# How fast can PPRV spread? Analyzing the results of an experimental PPRV infection of goats equipped with Ultra-Wideband sensors

**DOI:** 10.1371/journal.pone.0358839

**Published:** 2026-09-23

**Authors:** Manal Nhili, Jean-Baptiste Menassol, Gaye Laye Diop, Mariame Diop, Aminata Ndoye, Mbengué Ndiaye, Aminata Ba, Michel Dione, Assane Gueye Fall, Noha El Khattabi, Modou Moustapha Lo, Mounia Abik, Andrea Apolloni, Adama Diallo

**Affiliations:** 1 UMR ASTRE, CIRAD, Montpellier, France; 2 UMR ASTRE, CIRAD, University of Montpellier, Montpellier, France; 3 ENSIAS, Mohammed V University in Rabat, Rabat, Morocco; 4 UMR SELMET, CIRAD, INRAE, Institut Agro, Univ Montpellier, Montpellier, France; 5 LNERV, Institut Sénégalais de Recherches Agricoles, Dakar, Sénégal; 6 International Livestock Research Institute, West Africa Regional Office, Dakar, Senegal; 7 Faculty of Sciences, Mohammed V University in Rabat, Rabat, Morocco; Harvard University Archives: Harvard University, UNITED STATES OF AMERICA

## Abstract

Peste des Petits Ruminants (PPR) is a highly contagious disease affecting goats and sheep. The speed and extent of its spread depend on contact patterns and the virus’s intrinsic characteristics. To estimate its propagation speed and the role of inter-individual contacts, we conducted PPR virus transmission experiments in goats that were equipped with Ultra-Wideband (UWB) sensors to continuously track inter-individual distances alongside clinical and virological monitoring. Using Bayesian modeling, we integrated contact data and health status to estimate the basic reproduction number (*R*_*0*_), the incubation period, and the virus transmissibility. The novel integration of distance tracking revealed that animal density in an area critically drives infection risk: the minimum exposure time that is required for a naïve animal to become infected dropped from nearly two days at low density to just four hours at high density. Overall, the derived *R*_*0*_ estimates were 4.3 (95% HDI: 3.1–4.8) for a 5-day infectious period and 8.6 (95% HDI: 6.2–9.6) for a 10-day infectious period. The incubation period was estimated to be around 14.7 days (95% CI 11.6–20.1). The exposure time varies greatly depending on the density, ranging from nearly two days at low density to around four hours at high density. These findings highlight high-density environments, such as conditions in livestock markets, as an important factor of PPRV transmission between animals and the disease spread. Considering the high frequency of animals’ movement on long distances, the high incubation period, about 15 days, might be one of the factors explaining the spread of PPR along long distances from the source of infection. To efficiently control and eradicate PPR, sheep and goats’ movement control must be one of the means to be applied. Our findings, based on a single lineage IV PPRV strain (Senegal 20-GP) evaluated under experimental conditions, suggest that the adequacy of the current 21-day quarantine period, as specified in the World Organization for Animal Health (WOAH) *Terrestrial Code*, deserves further evaluation and validation under field conditions.

## Introduction

Peste des petits ruminants (PPR) is a highly infectious and contagious disease affecting small ruminants. Clinically, it is characterized by fever, congestion of different mucosal membranes, followed by ocular and nasal discharges, then buccal erosive lesions and diarrhea at the late stage with death in some cases. Because of its morbidity rate that can be as high as 80–90% and a mortality rate as high as 50–80% depending on the epidemiological context, PPR is in the list of economically important animal diseases in which outbreaks must be reported to the World Organization for Animal Health (WOAH). The causative agent of this disease is a virus, the PPR Virus (PPRV). It is one of the members of the genus Morbillivirus in the family *Paramyxoviridae,* along with the rinderpest virus, the causal agent of rinderpest, the cattle and buffaloes disease that was officially eradicated globally in 2011 [1,2]. Because PPR and rinderpest share similar symptoms (ocular and nasal discharges, buccal erosive lesions and diarrhea), PPR was overlooked for a long time in favor of rinderpest. But gene sequence data that were available as of 1994 had showed that PPRV and RPV are distinct viruses but closely related [3]. In fact, looking carefully at the epidemiological history, both diseases have evolved independently [4]. From its historical stronghold endemic region in West Africa, PPR has expanded to most of African countries, except Southern Africa, the Middle and Near East, and most of the Asian countries (from China to Central Asia), and then some European countries [5,6].

The regions that are currently threatened by PPR are home to about 80% of the world’s sheep and goat populations, and the annual losses that are caused by this disease were estimated in 2014 to be at the range of 1.2–2.8 billion [7]. Due to its significant economic impact, particularly on small farmers who rely mainly on sheep and goats for their livelihood in low-income countries, the Food and Agriculture of the United Nations (FAO) and World Organization for Animal Health (WOAH) convened an international conference in 2015 on PPR, conference during which was adopted a strategy for the global eradication of this disease by 2030: the PPR Global Control and Eradication Strategy (PPR-GCES) [8].

PPRV strains that have been identified so far have been grouped into four genetic lineages (I, II, III and IV) based on the virus gene sequence data [9]. Initially, these lineages occupied distinct geographical distributions across West Africa, East Africa and Asia. The lineage IV which is also named the Asian PPRV lineage as it was present only in Asia and the Middle East, but not in Africa, expanded its geographic distribution to that continent since year 2008, either replacing pre-existing lineage strains in some countries or co-existing with pre-existing lineage strains in some other countries [10–13]. For the moment, it is not known the factors behind the PPRV lineages geographical changes and the apparent advantages of lineage IV over to other lineages.

Knowledge of basic parameters of the PPRV transmission from host to host and understanding the virus spread might be useful in describing the transmission dynamics and supporting the PPR eradication program for more efficiency [14]. In 2018, Fournié et al. [15] proposed a mathematical model of PPRV transmission and elimination in Ethiopia based only on PPR serological surveillance data without any PPRV transmission experiment. Few years later, Herzog et al. [16,17] reported PPRV transmission experiments they conducted on both cattle and domestic small ruminants with a view to studying the potential involvement of cattle in PPR epidemiology. They concluded that cattle play a negligible role in PPRV circulation, even though PPRV-excreting small ruminants can infect cattle but without any overt disease. That transmission seems to have a probability much lower than that of small ruminants to small ruminants transmission. To our knowledge, there are currently no reports estimating PPRV transmission parameters in small ruminant populations under controlled experimental conditions. With that objective in mind, we conducted PPRV transmission experiments on goats to estimate basic epidemiological parameters, like contact rate and incubation period, as well as the minimum exposure time (i.e., the minimum time a naïve animal should be in contact with an infected one to get infected) and the influence of contact patterns between animals on virus transmissibility. Based on the results we obtained we propose a model for PPRV transmissibility. As the PPRV lineage IV seems to be the group of the most aggressive PPRV strains, continuously expanding its geographical distribution compared to other PPRV lineages, our experiments were conducted with a strain of that group. It was isolated during a PPR outbreak in 2020 in Senegal.

For our experiments, we equipped animals with UWB and Radio-frequency identification (RFID) devices that have been widely used to study interaction among individuals. Adapted to working in confined environments, this type of device provides more detailed and frequent information than GPS. The signals of the two devices are frequently triangulated, and the distance between their bearers is estimated at each time step. The result is a dynamic contact network among individuals. UWB and RFID devices have been widely used to study interactions among individuals (humans) in different settings like social events, conferences [18,19], hospitals [20], schools [21,22], and villages [19,23,24]. The results provide information for studying the interactions among individuals and retrieving information about possible drivers. Similarly, UWB devices have been recently applied to study animal behavior and welfare for different animal species, dogs, chickens, sheep, cows, and horses [25–33].

Due to the transmission mechanisms, we suppose that the likelihood of getting infected depends on the contact pattern between animals and could increase with exposure time. In our study, we tested these hypotheses by analyzing data from experimental infections where animals were equipped with UWB devices to collect information about contacts, and a set of statistical models was developed to infer the value of the virus’s transmissibility. The primary objectives of our study were: (i) to estimate the transmission rate and basic reproductive number ($R_{\text{0}}$) of the PPRV lineage IV; (ii) to characterize the distribution of the incubation period from experimental infection data; (iii) to estimate the minimum exposure time required for infection under different distance thresholds. Furthermore, we assessed whether UWB-derived distance information improved interpretation relative to duration-only models. To our knowledge, this is the first time that this kind of study, combining infection monitoring and contact patterns, has been performed for PPR. By taking account of contact variation, the analyses could help provide more accurate estimates of transmission probabilities and, consequently, the impact of PPR.

## Materials and methods

The study was conducted between June 2021 and June 2022. Five sessions of experiments were conducted during this period in June, August 2021, and January, April, and June 2022. In each session, two or three batches of six or seven animals were experimentally infected (exposure phase) and monitored. Four durations of the exposure phases were tested: 1 hour, 6 hours, 24 hours, and 44/48 hours. A total of twelve experiments were carried out for a total of 82 animals involved. The study was designed to detect 80% of the event of a transmission, if any, with a probability as low as 0.03. For the ease of the analysis, data from batches were classified using the session number (1–5), the batch number in each session (1–3), and the duration of the exposure phase (1h, 6h, 24h, 44h, 48h).

### Virus

In each experiment, a single animal (called the seeder) was infected with the PPRV Senegal 20-GP strain. The virus was isolated in BTS cells, a recombinant CV1 cell expressing the bovine SLAM protein (C. Adombi and A. Diallo, unpublished data), like the CHS cell which is a recombinant CV1 cell expressing the goat SLAM [34]. Both SLAM protein-based recombinant cells are efficient for PPRV in vitro isolation from pathological samples (C. Adombi and A. Diallo, unpublished data). This virus was grown in the BTS cells, and the collected virus suspension was aliquoted and stored in a 1.5 ml tube at −80°C. It was used at the 3rd passage in the BTS cells. The contents of three tubes were defrosted and titrated independently by the endpoint dilution assay on the BTS cells in 96-well flat-bottom plates for their cytopathic effect (CPE). The virus suspension titer was expressed as the 50% tissue culture infectious dose (TCID50), calculated according to the Spearmann-Kärber methodology as described by [35]. The mean value of the three titrations was 10^3.2^ TCID50/ml. It belongs to the PPRV lineage IV according to its partial Np sequence data [36]. In preliminary animal pathogenicity testing (Diallo A., Diop. M, Sagna A. and M. Lo, unpublished data), all goats inoculated with this virus exhibited acute PPR symptoms: high fever up to 40–41°C, diarrhea, significant ocular and nasal discharges, and death in four out of ten inoculated animals.

### Animals

A total of 82 goats, naïve and aged between six and twelve months, were used in the twelve experiments. They were purchased from different farms in Senegal by a team from the Laboratoire National d’Elevage et de Recherches Vétérinaires (LNERV) of the Institut Sénégalais de Recherches Agricoles (ISRA). Before purchase, all were proved negative for both PPRV and antibodies anti-PPRV by the ID.vet Innovative Diagnostic PPR pen-side test (ID Rapid® PPR Antigen) and the ID.vet Innovative Diagnostic ID Screen**®** PPR Competition respectively (See S1 File). The tests were performed by the ISRA laboratory team in the village of the farmers before purchasing the animals. After the purchase, the animals were brought to the ISRA experiment farm at Sangalkam (14.7955°N, 17.2289°W; WGS 84), about 30 km from Dakar. Upon arrival they were housed in a 300 m² barn for a minimum of two weeks for quarantine and acclimatization. During the first and second weeks, they were submitted to a second round of antibody anti-PPRV detection assay (ID.vet Innovative Diagnostic ID Screen® PPR) and the Reverse Transcription Polymerase Chain Reaction (RT-PCR) for PPRV nucleic acid detection according to the method of [37]. Only animals that tested negative again in both assays were included in the experiments. Any goat that was tested positive by one of the two tests was immediately removed from the group and excluded from the study.

### Experiment facilities

At the Sangalkam experiment farm, the experimental infection protocol was conducted in a dedicated and specially equipped area of 40m^2^ (see Fig S1, Section 1 in S1 File), located at about 50 meters from the quarantine barn. Within this area, paddock 2 was designated for controlled PPRV infection, while the animal-to-animal PPRV transmission experiments were conducted in paddock 1. Both open experimental barns were equipped with fenced pens designed to house each animal individually after the transmission experiments, to facilitate individual monitoring of PPR symptoms. The storage rooms were designated for storing animal feed and technical equipment, including syringes, blood collection tubes, gloves, coats, and containers for pathological sample collection. Outside the periods of experimental infection protocols, the animals were housed in the 300 m² barn. All barns, paddocks, and individual pens were equipped with troughs to provide animals with ad libitum access to feed and water. All the animal facilities were cleaned and disinfected before and after housing animals. During the experiment, the occupied rooms were cleaned daily.

### Experiment design

**Fig 1:**
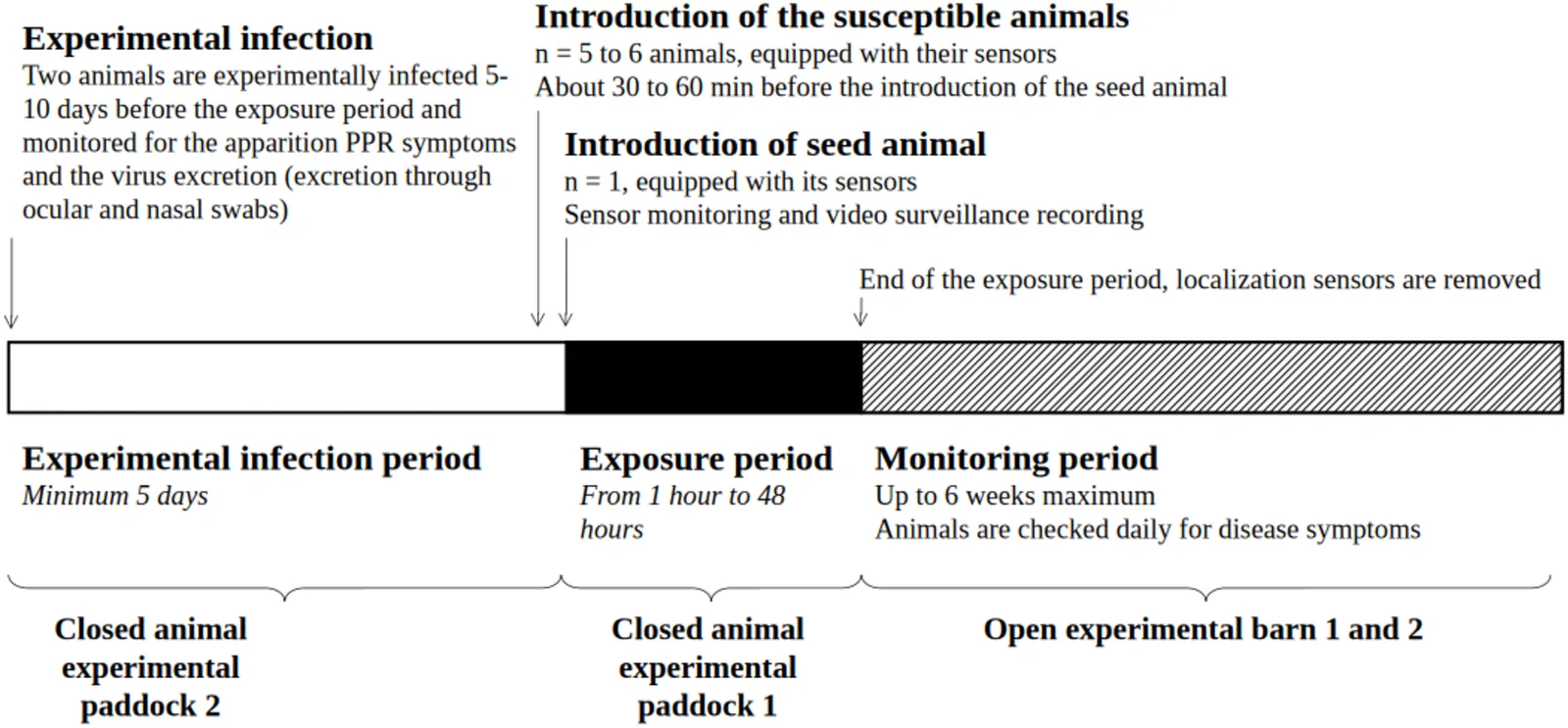
Detailed Schema of the Experimental Infection Protocol for Studying PPR lineage IV Transmission Dynamics. This diagram outlines the sequential stages of the experimental protocol designed to investigate the transmission of PPR among goats. The protocol includes three primary periods: the experimental infection period, the exposure period, and the monitoring period.

Each experience was divided into three phases as shown in Fig 1:

1. Experimental Infection phase: infection of animal to be used for transmission of the PPRV to naïve animals by contact: the seeder.
2. Exposure Phase: The seeder is in contact with other naïve animals to allow transmission from animal-to-animal in a paddock for a period of variable duration.
3. Monitoring phase: exposed animals are isolated and monitored until they show PPR symptoms or any other PPR test positive results.

Detailed procedures for each experimental phase are provided in the S1 File, Section 1.

### Data collection for indoor positioning

UWB solutions are used to measure the distance between a reference sensor (referred to as an anchor) and a sensor whose position is to be determined (referred to as a tag, equipped on the animal) using different metrics. For these experiments, paddock 1 was equipped with six anchors (UWB BeSpoon - STMicroelectronics, France) fixed to the ceiling, one at each corner and two near the center of the room. Each anchor was powered by a Power Over Ethernet cable connected to a dedicated 16-port switch (DES-1018MP; D-Link, Taipei – Taïwan). The tags (BeSpoon Industrial Tag; Length: 101 mm, Width: 52 mm, Thickness: 25 mm; BeSpoon - STMicroelectronics, France) were enclosed in a custom 3D-printed case to provide shock protection and ensure better attachment to the collar. The UWB system was controlled via a dedicated local server (a laptop with a Linux operating system), which provided a user interface for verifying the general system status and initiating data acquisitions. This experimental setup was designed to monitor and record, in near-real time, the precise indoor location of each individual.

The theoretical performance of the solution allows positioning an object with an accuracy of +/- 20 centimeters, but these performances depend notably on the tracking environment and the deployment conditions. Therefore, in order to evaluate the performance of this indoor positioning system under our experimental conditions, we designed and implemented a custom evaluation protocol using the different available tags (n = 7). Accuracy and precision were assessed using pairwise inter-sensor distance measurements across multiple geometric configurations:

- Sensors arranged in a straight line on the ground, with inter-sensor distances of 6, 10, 15, 30, and 50 cm, respectively.
- Sensors arranged in a straight line on a wooden bench at a height of 51.6 cm (approximating their actual monitoring height when equipped on the animals), with inter-sensor distances of 20 cm.
- Sensors arranged in a hexagon configuration with one sensor at the center and inter-sensor distances of 15, 30, and 100 cm, respectively.

Across all configurations, the system demonstrated a mean accuracy of 28.3 ± 6.2 cm and a mean precision of 22.2 ± 4.8 cm. These results were consistent with the manufacturer’s specifications and met our experimental requirements.

During the monitoring phase, the acquisition frequency was set to f = 1 Hz (one acquisition per second). Data were stored on the server as a csv file, with each line representing the estimated position of each tag on the x, y, and z axes. Distances between the infected individual and each susceptible animal were then computed from these data, using only the x and y axes. This frequency of indoor positioning provided a comprehensive temporal map of animal interactions. High-resolution proximity data enabled us to analyze not only the frequency of close contacts, but also the duration of these interactions at different distance thresholds.

### Ethics statement

Animals were monitored daily for clinical signs. Because severe diarrhea is a frequent precursor to mortality in PPRV-infected animals, the onset of this clinical sign was established as the humane endpoint for euthanasia. Euthanasia was performed via intravenous injection of 25 mL of pentobarbital sodium (PENTOBARSOL™), a method that induces rapid and painless death, thereby minimizing distress. For biosecurity purposes, at the conclusion of each experiment, all remaining animals were humanely euthanized using this same protocol, and the facilities were thoroughly cleaned and disinfected.

All the animal experiments have been carried out by staff of the LNERV/ISRA at the ISRA experimental farm. LNERV/ISRA is the Senegalese national veterinary laboratory for diagnostics and research in animal health and production. One of the supervisors of the experiments, Mr. Adama Diallo, DVM and PhD, has about 40 years’ experience in animal viral disease vaccine development with testing in animals. The second, Mr. Moustapha Lo, head of the Animal Health Program at LNERV, at the time of the experiment, is responsible for biosecurity and biosafety at ISRA/LNERV. The procedures and protocols of the animal experiment which results have been reported here were submitted to and approved by the Scientific Direction of LNERV/ISRA, a competent body for animal research.

### Statistical analysis and models

We employed a Bayesian statistical framework to estimate the transmission rate, incubation period, and model transmission probability, a choice that was driven by the need to integrate high-resolution individual proximity data with batch-level and individual level infection outcomes. This probabilistic approach is particularly suited to experimental datasets with a limited number of independent batches and small sample size, as it avoids reliance on large-sample asymptotic assumptions and it allows for the rigorous quantification of uncertainty through posterior distributions. By employing a Bayesian structure, we defined model-specific priors to ensure biological plausibility. Depending on the particular quantity to estimate, we used different likelihoods to characterize the binary outcome of infection status and the duration of the incubation phase. In all cases we used minimally informative prior distributions to minimize potential bias and applied the Markov Chain Monte Carlo (MCMC) approach to estimate model parameters. Table 1 summarizes the four transmission probability models described in this section, detailing their respective outcomes, predictors, parameters, and epidemiological assumptions.

**Table 1:** Comparative roadmap of transmission probability models.

Model	Analysis Class	Predictors	Outcome	Likelihood	Parameters	Epidemiological Interpretation
Experiment (Batch)	Baseline (Null)	Total exposure duration	Batch infection count	Binomial	p	Risk is constant and independent of inter-animal distance
Individual (goat)	Segmented	Time spent within distance threshold	Individual infection status (0/1)	Bernoulli	p	Risk is binary; transmission occurs only within a fixed radius
Individual (goat)	Stratified	Durations per distance band	Individual infection status (0/1)	Bernoulli	p and $\alpha_{i}$	Risk decays step-wise across discrete distance intervals
Individual (goat)	Envelope	Cluster median distance (r) and duration	Individual infection status (0/1)	Bernoulli	$p_{\text{0}}$ and λ	Risk decays exponentially as a continuous function of distance

#### Infection classification and endpoint.

To ensure consistency across all Bayesian models, we defined a pre-specified infection classification rule. An individual was classified as “infected” (Outcome = 1) if it met at least one of the following criteria during the monitoring phase: (1) seroconversion as detected by competitive ELISA (cELISA), representing a robust immune response to exposure; (2) a positive RT-PCR result from nasal/ocular swabs, indicating active viral shedding; or (3) positive post-mortem PCR results from lymph or lung tissue in cases of mortality. While seroconversion involves a temporal lag of several days, we used a binary Bernoulli likelihood that is independent of this lag. In this way, we compute the probability of a transmission event occurring strictly within the fixed exposure window (1–48 hours). Thus, the temporal delay in antibody detection affects the incubation period analysis but does not impact or bias the estimation of the unit-time transmission probability (p) nor the resulting minimum exposure time simulations.

#### Transmission rate.

We estimated the hourly transmission rate $\beta_{h}$ of the strain following the procedure of Dekker et al. [38]. The infection outcome for each susceptible individual in an interval of time Δt modeled as an independent and identically distributed Bernoulli random variable with infection probability *p*_*t.*_


$$\begin{array}{c} p_{t}\;\;=\;\text{1}\;-\;e^{-\beta_{h}\;\frac{I_{t}}{N_{t}}\;\Delta t\;} \end{array}$$ (1)

Where *N*_*t*_ is the total number of animals in the pen/paddock, $I_{t}$ the number of initially infected individuals (1 in all cases) and Δt is the duration of the exposure phase ranges from 1 hour to 48 hours (Δt = 1, 6, 24, 44, 48). Due to the variation in the length of the exposure phase, we estimated $\beta_{h}$ the transmission rate per hour. We estimated $\beta_{h}$ using the No-U-Turn Sampler (NUTS), fitting the model to serological data using a binomial likelihood.

#### Basic reproductive number.

In a continuous transmission model, considering an exponential process, the daily transmission rate $\beta_{d}$ can be estimated from the hourly transmission rate $\beta_{h}\;$as:


$$\begin{array}{c} \beta_{d}=\;\text{1}\;-\;e^{-\beta_{h}\;\text{2}\text{4}} \end{array}$$ (2)

Based on this, a first derived estimate of the basic reproductive number $R_{\text{0}}$ was calculated using the following formula derived from the SIR model definition:


$$\begin{array}{c} R_{\text{0}}=\frac{\beta_{d}}{\gamma } \end{array}$$ (3)

where, $\beta_{d}$ is the daily transmission rate, and γ stands for the recovery rate (the inverse of the infectious period) commonly used in PPR modeling. The infection period could not be estimated from our experimental settings. Therefore, we calculated $R_{\text{0}}$ using two infectious period estimates from the literature (5 and 10 days).

#### Incubation period estimation.

Following the same procedure that was adopted by Miura et al. [39], we estimated the incubation period by fitting a parametric distribution to the animal follow-up data. In order to estimate the incubation period, one of the following information were taken into consideration for each animal when available: the day on which symptoms were first observed, the day on which a positive result was obtained from the PCR test (indicating the presence of the virus), and the day on which a positive result was obtained from the serology test.

We used data on the time at which symptoms first appeared, or on positive RT-PCR results, to estimate the incubation period. After a preliminary analysis to check if exposure duration could impact the length of the incubation period survival analysis was conducted to estimate the average incubation period disregarding categorization in exposure groups.

To improve the estimation of the incubation period, we also included seropositive-only animals. A survival model was calibrated under the assumption that the onset of symptoms preceded seroconversion by a short and variable delay. This delay was modelled using a normal distribution parametrized using data from five symptomatic animals (mean = 2.3 days, standard deviation = 2.9 days). Model calibration was performed using ten Markov chains of 5000 iterations each.

To take account of the uncertainty of the moment of infection occurrence during the moment of exposure phase, this was randomly extracted for each animal. Three typical parametric distributions used in survival analysis (Weibull, lognormal, and Gamma) were used for likelihood and chose the one with the lowest widely applicable information criterion (WAIC).

#### Transmission probability.

We studied the effect of cluster dependence and within-experiment correlation on infectious status of the animals.

We used logistic regression model with a random effect, using the predicted variable as the individual infectious status and the exposure measure (${exposure}_{j})$ as a predictor of infectious status.


$$\begin{array}{c} \text{log}\left(p_{ij}\right)=\beta_{\text{0}}+\beta_{\text{1}}\;exposure_{j}+b_{j}+\epsilon_{ij} \end{array}$$ (4)

Exposure was considered to be the inverse of the distance from the source of infection at each time point, and we considered both the cumulative exposure and the duration of the experiment. We then performed a comparison between the different models using ANOVA test, to find if any difference existed between the different experiments.

**Fig 2:**
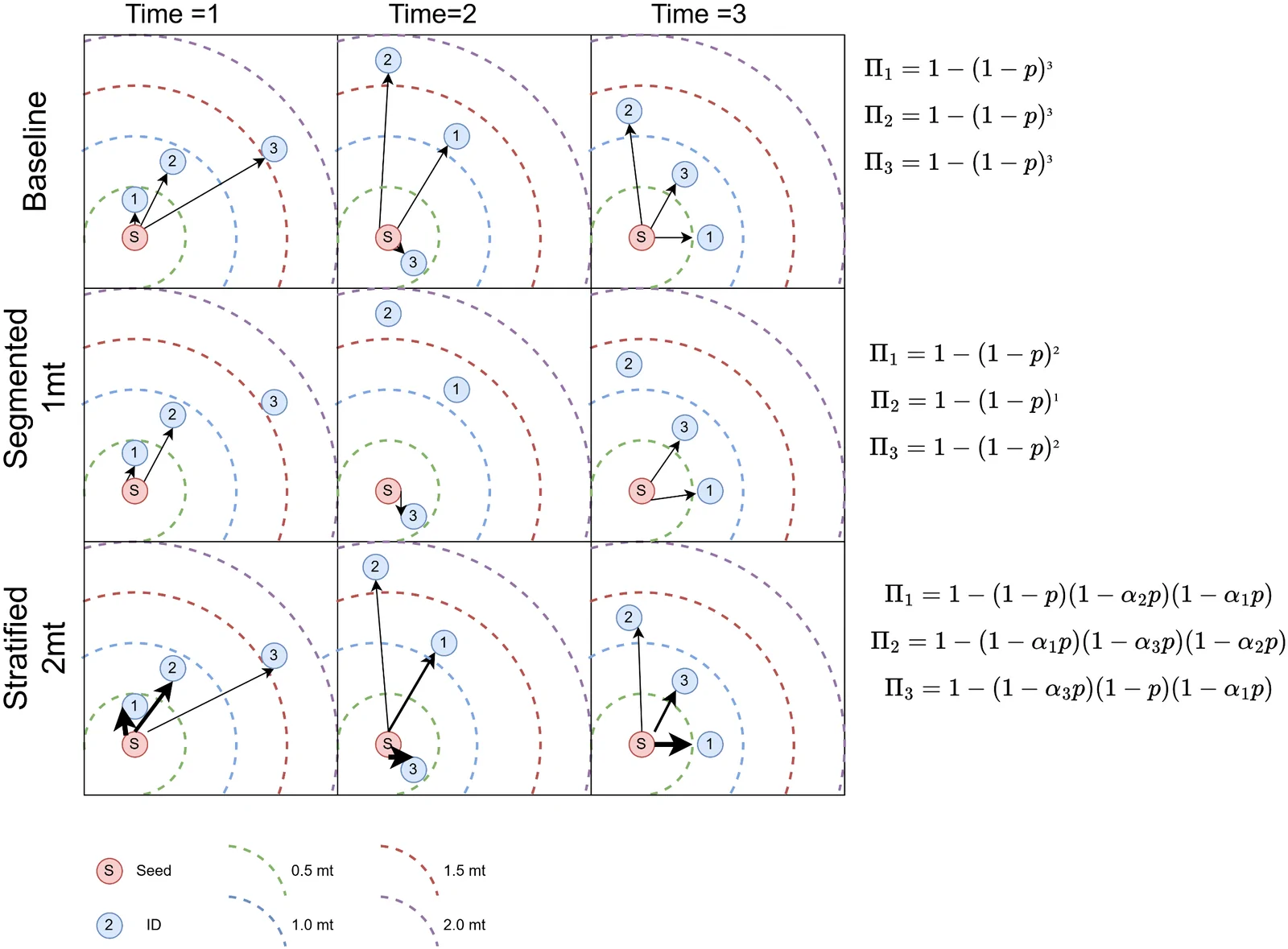
Graphical representation of three different models (Baseline, Segmented, and Stratified) illustrating the spread of infection over time. The infected seeder individual, denoted as S, interacts with susceptible individuals (1), (2), and (3). The arcs represent the varying distances between S and the susceptible individuals at three time points: t = 1, t = 2, and t = 3 (in minutes). The circles depict the dynamic radius of interaction, which either increases or decreases over time, showing the changing proximity and potential for infection spread in all orientations. The envelope model is a modification of Stratified model, where ranges are determined based on animals’ movement patterns.

Our analytical approach comprised four distinct modeling strategies for PPRV transmission in our study: (i) baseline model, (ii) segmented model, (iii) envelope model, and (iv) Stratified model. In our Bayesian statistical approach, we initially developed a baseline model that intentionally excluded proximity factors, focusing solely on temporal exposure patterns (see more details in Fig 2). In this approach we characterized transmission dynamics without spatial considerations. Subsequently, we progressively incorporated proximity data from UWB to systematically evaluate whether distance information significantly enhances transmission risk prediction. By comparing models with increasing spatial complexity, from a null temporal model to models integrating precise inter-animal distances, we aimed to empirically determine the statistical significance of proximity in PPRV transmission mechanisms. In all these models, we used Bernoulli likelihood to consider individual differences in contact patterns and activity.

##### Baseline model.

Our baseline model, which serves as the null hypothesis, assumes a constant probability of transmission per unit of time, independent of proximity. The individual cumulative probability of infection P for this model is expressed as follows:


$$\begin{array}{c} P=1-{\left(1-p\right)}^{t} \end{array}$$ (5)

where p represents the probability of a successful transmission per unit time, and t the total duration of the experiment. The simplicity of this model provided a fundamental benchmark against which more complex models could be compared.

##### Segmented model.

Building on the baseline approach, our segmented model has incorporated distance-based stratification to account for the potential influence of proximity on the virus transmission probability. This model uses the same fundamental equation as the baseline model:


$$\begin{array}{c} P\;=\;1\;-{\left(1-p\right)}^{t_{i}} \end{array}$$ (6)

However, in this context, $t_{i}$represents the cumulative time spent within specific distance thresholds (1 m, 1.5 m, and 2 m). Separate models were fitted for each threshold, enabling us to discern how the probability of transmission might vary with different distances.

##### Stratified model.

Our stratified model represents a more subtle method of integrating transmission dynamics as a function of distance. The observed distances were divided into different ranges:

- *t*_*1*_: cumulative time spent between 0.05 m and 0.5 m,
- *t*_*2*_: cumulative time spent between 0.5 m and 1 m,
- *t*_*3*_: cumulative time spent between 1 m and 2 m,
- *t*_*4*_: cumulative time spent beyond 2 m.

The probability of infection for this model is given by:


$$\begin{array}{c} P\;=\;\text{1}\;-\;(\text{1}-p{)}^{t_{j}}\;\Pi_{\text{i}}{\;\left(\text{1}-\alpha_{i}\;p\right)}^{t_{i}}\;,\;i\;\neq \;j \end{array}$$ (7)

where p is the probability of success, $\alpha_{i}$ are scale factors for different distances, and $t_{i}$, $t_{j}$ represents the time spent in each range. This formulation allows for a differential weighting of transmission risk according to proximity. We studied five variants of this model, each emphasizing different distances:

- Stratified 1: *j* = 1, *i* = 2, 3, 4
- Stratified 2: *j* = 1, *i* = 2
- Stratified 3: *j* = 3, *i* = 4
- Stratified 4: *j* = 2, *i* = 3, 4
- Stratified 5: *j* = 1, *i* = 2,3

##### Envelope model.

Our most sophisticated approach, the envelope model, integrates both temporal and spatial proximity data clustering. We identified distinct envelop interaction groups for each individual and used the total duration of these groups along with their median distances to estimate infection probability. This model was applied with different distance filters (≤ 1 m, ≤ 1.5 m, and ≤ 2 m). The mathematical formulation is detailed in (S1 File, Section 3).

#### Exposure time estimation.

To estimate the minimum exposure duration required for a susceptible animal to become infected, we simulated various contact scenarios by analytically inverting the cumulative probability of infection functions derived from our best-fitting transmission models. The cumulative probability of infection P(t) after an exposure time t (measured in minutes) was modeled under two main assumptions regarding animal movement and spatial density:

First, to represent a scenario where animals move freely and interact at varying distances without extended close contact (e.g., roaming herds), we used the Baseline model (see Eq. (5))

Second, to represent high-density environments where animals are maintained at relatively fixed, close distances (e.g., crowded livestock markets or transport), we used the Stratified models. For a susceptible animal kept at a constant distance within a specific spatial interval k, the cumulative probability of infection simplifies to:


$$\begin{array}{c} P(t)=\;\text{1}\;-\;{\left(\text{1}-\alpha_{k}p\right)}^{t} \end{array}$$ (8)

In this formulation, p represents the reference transmission probability for the baseline distance interval j and scaling factors $\alpha_{k}$.

For both scenarios, the minimum exposure time t required to reach a specific probability of infection threshold P (e.g., 50% and 95%) was calculated by solving the cumulative probability equations.


$$\begin{array}{c} t\;=\;\frac{\text{ln}(\text{1}-P)}{\text{ln}\left(\text{1}-p_{eff}\right)} \end{array}$$ (9)

Where $p_{eff}$ is the effective transmission probability per minute ($p_{eff}=\;p$ for the Baseline model, and $p_{eff}=\alpha_{k}p$ for the Stratified models).

We used the posterior mean and 95% HDI estimates of the transmission probabilities (p) and scaling factors ($\alpha_{k}$) obtained from the Bayesian inference of the models. We used the Baseline model to estimate exposure time for the unrestricted movement scenario. For fixed distances, we used the Stratified 1 model for the 0.05–0.5 m range, and the Stratified 4 model for the remaining distances (0.5–1 m, 1–2 m, and ≥ 2 m). Finally, we converted all exposure times from minutes to hours to make them easier to compare across the different density scenarios.

### Parameter estimation

The choice of prior distributions for model parameters was guided by both biological plausibility and computational considerations. The specific parametrizations of the priors were selected based on preliminary analyses and expert knowledge of PPRV transmission dynamics. Since individuals could vary based on their activity patterns, we used a binomial likelihood to elicit the differences among individuals’ patterns.

We used the NUTS, an MCMC method. This approach facilitated efficient exploration of the parameter space and estimation of posterior distributions. Our sampling protocol was designed to ensure thorough exploration of the parameter space. We configured the sampler to draw 10,000 samples per chain, with a tuning phase of 1,000 iterations, and ran ten independent chains. This configuration allowed for comprehensive sampling and robust convergence diagnostics. We chose a high target acceptance rate of 0.95 to promote efficient exploration of parameter space, which was particularly important for our more complex models. Posterior distributions were obtained for each model parameter, and the Bayesian estimates were summarized using the posterior mean estimate and the 95% highest (posterior) density interval (HDI). The 95% HDI is the minimum Bayesian credible interval (BCI) that contains 95% of the posterior probability distribution. Convergence of the chains was confirmed by visual inspection of trace and density plots and, the Gelman—Rubin diagnostic (R̂) which compares the within-chain and between-chain variances [40]. If the chains have converged, the variances should be similar and results in an R̂ close to 1.

### Model evaluation

Model comparison was facilitated through the computation of WAIC [41] because it provides an estimate of predictive accuracy while accounting for model complexity, without requiring cross-validation or separate training/test datasets. WAIC estimates how well a model generalizes to unseen data by balancing goodness of fit (how well the model fits the observed data) and penalizing complexity (to avoid overfitting). A model with a lower WAIC value is preferable, as it indicates better predictive performance. However, models with close WAIC values can be considered as having similar performance, and other factors such as interpretability and model simplicity can be considered for the final selection.

We conducted a receiver operating characteristic (ROC) curve analysis and generated areas under the curve (AUCs) estimates using the individual transmission probability for each model to compare their binary classification performance. The ROC curve is a graphical representation that plots the True Positive Rate (TPR) against the False Positive Rate (FPR) across all possible thresholds. The AUC values range from 0 to 1, with the higher AUC values signifying superior performance of the predictive model.

We assessed the goodness of fit through posterior predictive checks using aggregated Bernoulli draws at the experiment level. This approach involves simulating replicated datasets by drawing from a Bernoulli distribution and then summing up these binary classifications to compute the total number of positive outcomes. We compared the mode of the simulated aggregates to the observed number of positive cases for each experiment graphically.

The Bayesian analyses were performed using the MCMC algorithm and were conducted in Python (version 3.9) using the PyMC language model (version 5.10.3) with BlackJax backend (version 1.1.0). WAIC computations were handled using the built-in function from Arviz library (version 0.17.0). The statistical tests were conducted using R (version 4.4.2).

## Results

### Observed contact distribution and patterns

In our analysis, we considered only those experiments for which we had a complete dataset covering the entire duration of the experiment.

Fig S2-A (S1 File, Section 2) shows the distance distribution among animals for all experiments. From a visual inspection, the distance distribution appears different between experiments, which is confirmed by the Kruskal-Wallis test results ($\chi^{\text{2}}$ = 1331181, p-value < 2.2e-16) indicating that some of the distributions are different, and the *post-hoc* pairwise Wilcox test, indicating that there is a significant difference among all the experiments (all p-value < 2.2e-16) except two (namely the experiments 1_2 and 2_2) for which the corresponding p-value = 0.19.

Fig S2-B (S1 File, Section 2) summarizes the distribution in the form of a boxplot. As we can notice, the median distance in each experiment varies. In particular, we notice that for five of them the median is in the range 1.5–2.5 m., while for the others it is between 3.3 and 4.5 m. The differences among contact patterns could indicate different behavior of the animals, due to external reasons, and could influence transmission of the virus. We analyzed the temporal network to identify possible eras, i.e., specific periods related to different collective behaviors that could indicate the presence of circadian activity. In Fig S3 (S1 File, Section 2), the median (solid line) and the minimum and maximum distances among animals are reported for each second of the exposure phase. The era identification analysis shows inconclusive results: a single large period was identified, lasting more than 90% of the time, together with some very short eras (of the order of the minutes).

Instead of focusing on the characteristics of the overall network, we extracted information about the contact pattern between the seeder and each susceptible animal (called proximity analysis). The proximity results presented in Fig S4 (S1 File, Section 2) shows the fraction of time susceptible animals spent in very close contact (less than the 0.5 m), medium range contact (0.5–1.0 m), long range (1.0–2.0 m) and distant contact (more than 2.0 m) The pattern of dynamic behavior is characterized by frequent, spontaneous fluctuations, where susceptible individuals intermittently reduce their proximity to the seeder before increasing the distance again. Specifically, susceptible individuals would briefly enter closer proximity zones (under 1 meter) before returning to the mostly distancing pattern of beyond 2 meters. Overall, individuals spend a very small fraction of time in close contact with the seeder, while most of the time they are at a large distance.

### Clinical observations and serology results

In this section, we present the results of the tests and observations conducted to assess the animals’ health status and to determine whether they were infected (through RT-PCR), as well as their immune response to the exposure to the pathogen (through serological testing). The data collected from each experiment, including the monitoring period and the number of positive serological results, are summarized in Table 2. Overall, 18 positive cases based on positive serology were identified out of 70 susceptible individuals. A significant association between exposure time and infection status was found (Fisher Exact test, p-value < 0.001), indicating that exposure duration influences infection likelihood. Three of these (ID 32L, 42L and 81) were found to be positive during post-mortem analysis, while the others exhibited an immune response (positive serology) during the monitoring period. Only five animals (ID 104, 108, 99, 4 and 4D) were also positive in the RT-PCR test, and of these, only three (ID 104, 108 and 99) exhibited symptoms such as nasal and ocular discharges, diarrhea. At this last symptom, 4 animals were humanely euthanized (see Table 3).

**Table 2:** Summary of experimental infection results by experiment session and duration.

Experiment	Duration	Date	Batch size (In-contact animals)	Positive
1	1 h	27/06/2021	6	0
	6 h	27/06/2021	6	0
	24 h	27/06/2021	6	0
2	24 h	25/08/2021	6	0
	24 h	26/08/2021	6	1
	44 h	27/08/2021	6	4
3	24 h	02/03/2022	5	1
	24 h	02/03/2022	5	3
4	24 h	28/04/2022	6	0
	48 h	29/04/2022	6	4
5	24 h	14/06/2022	6	1
	48 h	15/06/2022	6	4

We report the number of newly infected animals (Positive) after the exposure phase.

**Table 3:** Day of symptom onset, RT-PCR positivity, serology positivity, and death/humanely euthanized for the confirmed cases of PPR by experiment and ID, as the number of days elapsed since the end of the exposure phase.

Session	Batch	Starting date	Contact duration	Animal Id	Symptoms	RT-PCR	Serology	Death/humanely euthanized (*)
2	2	26/08/2021	24 h	40			20/09/2021	
	3	27/08/2021	44 h	104	04/09/2021	06/09/2021	10/09/2021	13/09/2021* (17 days post exposure)
		27/08/2021	44 h	108	08/09/2021	08/09/2021	12/09/2021	
		27/08/2021	44 h	128			20/09/2021	
		27/08/2021	44 h	99	04/09/2021	07/09/2021	06/09/2021	07/09/2021* (11 days post exposure)
3	1	01/03/2022	24 h	20			15/03/2022	18/03/2022* (17 days post exposure)
	2	02/03/2022	24 h	32L				14/03/2022
		02/03/2022	24 h	42L				13/03/2022
		02/03/2022	24 h	23KD			16/03/2022	20/03/2022*(18 days post exposure)
4	2	29/04/2022	48 h	4		18/05/2022		18/05/2022 (19 days post exposure)
		29/04/2022	48 h	4D		16/05/2022		16/05/2022 (17 days post exposure)
		29/04/2022	48 h	16			15/05/2022	
		29/04/2022	48 h	41L			07/06/2022	
5	1	14/06/2022	24 h	6			06/07/2022	
	2	15/06/2022	48 h	18			24/06/2022	
		15/06/2022	48 h	33			08/07/2022	
		15/06/2022	48 h	59			24/06/2022	
		15/06/2022	48 h	81				

Symptoms indicate the day when at least one of the PPR symptoms (ocular, nasal discharge, diarrhea, high temperature) was recorded, virology the day when samples were positive to the lab test, serology the day when antibodies were detected in the blood, and death the day animals were found dead and positive to the disease.

**Fig 3:**
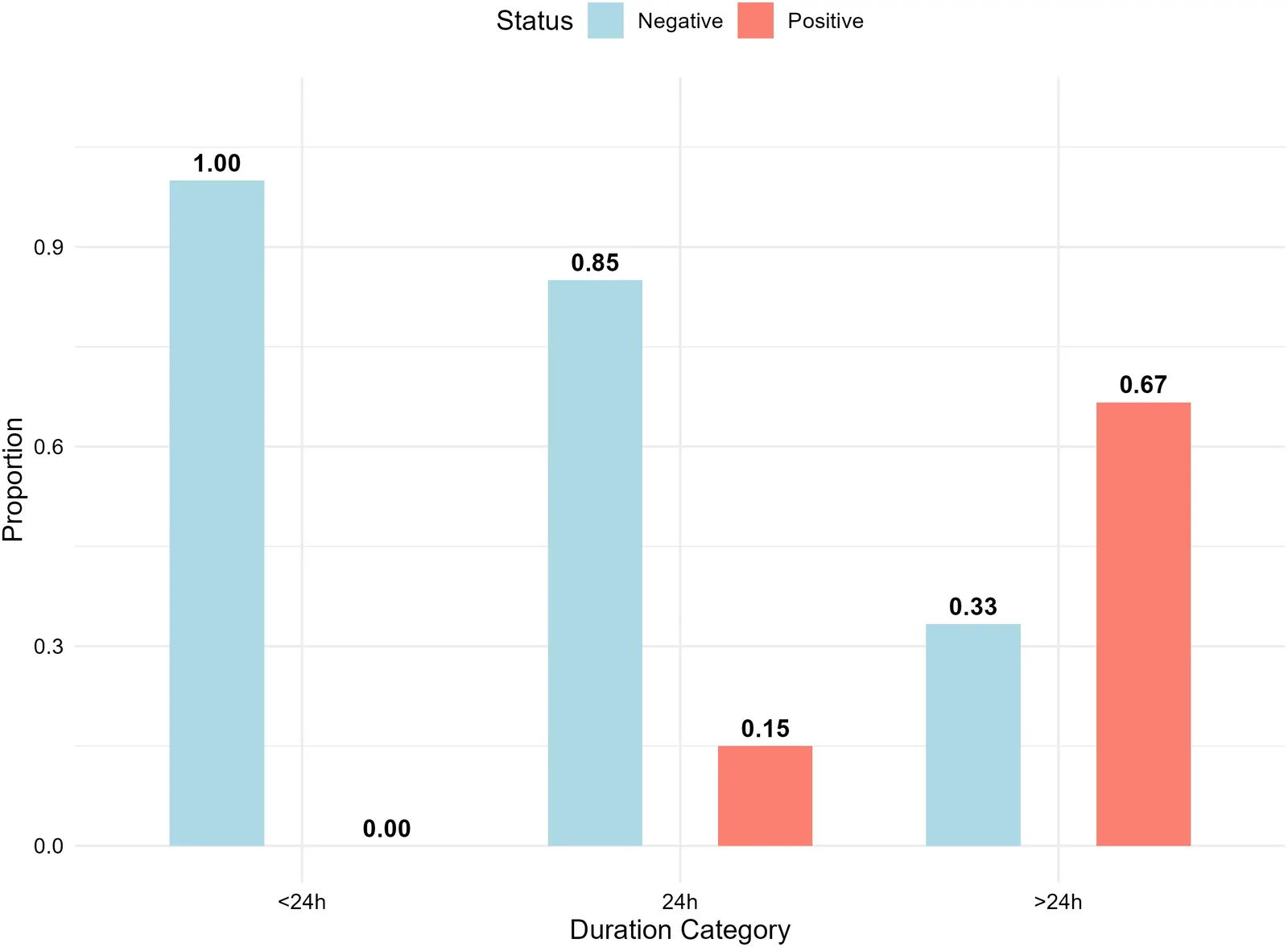
Proportions of infection by exposure time categories. Outputs of the experimental infections are grouped based on the duration of the exposure phase. Red corresponds to the fraction of positive in each category, Cyan to the negative ones.

Fig 3 visually depicts the proportions of infected versus non-infected individuals across different exposure time categories, emphasizing that no infections were recorded for exposure times under 24 hours. We observed that experiments with longer durations of exposure resulted in a higher number of infections, indicating that the length of the exposure period may impact the probability of transmission.

### Transmission rate estimation

The transmission rates were initially estimated on an hourly basis. However, for reporting purposes, these values were multiplied by 24 to convert them into daily rates. The estimated value of $\beta_{d}$ is 0.86 (95% highest density interval (HDI): 0.62–0.94).

The basic reproductive number $R_{\text{0}}$ is estimated to be 4.3 (95% HDI: 3.1–4.8) when considering an infectious period of 5 days as used by El Arbi et al. [42] and 8.6 (95% HDI: 6.2–9.6) when considering an infectious period of 10 days, as reported by Fournié et al. (2018) [15] and Herzog et al. (2024) [17].

### Incubation period estimation

Table 3 shows, for each positive confirmed animal, the day at which it showed symptoms (symptoms), was positive to RT-PCR or serological tests, and in case of deaths, the day of death. For the survival analysis, we considered the first day on which one of these factors was registered. As the table shows, very few positive animals (only 5) showed symptoms or were positive to RT-PCR during the follow-up period, indicating that the virus may circulate sub-clinically in a herd. This occurred an average of 11 days after exposure, with a range of 7–17 days. The same animals tested seropositive around two days later on average (2.3 days). Animals that did not exhibit symptoms became seropositive within an interval of 9–37 days after exposure phase.

Preliminary survival analysis was done to check if there was a difference between exposure duration and length of the incubation period. The log-rank test showed no significant difference between the groups (Chi-square 0.1, p-value 0.8). Because of this, we conducted survival analysis without taking into consideration the duration of the exposure phase. Among the three parametric models, the LogNormal one outperformed the others (Table 4). The average incubation period is around 14.7 days (95% CI 11.6–20.1).

**Table 4:** Estimation of the incubation period using three different parametric distributions (Weibull, Gamma, and LogNormal) showing the median of the parameter estimates, the 95% Credible Interval and Watanabe Akaike Information Criterion (WAIC).

	2.5%	50%	97.5%	WAIC
Weibull	11.6	15	19.2	116.5
Gamma	11.7	14.4	17.9	114.8
LogNormal	11.6	14.7	20.1	113.1

**Fig 4:**
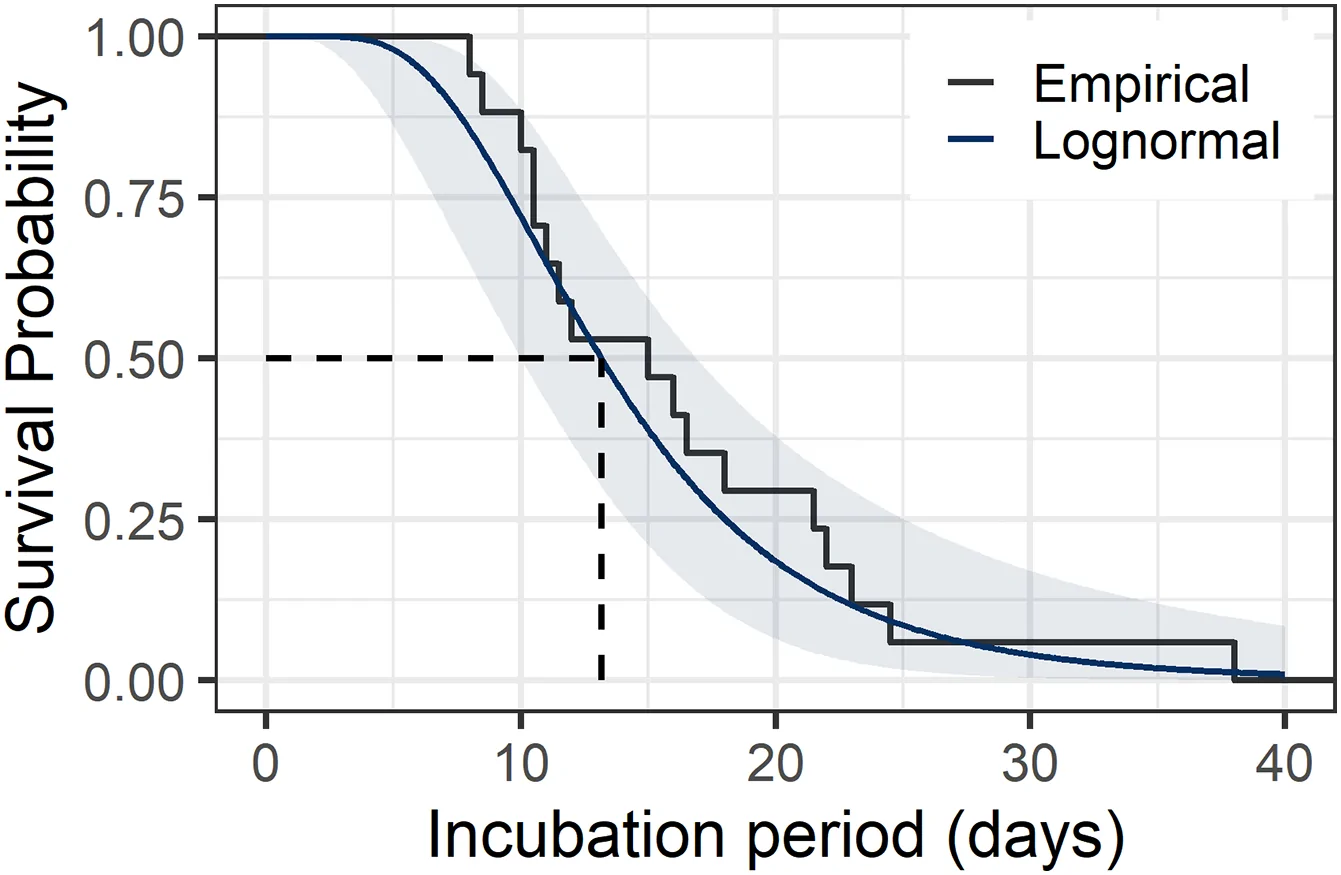
Kaplan-Meier curve for the empirical (black solid line) and the estimated one (blue). The shaded area corresponds to the 95% C.I. of the survival plot. The dashed line indicates the time (in days) half of the population has become infectious.

Fig 4 shows the empirical Kaplan-Meier curve, and the theoretical one obtained using a lognormal distribution. During the first four days after exposure, none of the animals can secret the virus. From the fifth day till the 20^th^ day, the probability of not developing symptoms decreases. Around the 14^th^ day, half of the population is no longer incubating, and animals either show symptoms or are tested positive. After the 22^nd^ day, only a small percentage of animals still develop symptoms.

### Transmission probability estimates

The preliminary analysis using logistic model results implies a significant effect of the group on the infectious status indicating that there could be actually a difference in the outcomes that could depend on the exposure/contact pattern (See S1 File, Section 6).

In Table 5, an overview of the prior distributions used for each parameter across all models is presented. For the transmission probability parameters, we employed Beta distributions to constrain values between 0 and 1, reflecting the probabilistic nature of these parameters. The decay parameter in the envelope model was assigned a Gamma distribution to ensure positivity while allowing for a range of plausible values. For scaling factors in the Stratified model, we used a uniform prior over a range that encompassed biologically reasonable values.

**Table 5:** Prior distributions for model parameters.

Model	Parameter	Assumption	Prior Distribution	Justification
Baseline	P	Risk is constant and independent of inter-animal distance	Uniform(0.0001, 0.01)	Uninformative prior for probability
Segmented	P	Risk is binary; transmission occurs only within a fixed radius.	Uniform(0.0001, 0.01)	Uninformative prior for probability
Stratified	P	Risk decays step-wise across discrete distance intervals	Beta(1, 1)	Uninformative prior for probability
	$\alpha_{i}$		Uniform(0, 1)	Allows for range of scaling effects
Envelope	$p_{\text{0}}$	Risk decays exponentially as a continuous function of distance	Beta(1, 1)	Slightly informative, centered at 0.5
	λ		LogNormal(−1, 0.1)	Ensures positivity, allows for a range of decay rates

The estimated probability parameters p from MCMC, differ across models depending on how they incorporate distance. The results of the inference through calibration are shown in Table 6. Probability estimates for the Baseline case are the lowest. This is mainly due to the fact that in the model, individuals are considered always to be exposed independently of the distance.

**Table 6:** Summary of posterior distributions for all models.

Model	Parameter	Assumption	Mean	SD	HDI 95% lower	HDI 95% upper
Baseline	p	Risk is constant and independent of inter-animal distance	0.0003	0.0001	0.0001	0.0004
Segmented 1 m	p	Risk is binary; transmission occurs only within a fixed radius.	0.0063	0.0014	0.0037	0.0092
Segmented 1.5 m	p		0.0028	0.0007	0.0015	0.0041
Segmented 2 m	p		0.0014	0.0003	0.0008	0.0021
Envelope 1 m	$p_{\text{0}}$	Risk decays exponentially as a continuous function of distance	0.0112	0.0029	0.0059	0.0169
	λ		0.3702	0.0374	0.297	0.4428
Envelope 1.5 m	$p_{\text{0}}$		0.0043	0.0011	0.0023	0.0063
	λ		0.3703	0.0371	0.2999	0.4439
Envelope 2 m	$p_{\text{0}}$		0.0025	0.0006	0.0014	0.0038
	λ		0.3706	0.0372	0.2999	0.445
Stratified 1	p	Risk decays step-wise across discrete distance intervals	0.0046	0.0043	0.0003	0.0133
	$\alpha_{\text{1}}$		0.3764	0.2708	0.014	0.9012
	$\alpha_{\text{2}}$		0.5064	0.2627	0.1092	0.9973
	$\alpha_{\text{3}}$		0.5879	0.2524	0.1586	0.9999
Stratified 2	p		0.0117	0.0063	0.0036	0.0245
	$\alpha_{\text{1}}$		0.5855	0.2427	0.1835	0.9996
Stratified 3	p		0.0006	0.0003	0.0002	0.0014
	$\alpha_{\text{1}}$		0.4917	0.2644	0.0869	0.9895
Stratified 4	p		0.0016	0.0011	0.0002	0.0038
	$\alpha_{\text{1}}$		0.41	0.266	0.0264	0.9192
	$\alpha_{\text{2}}$		0.5531	0.2603	0.1281	1
Stratified 5	p		0.0064	0.0049	0.0011	0.0163
	$\alpha_{\text{1}}$		0.4705	0.2621	0.061	0.9486
	$\alpha_{\text{2}}$		0.5866	0. 2476	0.1693	1

For the other models, spatial information was incorporated *post hoc,* by applying distance-based filters to the proximity data after it was collected; no constraints were imposed during data generation or experimentation. Segmented models used simple distance filters (e.g., allowing transmission only within 1 m, 1.5 m, or 2 m) produced progressively lower p estimates (*p* = 0.0063, 0.0028, and 0.0014, respectively), showing that increasing the spatial window reduces the estimated per-individual transmission probability. Stratified models further refined this approach by dividing the spatial range into intervals (e.g., 0.05–0.5 m, 0.5−1 m, 1–1.5 m, etc.), and we noticed that the more models cover close-contact (0.05–0.5 m) the more the probability p estimates increase (e.g., Stratified 2, Stratified 5 and Stratified 1), and for the case when models integrated distances above 2 m, the p estimates decrease (e.g., Stratified 3). In particular, we noticed that for Stratified 2 model the probability of transmission at very close distance (less than 0.5 m) is 39 times larger than in the baseline case. This result indicates that spatial density strongly impacts the diffusion of pathogens.

### Model evaluation results

**Fig 5:**
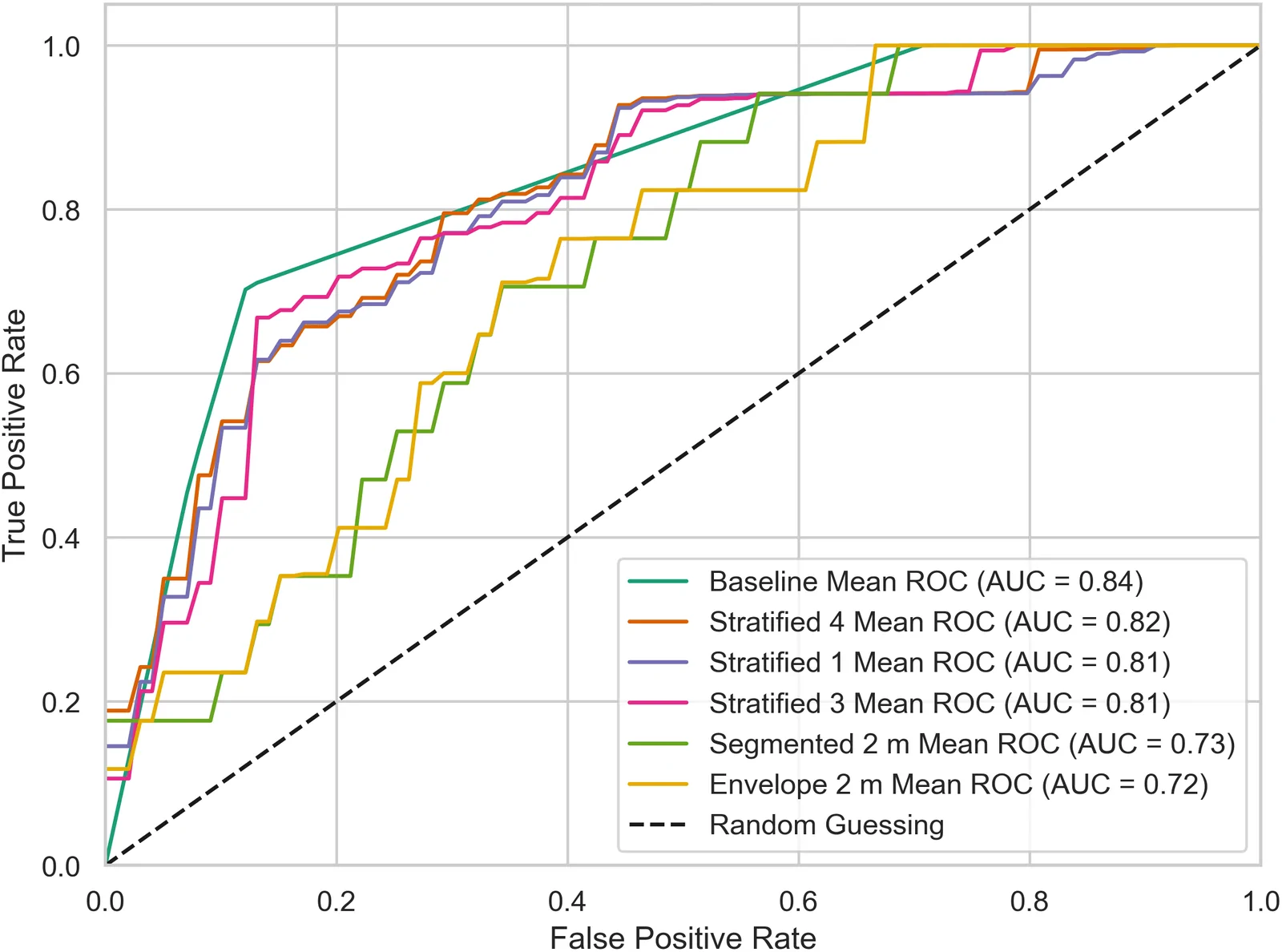
Mean ROC curves of best models. Colors correspond to the best models identified in each category and compared to the baseline case when no contact information is considered. The average ROC and AUC are estimated from the average over a parameter sample of 100 000 transmission probability values.

The ROC curve analysis was performed to assess the diagnostic performance of our models, with the mean AUC and its 95% HDI presented in Table 7. Fig 5 illustrates the average ROC curves for these models, showcasing their sensitivity and specificity across various thresholds. The analysis of AUC values provided critical insights into the predictive capabilities of the evaluated models. Notably, the baseline model and the Stratified 4 model exhibited the highest AUC values, indicating superior predictive accuracy, with the baseline model achieving an AUC of 0.84 and the Stratified 4 model closely following at 0.82. These findings suggest that both models are highly effective in distinguishing between different classes. Additionally, the Stratified 3 and Stratified 1 models demonstrated similar AUC values of 0.81 each, reflecting similar levels of predictive efficiency. In contrast, the segmented and envelope models yielded lower AUC values overall; however, within their respective categories, the segmented model and the envelope model performed relatively better at distances below 2 meters, achieving AUC values of 0.73 and 0.72, respectively. These results underscore the robustness of both basic and Stratified models in predictive tasks while highlighting potential limitations associated with segmented and envelope models.

**Table 7:** Comparison of AUCs for different models.

Model	Mean AUC	SD	HDI 95% lower	HDI 95% upper
Baseline	0.84	0	0.84	0.84
Segmented 1 m	0.67	0	0.67	0.67
Segmented 1.5 m	0.68	0	0.68	0.68
Segmented 2 m	0.73	0	0.73	0.73
Envelope 1 m	0.65	0	0.65	0.65
Envelope 1.5 m	0.68	0	0.68	0.68
Envelope 2 m	0.72	0	0.72	0.72
Stratified 1	0.81	0.02	0.76	0.84
Stratified 2	0.66	0.01	0.64	0.67
Stratified 3	0.81	0.01	0.78	0.83
Stratified 4	0.82	0.02	0.76	0.84
Stratified 5	0.71	0.02	0.68	0.73

In Table 8, we summarized the performance of the models based on the WAIC results. In the table “elpd_waic”: This is the expected log pointwise predictive density (ELPD). High values indicate better predictive accuracy. “p_waic”: This is the effective number of parameters in the model that provides an estimate of model complexity; “elpd_diff”: This is the difference in elpd_waic between the current model and the best model. Smaller differences indicate that the models have similar predictive performance; “se”: The standard error of ELPD estimate. “rank” indicates the ranking of models based on their WAIC values. A low rank indicates a better model.

**Table 8:** WAIC comparison of the different models to assess their performance and complexity.

Model	elpd_waic	p_waic	elpd_diff	se	dse	rank
Baseline	−27.93	0.88	0	2.45	0	1
Segmented 1 m	−35.55	1.07	7.62	5.52	4.49	10
Segmented 1.5 m	−35.28	1.12	6.99	5.41	4.5	8
Segmented 2 m	−31.52	1.04	3.59	3.75	2.61	5
Envelope 1 m	−35.57	1.27	7.64	6.41	4.99	11
Envelope 1.5 m	−35.28	1.12	7.36	5.61	4.68	9
Envelope 2 m	−32.11	1.07	4.18	4.05	2.97	6
Stratified 1	−28.95	1.25	1.02	3.39	1.9	4
Stratified 2	−36.31	1.32	8.39	5.58	4.53	12
Stratified 3	−28.77	1.15	0.84	3.29	1.76	2
Stratified 4	−28.88	1.30	0.95	3.32	1.86	3
Stratified 5	−32.29	1.30	4.37	4.02	2.87	7

We compared multiple models using WAIC. The baseline model had the highest ELPD (elpd_waic = –27.93) and thus the best out-of-sample predictive performance. The Stratified 3, Stratified 4, and Stratified 1 models showed minor decreases in ELPD relative to the baseline (elpd_diff = 0.84, 0.95, and 1.02 respectively), with standard errors that suggest these differences are not substantial (dse = 1.76, 1.86, and 1.9 respectively). All of these models had similar complexity, with p_waic values between 0.88 and 1.3.

The remaining models Segmented, Envelope and some Stratified models had notably lower ELPD indicating some problems in predictive performance. The elpd_diff values for these models were larger than the associated standard errors (se), supporting the conclusion that these models predict less well than the baseline and top Stratified models.

### Goodness of fit

**Fig 6:**
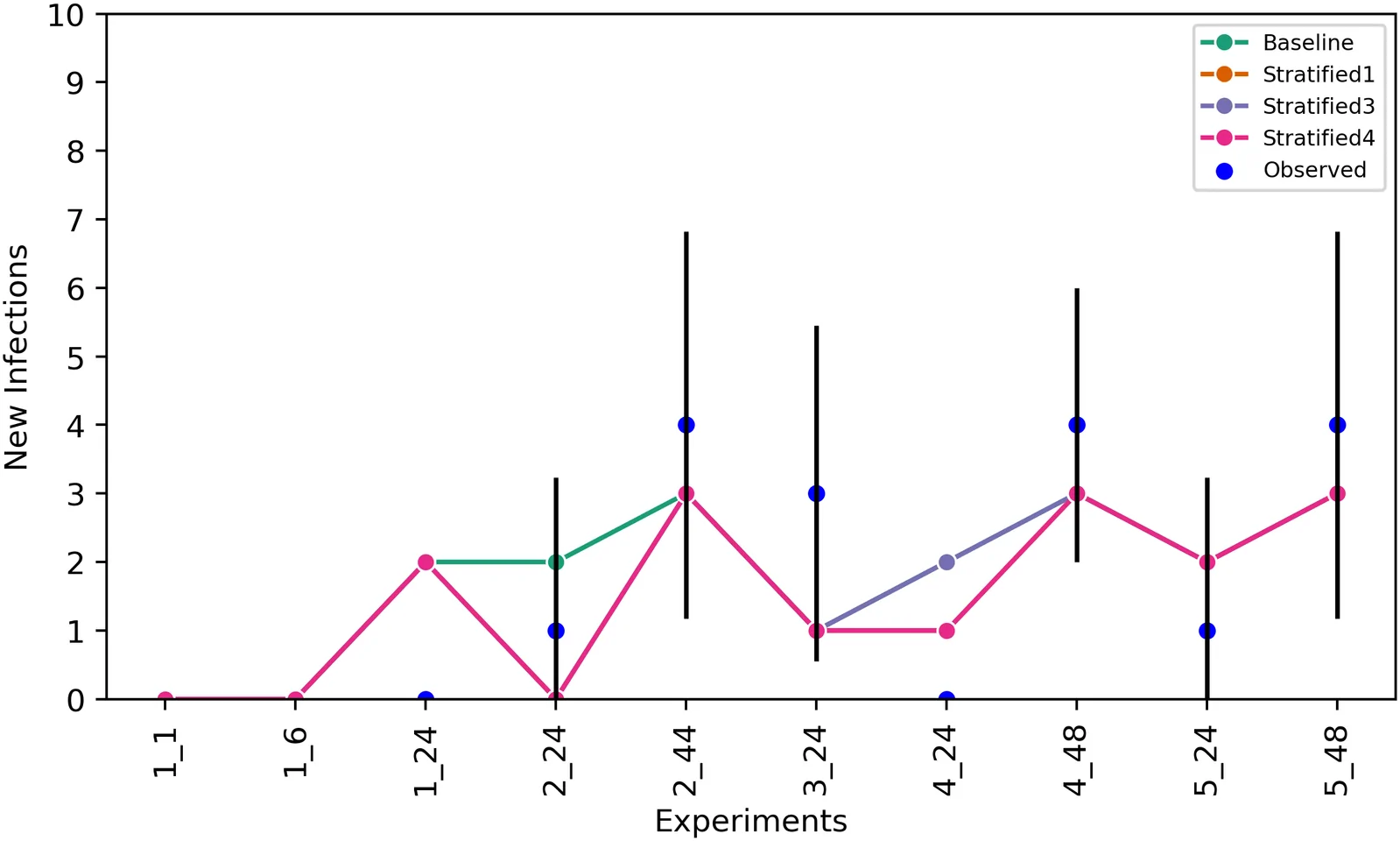
Comparison of model-predicted and experimentally observed PPR positive cases. Predictions are displayed as mode values, with error bars indicating the standard deviation, and real observations are presented as blue dots.

We conducted Bernoulli simulations using the estimated probability values p derived from the best-ranked models: Baseline, Stratified 4, Stratified 3, and Stratified 1, to generate binary outcomes (0 or 1) for each scenario. These simulations were grouped by experiments, and the total number of positive cases per experiment was calculated by summing the binary outcomes. The results are summarized in Fig 6, which compares the predicted mode number of positive cases against the real observed values. The results demonstrate that the four models are nearly juxtaposed, indicating that their performance in terms of fitting and prediction is almost identical. This suggests a high level of consistency across the models in capturing the underlying patterns of the data. Additionally, the predicted curves closely follow the trends of the real observations, further validating the models’ accuracy. However, an exception is noted in experiment 2_24, where the predicted outcomes deviate slightly from the observed trends.

### Exposure time estimation

**Fig 7:**
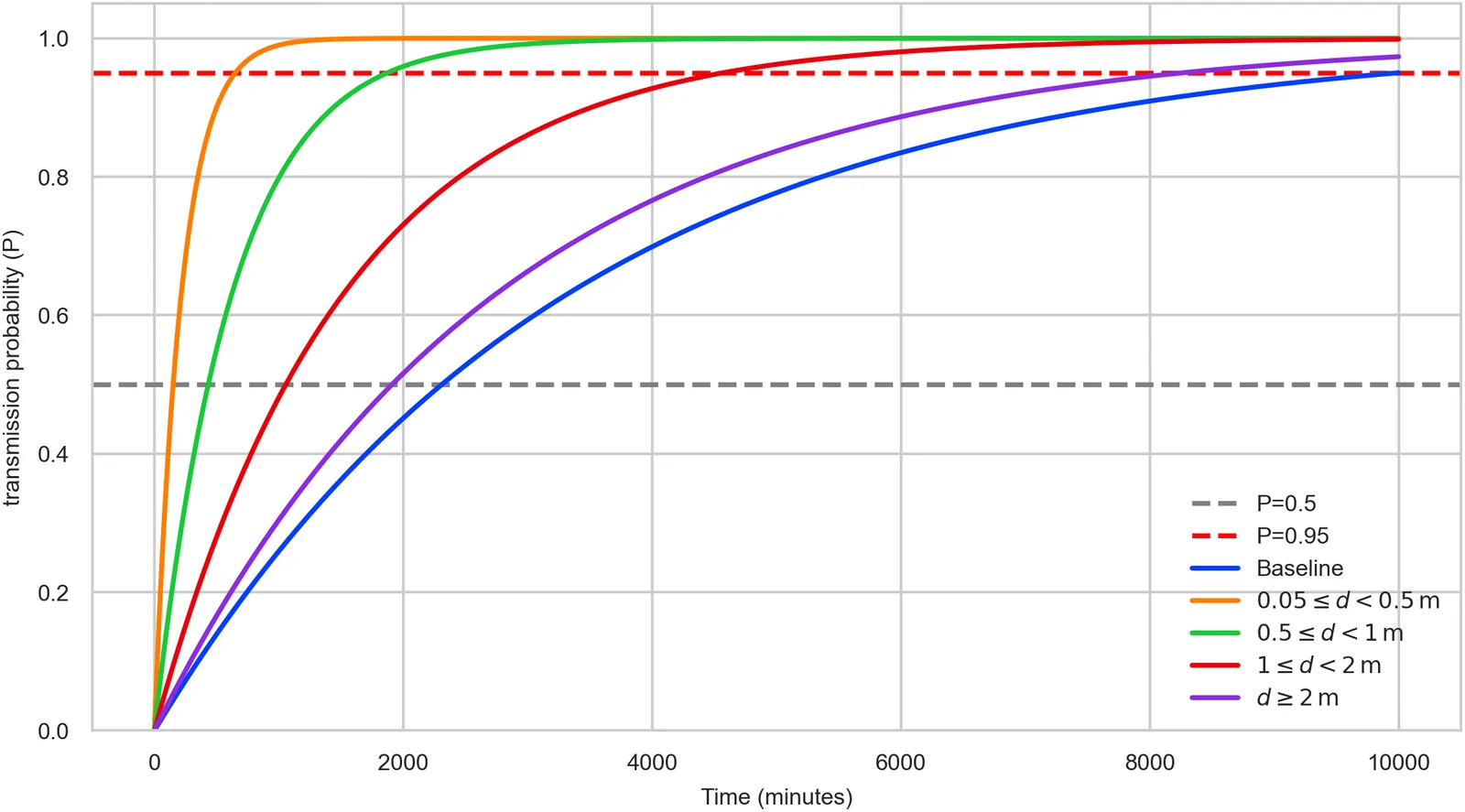
Estimating the minimal exposure duration required to achieve contamination. The solid lines represent the cumulative probability of an animal becoming infected after being exposed for t minutes. Colors indicate different scenarios: the baseline case without contact patterns, and hypothetical cases where animals remain at fixed distances, Stratified 1 was used for the first distance segment 0.05-0.5 m, and Stratified 4 for the remaining segments. Dashed lines mark the thresholds corresponding to 50% and 90% infection probability.

To estimate the minimum exposure time, we simulated various exposure scenarios. In Fig 7 we report the results of the estimation of the probability for an individual to get infected while in contact with an infected animal after a certain amount of time. We considered several scenarios, based on assumptions about contact patterns. We initially used the estimated values of the probability of success from the baseline to determine the minimum exposure time, measured in hours, needed for successful contamination. In this example, we assumed that animals could move freely and that the chance of infection is uniform. If one of them is infected, there is a 50% chance for a naïve animal to catch the infection after being exposed for about 38.50 hours corresponding to one day and a half. On the other hand, if the probability of infection is 0.95, it is estimated that the average exposure time is around 166.40 hours (almost one week). These estimations are based on the fact that animals can move freely and interact at short distances rarely. However, livestock markets are very crowded places, where thousands of animals spend 6–8 hours in very close contact. To estimate the exposure times in these conditions, we considered simulations using the parameters for close contacts estimated by our models.

The analysis of simulation using the Stratified 1 and Stratified 4 models revealed a consistent positive relationship between exposure time and the inter-individual distance. We used Stratified 1 model to estimate the exposure time related to a fixed distance within the first range of 0.05–0.5 m and Stratified 4 for the rest of distance segments. The exposure duration increased with distance to reach the probability thresholds of 0.5 and 0.95. To reach a transmission probability of 0.5, the mean exposure durations were 2.51 hours, 7.21 hours, 17.60 hours, and 31.83 hours for the distance segments 0.05–0.5 m, 0.5–1 m, 1–2 m, and ≥ 2 m, respectively. To reach a probability of 0.95, the required durations increased substantially, with corresponding values of 10.83 hours, 31.18 hours, 76.09 hours, and 137.58 hours, respectively, across the same distance intervals. These estimates indicate a substantial increase in the average exposure time required to achieve higher probabilities of infection, highlighting the variability and escalation of exposure duration necessary as the risk of infection increases.

## Discussion

This study shows that controlled PPRV infection experiments, combined with high-resolution UWB proximity data, can improve estimations of the transmission parameters and show how contact structure shapes infection risk. The main finding of our study is that exposure duration alone explained much of the observed infection pattern, but distance-resolved data were essential for translating that risk into biologically meaningful scenarios, especially when animals were densely grouped. This is relevant for pens, transport, and livestock markets, where short-range contacts may accumulate over several hours.

Our experimental results yielded an estimated daily transmission rate $\beta_{d}$ = 0.86 (95% HDI: 0.62–0.95). This corresponds to a derived $R_{\text{0}}$ estimate of 4.3 (95% HDI: 3.1–4.8) when an infectious period of 5 days is assumed, as in ElArbi et al. (2019), and 8.6 (95% HDI: 6.2–9.6) when an infectious period of 10 days is assumed, as reported by Fournié et al. (2018) [15], and Herzog et al. (2024) [17]. These two values serve as sensitivity estimates given that the infectious period was not directly measured. Our estimates are notably higher than some previous experimental studies, such as estimates vary with study type (modeling or experimental infection), geographical context (Western or Eastern Africa), husbandry practices (pastoralist against sedentary) as well as the value of the infectious period considered. In the context of experimental infection, we observed a difference when comparing our findings with those of Herzog et al. (2024) who reported a lower small-ruminant transmission rate under a different experimental design. Meanwhile modeling studies report values of approximately 1.61 in Mauritania [42], and between 0.15 and 0.62 in Ethiopia [15], depending on the husbandry practice. Similarly, the estimates for $R_{\text{0}}$ widely vary from around 1.5 in highlands Ethiopia (mostly sedentary agropastoral) [15], to 2.8 and 2.9 in Senegal and Mauritania [42,43], till 4 and 6 in Tanzania [44] and lowland Ethiopia. Besides the aforementioned factors, explanation for these differences can be traced back in experimental protocol.

Although PPR is a disease that affects all small ruminants, we used only goats in our reported experiments. To ensure the animals were fully susceptible, only PPRV seronegative ones, were placed in contact with a seeder. Following the exposure phase, they were kept strictly separated and isolated to confirm that any infection occurred only during the controlled contact time and solely from a single infected, virus-excreting animal. In the abovementioned studies, herds were mixed (either goat and sheep, either small and large ruminants) and animals of different ages were mixed. Because of this, animals with different susceptibilities to the disease were mixed thus reducing the number of effective contacts. Furthermore, in the abovementioned studies, parameters were estimated from final sizes and serology, while in our study the duration of the experiment was fixed a priori, and the focus was more on the number of animals that could be infected in a time window. In the study that was reported by Herzog et al. (2024), the animals were kept in common pens throughout the infectious period of the experiment, which enabled continuous contact between infected and susceptible animals, with possibilities of secondary infections in that case, and the statistical model was calibrated on data collected over several days. Because of this the number of new infections could decrease over time, and the transmission parameter could represent a sort of temporal average. In our study, instead, contact between infectious and naïve animals was allowed for a fixed period of time in view of avoiding secondary sources of infection.

Until now, the average incubation period of PPR is considered to range from 4 to 6 days, though it can range from 3 to 10 days. For surveillance and control purposes, the WOAH recommends adopting a conservative incubation period of 21 days [45]. Our estimated mean incubation period was about 14.7 days (95% CI: 11.6–20.1 days). This estimated value is longer than the commonly cited PPR incubation periods. We pooled first clinical signs, RT-PCR positivity, serology and post-mortem detection to estimate the incubation period. So, this could explain the difference between the previous estimates and ours. Still, the result highlights a surveillance concern: infected animals may remain without overt clinical signs for a relatively long period during which they will move between herds, markets, or different administrative areas and sometimes over long distances before detection of the disease. This extended virus infection latency, coupled with the often-asymptomatic nature of infected animals, presents a significant challenge to effective disease control. Those challenges are of particular importance in Africa, where animals are kept mobile for different reasons such as transhumance, a long incubation period could mean that asymptomatic animals could travel several hundred km, and cross several national borders, before disease could be detected. This poses a threat to the control of disease and demands stricter and more harmonized surveillance measures and vaccination activities among countries in the area, restriction/control in animal movements, implementation of animal containment quarantine.

Direct transmission requires close physical contact between animals. The concepts of “contact” and “effective contact” are fundamental yet complex ideas in mathematical epidemiology. For contact to be effective, animals must be close enough for droplets to travel from infected to susceptible individuals. However, throughout a typical day, animals move and interact at different times and for varying durations. As a result, the possibility of pathogen transmission is shaped by the pattern of effective contacts.

A major strength of our study is the innovative use of UWB-based indoor positioning to reconstruct high-frequency, metric inter-animal distances. This provides a significantly stronger basis for transmission modeling compared to conventional binary contact/non-contact definitions, representing an important methodological advancement. This high-resolution spatial data allowed us to explore how proximity affects pathogen transmission. Our analysis of segmented and stratified models reveals that specific distance intervals contribute differentially estimated infection risk. For instance, very close proximity (e.g., < 0.5m) likely represents direct contact transmission, involving physical touch or direct droplet transfer. Intermediate distances (e.g., 0.5-2m) could indicate short-range droplet/aerosol exposure, where infectious particles travel through air over limited distances. Longer distances (e.g., > 2m) might reflect shared airspace effects or indirect transmission through contaminated fomites within a communal environment. Explicitly identifying and interpreting these intervals in biologically credible terms enhances the mechanistic understanding of PPRV spread. Similarly, for the envelope model, the parameters$\;p_{\text{0}}$ and λ describe the baseline probability of transmission at near-zero distance and the rate at which this risk declines with increasing distance, respectively. A high $\;p_{\text{0}}$ signifies a strong risk upon close interaction, while a rapid decline in λ indicates that transmission is highly distance-dependent.

Previous studies using networks constructed from data collected using UWB, or similar, devices employed threshold models to identify effective contacts (when two individuals were within 1–1.5 meters of each other) (http://www.sociopatterns.org/) based on a literature review [31,33,46–49]. For PPR, there is limited, if not none, information about transmission distances that could inform our model. Therefore, we calibrated statistical models with multiple distance-based hypotheses to account for activity patterns and heterogeneity, and to evaluate how proximity affects pathogen transmission. Our results indicate that individuals spend a very short amount of time—just a few minutes—close to each other. Moreover, the analysis of the temporal contact network revealed no clear contact patterns, suggesting that animals tend to avoid close interactions. This behavioral tendency to maintain distance effectively created a protective “spatial buffer” within the enclosure. This behavior may be due to a lack of familiarity, the animals in our study were young and, coming from different herds, and were not previously acquainted. This could have influenced their social behavior and reduced their gregariousness, especially considering that young animals typically remain close to their mothers and siblings. Consequently, the force of infection was not a continuous low-level stream, but rather a series of rare, high-intensity events. This behavioral “spatial buffering” explains why no transmission events were recorded in experiments lasting fewer than 24 hours; the duration was simply insufficient for random movement to eventually force a high-risk close-contact event. However, our Stratified 2 model identifies these rare, brief windows of face-to-face proximity (< 0.5 m) as the primary drivers of the outbreak, carrying a per-minute transmission probability (p) 39 times higher than the facility-wide average. This behavioral-probabilistic link suggests that PPRV is driven by discrete, high-risk interactions during spontaneous fluctuations in proximity, a dynamic that would rapidly collapse in high-density settings such as livestock markets. We are currently analyzing the footage by cameras that were in the animal facility. The results, to be reported in a second manuscript, will give more information about the contacts between animals in the same premises.

Despite ongoing research, there is little information on the minimum exposure period required for successful contamination. To this end, it becomes important to estimate a probability of transmission for “effective contact”. In the absence of a definition of close contact for PPR transmission, several models have been developed that considered different definitions of effective distance and transmission probabilities that decrease with distances. Nevertheless, the baseline model, that did not incorporate any information about contact patterns, slightly outperformed all other models based on the WAIC and the AUC-ROC. The observation that the simplest model, which does not incorporate distance, provided the best fit suggests that certain aspects of our experimental design or initial assumptions about PPRV transmission dynamics may need to be reconsidered.

While this shows that distance may not be a significant factor in transmission, it would be premature to draw solid conclusions without first evaluating the potential limits of our study design. The results indicate that the distances between animals differ greatly, with numerous interactions taking place at distances greater than 2 meters. Furthermore, the frequent contacts occurring at these longer distances are linked to elements that reduce the precision of models using distance-related characteristics. We also noted that the uneven distribution of interaction distances, very few close-range interactions, hindered our capacity to identify impacts on virus transmission. Future studies should focus on better assessing the effective distance for contamination, either by implementing numerical simulation for the trajectory of droplets, or by considering experimental infections at predetermined fixed distances.

Our findings on minimal exposure time estimates provide actionable insights for outbreak modeling and control strategies. These results demonstrate that significantly less exposure time is required for transmission in scenarios with higher contact density. For example, the minimal exposure time can scale by a factor of four from a highly dense herd (distance < 0.5m, density = 5 animals per m^2^) to a herd that is almost 35 times less dense, or by a factor of 12 when animals move randomly in a 100 times larger space. This translates directly into practical scenarios: in lower-density free-grazing systems, animals might require longer periods of interaction for transmission to occur, whereas in higher-density pen or market settings, even brief encounters could pose a substantial risk. These findings strongly support the effectiveness of physical distancing measures and provide valuable insights into safety protocols in controlled environments.

Despite these advancements, our study has several limitations. The experimental design involved a small sample size and a single PPRV strain, and was conducted within a confined experimental setting. While this controlled environment allows precise measurement of inter-animal distances and transmission events, it may not fully reflect the complexities of field scenarios, where environmental factors, seasonal variations, and diverse husbandry practices can influence transmission dynamics. Moreover, the experimental contact conditions—characterized by restricted animal movement, a controlled environment, and a known infection source—do not fully represent naturally occurring contact patterns in pastoral systems, markets, or communal grazing, where animal mixing patterns and exposure routes are more complex and variable. Because our findings are limited to a single lineage IV PPRV strain (Senegal 20-GP) evaluated under controlled experimental conditions, they should be interpreted as indicating that the adequacy of the current 21-day quarantine period warrants further evaluation and validation under field conditions, rather than as a basis for revising current WOAH guidance. Further studies are needed to validate the derived estimate of the incubation period and to estimate the infectious period of additional PPRV strains, particularly those belonging to lineage IV, under field-relevant conditions. Future research should aim to validate these findings in larger, more diverse populations under varying field conditions and also with virus strains from other PPRV lineages.

Direct contacts between virus excreting animals and susceptible animals are certainly the main way, or the important way, for PPRV spread but it might not be the only one. The virus might spread through shared water or feed or through feces, as well as urine as suggested by some authors [50,51]. But those possibilities have yet to be proved experimentally. Combining UWB data with camera recordings could help identify contact hotspots and distinguish direct from indirect exposure. This would improve the biological interpretation of proximity data and support biosecurity measures that address both animal crowding and shared environmental resources.

## Conclusion

Ultra-wideband (UWB) captors proved effective for precisely monitoring goat movements and estimating infectious pathogen transmission probability and exposure time based on high-resolution proximity data. The transmission parameters derived in this study were obtained from experimental infections with a single lineage IV PPRV strain (Senegal 20-GP) under controlled contact conditions. These experimental contact conditions, which involved restricted animal movement and a known infection source, do not fully represent natural contact patterns in pastoral systems, markets, or communal grazing, where animal mixing patterns and exposure routes are more complex and variable. The presence of viral transmission in communal environments necessitates broad control measures addressing both immediate livestock surroundings and shared resources. Future research might investigate the following: conditions marketplaces where animals stay in close contact with important movements, possibilities of indirect transmission routes through feed and water, duration of virus viability in excretions. Additional research should focus on optimizing proximity data collection methodologies and quantifying close contact frequencies to enhance modeling accuracy. Studies incorporating larger herd sizes, and field conditions across diverse geographical regions would validate the applicability of these findings. Future research should focus on reducing uncertainty ranges and investigating additional variables affecting exposure times, our current results offer a robust foundation for developing evidence-based safety guidelines and risk management strategies. Additionally, investigation into environmental persistence of PPRV under varying climatic conditions would contribute valuable data for comprehensive control program development.

## Supporting information

S1 File Supplementary information. This document file contains additional analyses and extended methodological details supporting the findings of the study.
(PDF)

S2 File Official dataset. This Excel file contains follow-up data from experimentally infected goats, organized by individual experiment.
(XLSX)

S3 File Plos-one-humane-endpoints-checklist_PPR. (DOCX)
