# Supplementary material for: How fast can PPRV spread? Analyzing the results of an experimental PPRV infection of goats equipped with Ultra-Wideband sensors
Source: PLoS One. 2026 Sep 23;21(9):e0358839. doi: 10.1371/journal.pone.0358839 (PMC13600536; doi:10.1371/journal.pone.0358839)
Supplement: Supplementary file 1 [file pone.0358839.s001.pdf]

How fast can PPRV spread? Analyzing the results of an experimental PPRV infection of goats equipped with Ultra-Wideband sensors

Manal Nhili*, Jean-Baptiste Menassol, Gaye Laye Diop, Mariame Diop, Aminata Ndoye, Mbengué Ndiaye, Aminata Ba, Michel Dione, Assane Gueye Fall, Noha El Khattabi, Modou Moustapha Lo, Mounia Abik, Andrea Apolloni, Adama Diallo

Supplementary Information

Section 1: Experiment design

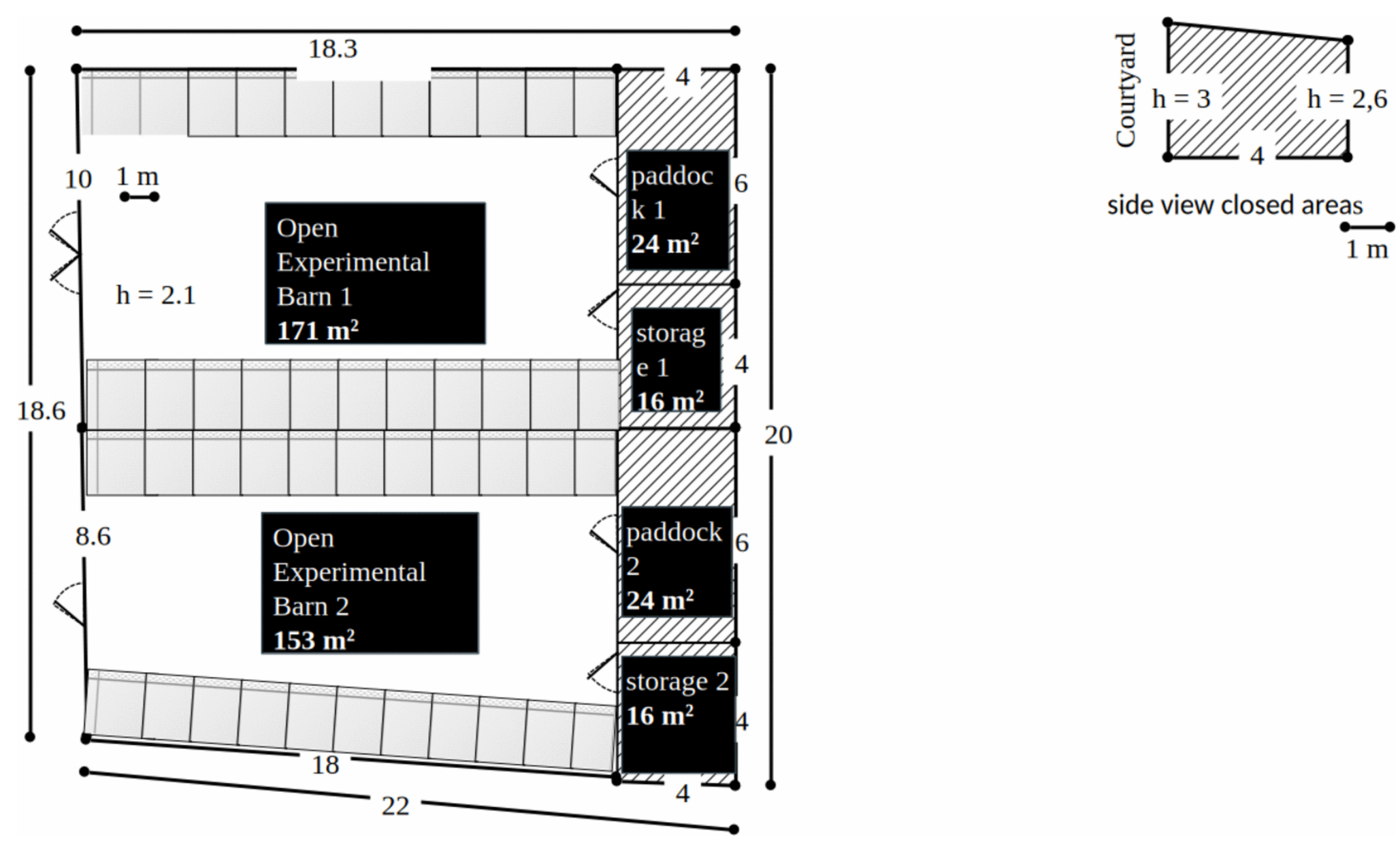

Fig S1. Schema of the Experiment Facilities. This diagram shows the experiment facilities maps including dimensions.

Experiment infection phase

In the preliminary part of the experiment, two goats were retrieved from the quarantine barn and moved to paddock 2 where from each animal ocular and nasal swabs were collected. Those collected samples will be used as "day 0" samples in different subsequent tests: antigen, nucleic acid, and serum detection tests. Just after this procedure, the two animals were subcutaneously inoculated with 10^3 TCID50 of PPRV Senegal 20-GP in 2 ml. The subjects were then housed in paddock 2 and subjected to daily clinical observation for monitoring any clinical signs of PPR expression, including rectal temperatures. All observations were recorded on a dedicated clinical sheet. The ocular and nasal swabs were also collected daily and submitted to tests for PPRV antigen detection and the nucleic acid detection by real-time RT-PCR following the method that was described by Batten et al. (2011) [1]. Following preliminary results of that test on samples 10 fold serial dilutions of the used PPRV, RNA extracted from the virus suspension at $10^{3.2}$ TCID50/ml gave the threshold cycle number (CT value) of 22.13 while that result was 27.0 for the $10^{0.2}$ TCID50/ml. Based on those standard samples results, once an inoculated animal expressed clinical signs of PPR and a good level of PPRV excretion in the collected swabs, CT value of the RT-PCR result less than 26, it was transferred from paddock 2 to paddock 1 to serve as a seeder for the exposure phase. Usually, this period of experimental infections lasted between six to ten days post infection.

Exposure phase

Once the seed animal was identified, five/six animals from the quarantine barn were randomly selected for the exposure period and housed in paddock 1. Each of the selected animals was bled for the day 0 serum collection. This bleeding was carried out along with the ocular and nasal swabs (in duplicate) collection. All those samples will serve as day 0 samples in the tests of other subsequently collected samples testing. Each animal was equipped with a collar-mounted UWB sensor (BeSpoon - STMicroelectronics, France) to triangulate their relative positions and estimate distances all along the experiment. At the time of the introduction of the seed animal into paddock 1, recordings were initiated. The length of exposure of the naïve animals to the seeder varied, with each exposure duration representing a distinct experiment.

Monitoring phase

At the end of the exposure phase, each animal was retrieved, its UWB collar was removed, and it was placed in an individual fenced pen in either open experimental barn 1 or 2. This move marked the start of the monitoring period that lasted for about 5 weeks (except for experiment 4 – 48 hour exposure – for which the duration was 8 weeks). The health status of each animal was monitored daily through visual observations without touching them. Once a week the animals were handled for nasal/ocular swabs collections and bleeding for serum. It was only on that occasion that the animals were touched and only by one animal attendant and the scientist in charge of the trial. For that, they wore personal protective equipment (PPE) which were changed between each animal. Outside of these sampling periods, only the animal attendant was allowed to enter the pens for cleaning and for providing feed and water in dedicated troughs, without making direct contact with the animals.

At any time during the monitoring phase, once an animal looked sick, its rectal temperature was recorded, nasal and ocular swabs were collected along with blood for serum. In such cases, only apparent sick animals were handled. In the event of death, a post-mortem autopsy of the animal was performed with the collection of some pathological samples: lymph nodes and piece of lung to be tested for the PPRV nucleic acid detection by real-time RT-PCR. The findings were recorded.

Following the exposure period, the seeder animal was returned to paddock 2. Daily clinical observations of the two PPRV-inoculated animals were conducted; to minimize the risk of cross-contamination, the in-contact animals were examined first. Because severe diarrhea is a frequent precursor to mortality in PPRV-infected animals, the onset of this clinical sign was established as the humane endpoint for euthanasia. Euthanasia was performed via intravenous injection of 25 mL of pentobarbital sodium (PENTOBARSOL™), a method that induces rapid and painless death, thereby minimizing distress. For biosecurity purposes, at the conclusion of each experiment, all remaining animals were humanely euthanized using this same protocol, and the facilities were thoroughly cleaned and disinfected.

Section 2: Distances distribution along the exposure phase

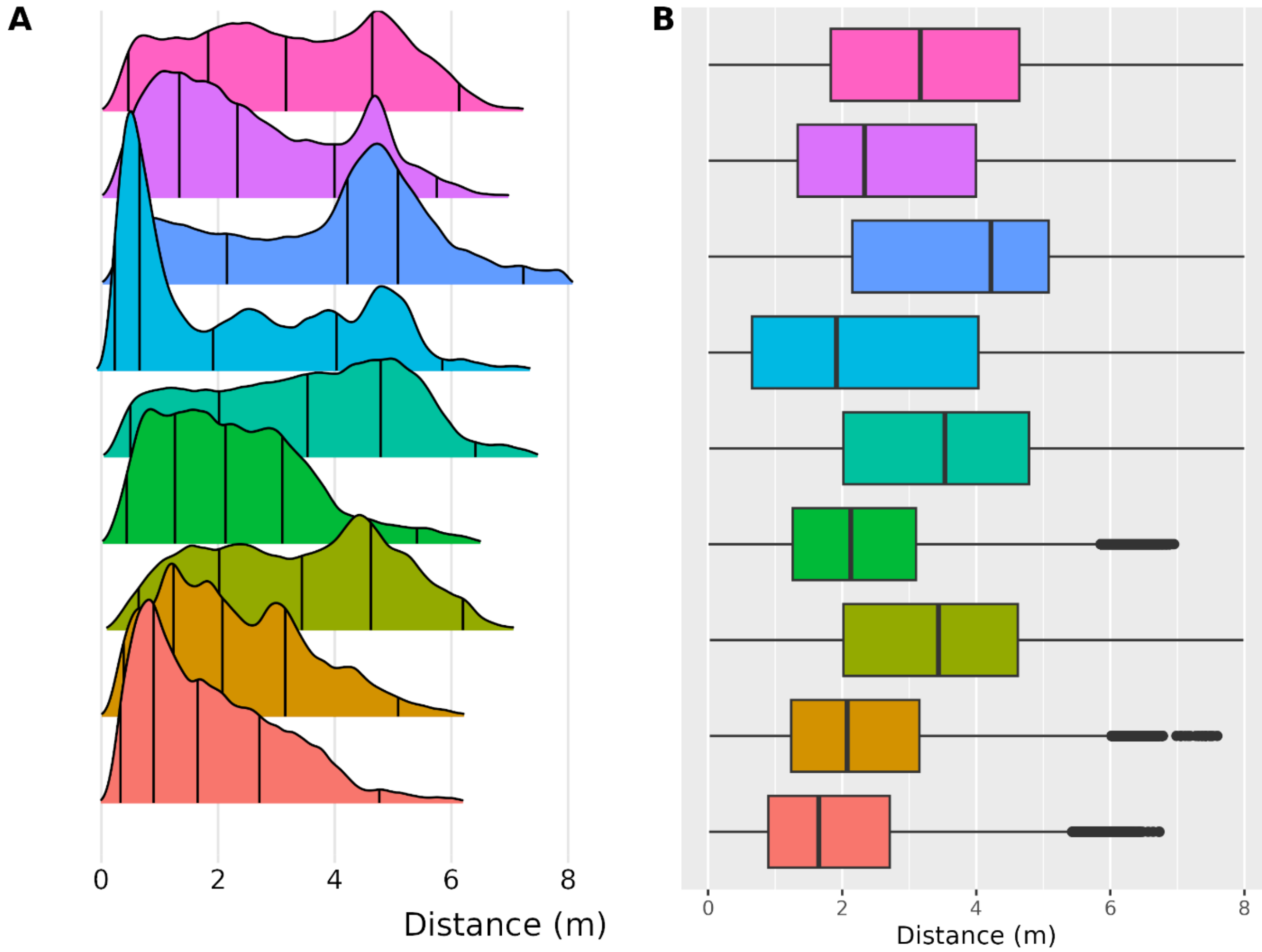

Fig S2. (A) Distribution of distances among animals per experiment. Colors correspond to different experiments. Vertical lines indicate quartile values of the distribution. **(B) Boxplot to resume the distribution.**

Fig S2 shows that there are no specific patterns of interactions that appear in any of the experiments, indicating that animals move randomly in the paddock trying to avoid each other. In Fig S3, distance distribution among animals is shown, divided by the experiment lot (row) and the duration (column), while colors correspond to the specific experiment. At each second of the exposure phase the distance for each couple of animals is estimated. In the plot the shortest, longest and median distance among couples is shown for each second of the phase.

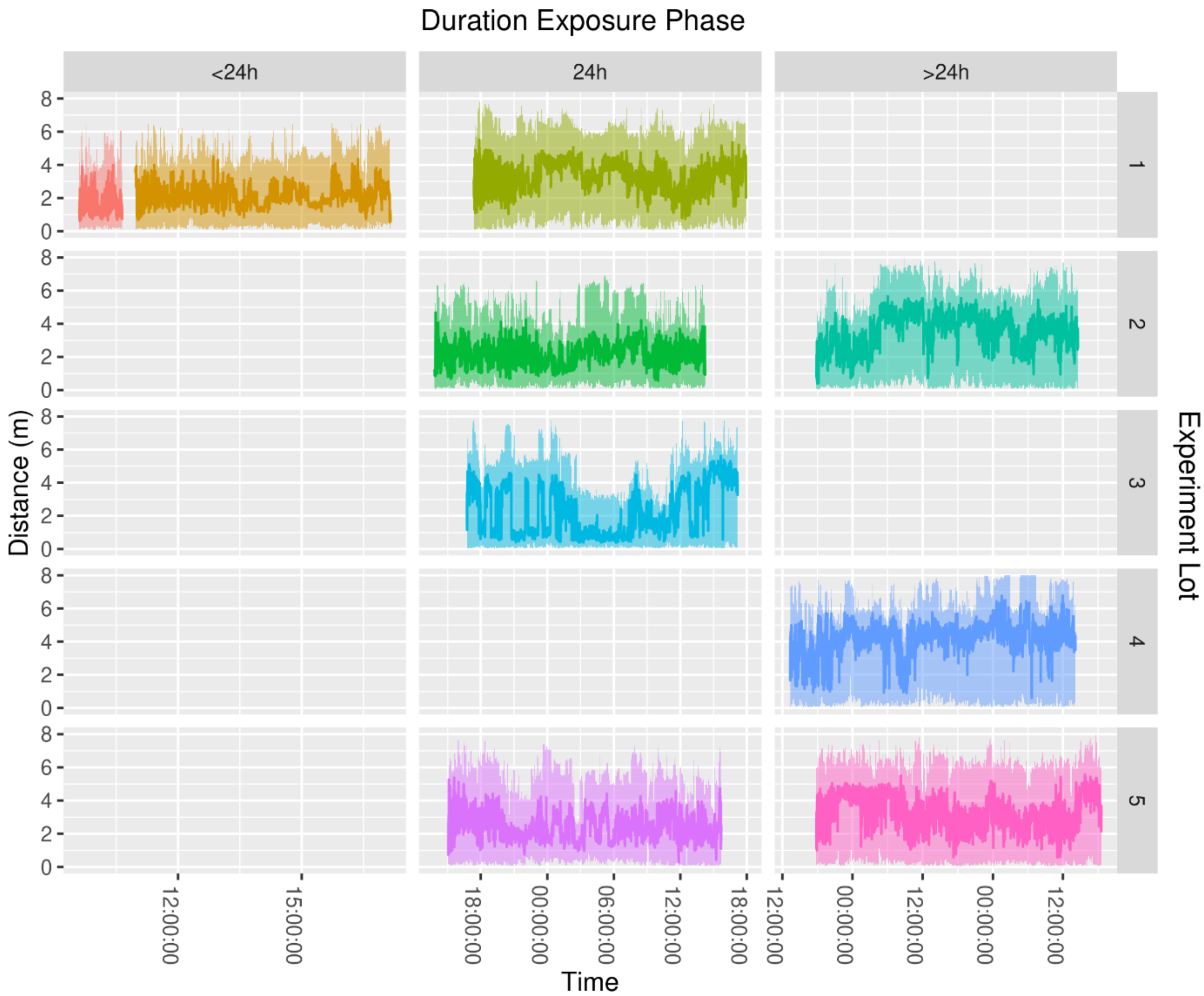

Fig S3. Contact patterns among animals. In each plot the median distance (solid line) and the minimal and maximal distance among animals is shown for each second of the exposure phase. Plots are organized by experiment lot (line) and duration (column) and color correspond to the specific experiment

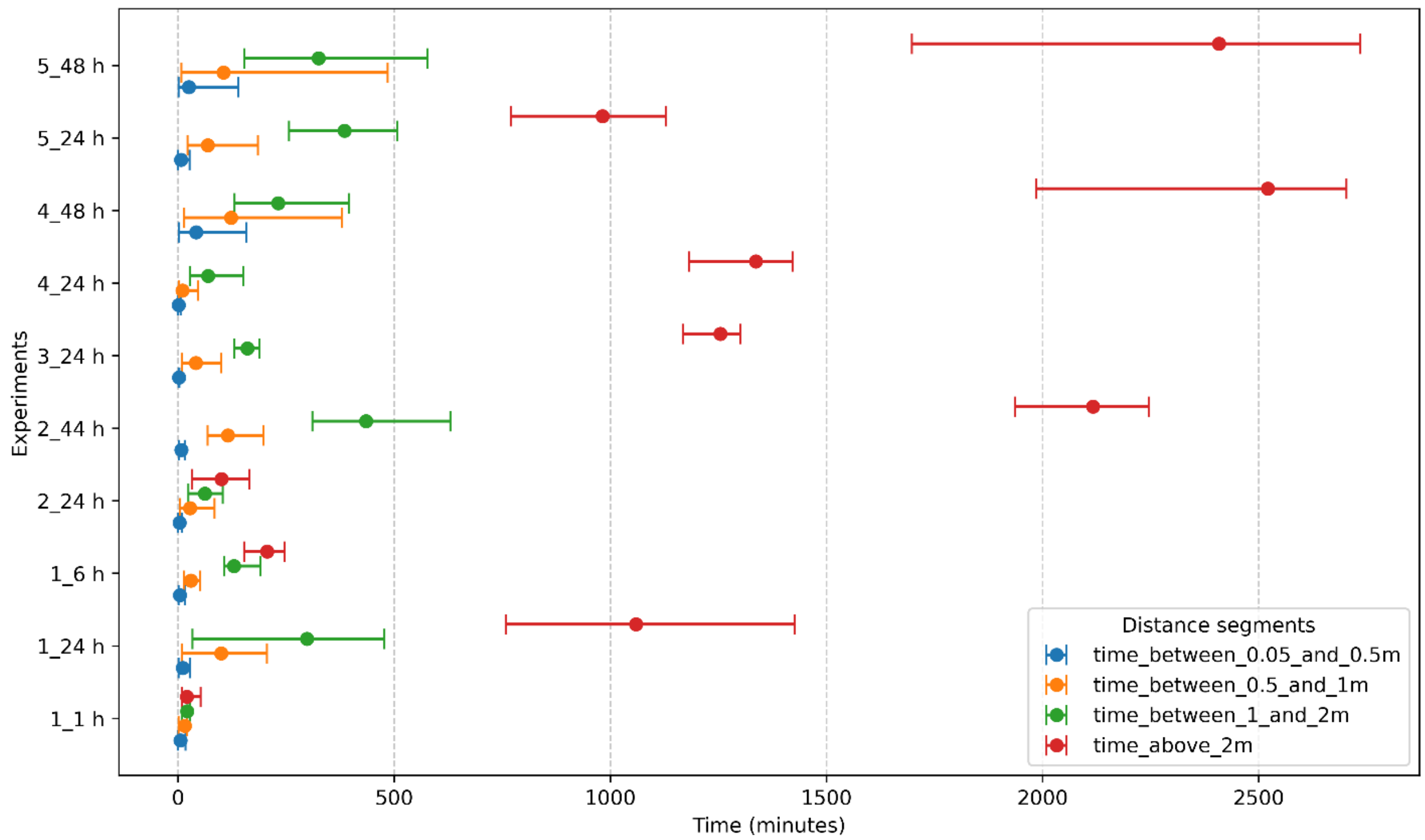

Fig S4. Contact time between seeders and susceptible animals' distribution under distance segments. For each experiment, the color of each bar corresponds to a specific range of distance, the dot corresponds to the average time a susceptible animal is at a certain distance from the seeder one, and the extreme indicates the minimum and maximum time.

Section 3: Description of the "envelope algorithm"

The "envelope algorithm" identifies clusters of stable distance between seeder and susceptible individuals (See Fig S5). This algorithm processes proximity data to extract "envelopes" - periods where the distance between individuals remains relatively constant (within a threshold ε) and observations occur at consecutive time points. These identified clusters serve as the fundamental units for the probability of transmission.

Our analysis uses a dataset of N observations, where each observation i is characterized by:

- d_i: distance between "seeder" and susceptible animal at observation i
- t_i: timestamp of observation i (in minutes)

We define an "envelope" as a cluster of observations that satisfies two conditions:

1. Temporal continuity: Adjacent observations occur at consecutive time points $t_{i+1} - t_i = 1$
2. Distance stability: The distance between consecutive observations varies within a predefined threshold ϵ, $|d_i - d_{i+1}| \leq \epsilon$.

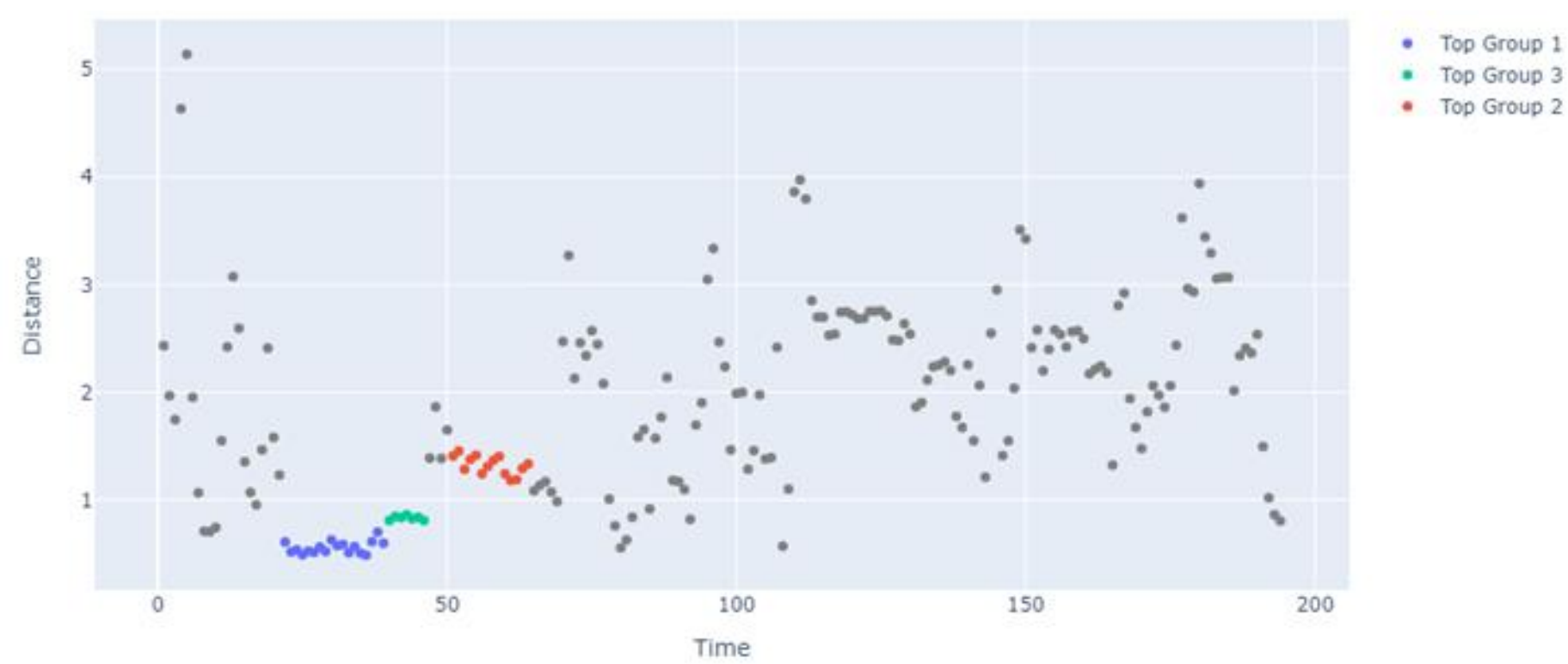

Fig S5. Envelope identification. An example of a snapshot of three envelopes identified for a pair "seeder-susceptible"

The "envelope model" is the probabilistic model that quantifies PPRV transmission probability using the envelopes identified by the algorithm. This model calculates transmission probability on a per-individual, per-minute basis by:

1. Taking the median distance of each envelope
2. Considering the cumulative time duration of each envelope
3. Applying a distance-decay function to calculate transmission probability.

$$P_i = 1 - \prod_j (1 - p(r))^{t_{i,j}(r)} , \; where \; p(r) = p_0 \cdot e^{-\lambda . r} \qquad (Eq. S1)$$

- r is the median distance of the enveloppe j for individual i.
- $t_{i,\,j}(r)$: cumulative time of envelope j for individual i
- p_0 : maximum probability when distance is null.
- λ : decay rate.

Section 4: Network analysis of the contact pattern

Data collected from UWB devices were used to reconstruct the contact patterns between animals. A temporal network approach was applied to represent and analyze these interactions. In this framework, animals are represented as nodes, and their interactions (based on proximity) as links with varying strengths (in this case, the distance). In a temporal network, the number of nodes, links, and their strengths (i.e., distance) can change over time.

In each experiment, the network consisted of 6 or 7 nodes—corresponding to the number of animals in the pen—and 15 or 21 possible links, respectively. While the number of nodes and links remained constant each time, the distances between animals varied. For each experiment, we analyzed the distribution of these distances. The Kruskal-Wallis test, followed by pairwise Wilcoxon tests, was used to assess whether significant differences existed among the distance distributions.

Additionally, we used the method developed by Berlingerio et al. [2] to identify possible patterns among animals. This approach relies on the use of a particular form of the Jaccard measure for weighted networks to quantify similarity over time:

$$J_w = \frac{\sum_{e \in E_t \cap E_{t+1}} \min\big(w_t(e), w_{t+1}(e)\big)}{\sum_{e \in E_t \cup E_{t+1}} \max\big(w_t(e), w_{t+1}(e)\big)} \qquad (Eq. S2)$$

where e indicates a link on the network $w_t(e)$ the corresponding weight, $E_{t,t+1}$ the set of edges at time t and $t+1$. After computing the Jaccard similarity between all pairs of consecutive time points, a hierarchical cluster approach is used to identify possible *eras,* period of times during which the dynamics among animals are different.

Section 5: Power analysis

The infection is a binomial process, where animals can get either infected (1) or not (0) with a certain probability p. To estimate the power of detection of the study, i.e.; the chance of having at least an animal infected among all the experiment for a specific value of the probability p, we followed the same procedure as in Herzog et al. (2024) [3]: we considered all the possible interactions between the seeders and the susceptible animals as trials (66) in a binomial process ; for a fixed value of the probability p , we simulated 1000 binomial extractions and estimated the

fraction of extractions where at least an animal get infected ; we repeated the same procedure for all the values of p between 0 and 0.1; we fixed an arbitrary threshold of 80% chance to have at least an infected animal and extract the corresponding probability of transmission. Supplementary Fig S6 shows the results of the procedure, where the red line indicates the threshold used for the detection probability while the vertical dashed line being the corresponding value of the transmission probability.

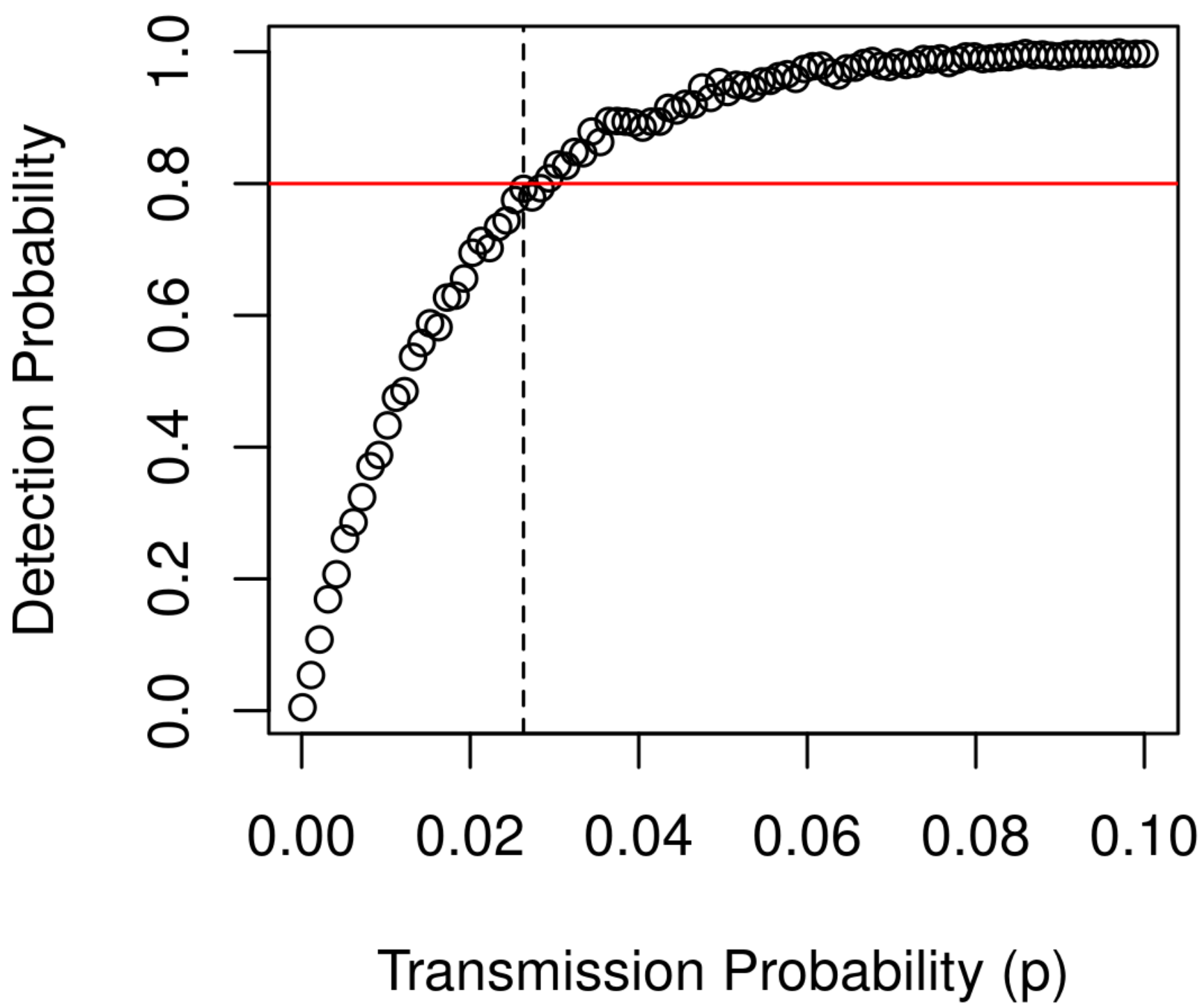

Fig S6. Power Analysis results. One thousand binomial extractions have been realized over all the possible animals involved (66) with different values of transmission probability p. For each value, the fraction of extraction with more than one case has been evaluated (dots). Solid red line indicates the threshold value for detection of at least a single animal, while the vertical intercept corresponds to the lowest probability the study can be detected in the study.

Section 6: Variations among experiments

Results from the null- logistic model, when only random effect is considered, show a significative value for the intercept (estimate = -1.4, $p < 0.01$). When the individual cumulative exposure and duration was included in the model, it showed a significant effect on infectious status (Type II Wald test, $p < 0.04$). Furthermore, the inclusion of exposure and duration substantially reduced the

between-experiment variance by more than 54% and 76%, respectively. However, the sensitivity analysis, which focused only on experiments lasting more than 24 hours, revealed that the effect is reduced.

Table S1. Results from the logistic regression of the infectious state against exposure and duration to check cluster effect and within correlation. In the sensitivity analysis models are restricted for the experiment of longer duration

Comparison Infectious Status	Model	Intercept (p-value)	Coefficient exposure (p-value)	Coefficient Duration (p-value)	Anova Chi-square (p-value)	Variance	Percentage Explained Variance
Original Model (All Experiments Included)	1 +(1|ID)	-1.4621 (0.0828)				3.968	
	exposure+(1|ID)	-1.296e+00 (0.179)	6.909e-01 (<2e-16)		1.2013 (0.27306)	1.811	54%
	Duration + exposure+(1|ID)	-1.841 (0.995)	-1.564e-05 (0.510)	Not significative	8.3518 (0. 01536)	0.9302	76%
Sensitivity Analysis (only long experiment)	1 +(1|ID)	-0.7356 (0.269)				2.309	
	exposure+(1|ID)	-7.130e-01 (<2e-16)	3.042e-01 (<2e-16)		0.2951 (0.58697)	1.7	26%
	Exp + exposure+(1|ID)	1.682e+00 (0.3394)	-1.564e-05 (0.5306)	-2.84e+00 (0.0493)	4.4035 (0.03586)	0.9302	61%

Section 7: List and characteristics of the tests used during the different phases

1°) Rapid test for PPR antigen detection in the field: ID.vet Innovative Diagnostic PPR pen-side test (ID Rapid® PPR Antigen) (IDvet: Montpellier, France)

2°) Antibodies anti PPRV detection test by competitive ELISA: ID.vet Innovative Diagnostic ID Screen® PPR Competition. (IDvet: Montpellier, France)

Section 8: Precision and accuracy analysis of the UWB devices used Sensors positions

There were nine different experiments corresponding to different geometrical structure of exchanges between sensors. These experiments are represented in the following figure (Fig S7):

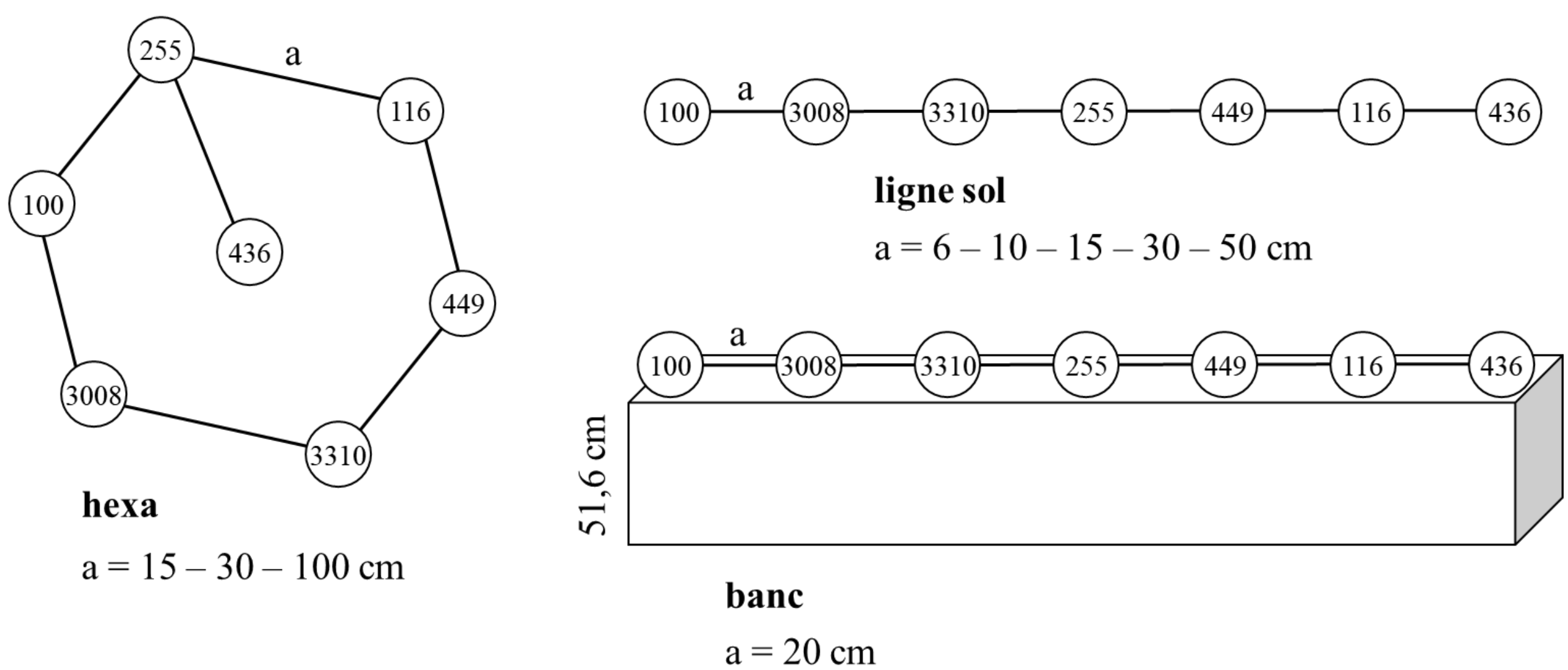

Fig S7. Sensors positions

Analysis of data

For each experiment, the position of each sensor is described as the mean +/- sd of their x, y and z values. These mean positions are stored in a list for each experiment and used to construct 3D plots of each sensor position.

Results

Tables of means +/- sd x, y, z positions for each sensor at each experiment

Table S2. Mean and standard deviation of 3D positions (x, y, z) per sensor from the validation test on *blanc_516_20*.

sensor	mean_x	sd_x	mean_y	sd_y	mean_z	sd_z
**100**	2.764704	0.0290412	1.145128	0.0349278	3.9024557	0.0489330
**116**	3.757055	0.0353763	1.287583	0.0286683	3.8295448	0.0442411
**255**	3.355336	0.0029041	1.295569	0.0482881	3.9185150	0.0426280
**436**	3.926876	0.0190089	1.129928	0.0244818	3.8768048	0.0277123
**449**	3.561531	0.0126671	1.212251	0.0388737	3.9522487	0.0576824
**3008**	2.940950	0.0178115	1.086868	0.0356895	3.9839767	0.0390408
**3310**	3.149653	0.0159596	1.564077	0.0233314	0.5497586	0.0229993

Table S3. Mean and standard deviation of 3D positions (x, y, z) per sensor from the validation test on *hexa_1000mm*.

sensor	mean_x	sd_x	mean_y	sd_y	mean_z	sd_z
**100**	2.812736	0.0108797	0.7624888	0.0522434	4.3764925	0.0639524
**116**	4.822824	0.0550450	0.9417785	0.0468361	4.2102541	0.0445432
**255**	3.853636	0.0698579	0.0874060	0.0507150	4.1047866	0.0744130
**436**	3.764684	0.0055016	1.3328056	0.0482116	4.5623855	0.0457083
**449**	4.713020	0.0443169	1.9486675	0.0612380	4.5356739	0.0587081
**3008**	2.822264	0.0406773	1.7111656	0.0427996	4.7490022	0.0471860
**3310**	3.843793	0.0285507	3.0116385	0.0325571	0.1517874	0.0560827

Table S4. Mean and standard deviation of 3D positions (x, y, z) per sensor from the validation test on *hexa_150mm*.

sensor	mean_x	sd_x	mean_y	sd_y	mean_z	sd_z
**100**	2.501545	0.0211449	0.8392764	0.0607993	4.4539436	0.0509052

**116**	2.901938	0.0031351	0.8276735	0.0187494	4.3186478	0.0415442
**255**	2.653749	0.0180602	0.6941909	0.0511404	4.4987304	0.0439493
**436**	2.808417	0.0068143	0.9505693	0.0212559	4.4432845	0.0769522
**449**	2.924737	0.0136025	1.0893366	0.0407295	4.5428563	0.0552602
**3008**	2.514746	0.0080637	0.8279182	0.0645808	4.4704557	0.0495762
**3310**	2.617181	0.0351648	1.5103589	0.0783469	-0.0121812	0.0481544

Table S5. Mean and standard deviation of 3D positions (x, y, z) per sensor from the validation test on *hexa_300mm*.

**sensor**	**mean_x**	**sd_x**	**mean_y**	**sd_y**	**mean_z**	**sd_z**
**100**	3.357897	0.0226948	1.2499751	0.0459102	4.5720369	0.0573869
**116**	3.903544	0.0101974	1.2794764	0.0340797	4.5086009	0.0356462
**255**	3.727984	0.0266732	0.9601817	0.0485282	4.5573522	0.0717773
**436**	3.539996	0.0512492	1.3616282	0.0787004	4.5839150	0.0579764
**449**	3.923349	0.0217106	1.3593598	0.0207331	4.6973414	0.0464197
**3008**	3.490395	0.0276789	1.7570831	0.0161918	4.6842694	0.0433082
**3310**	3.729201	0.0281826	2.3795158	0.0443889	0.0889439	0.0451942

Table S6. Mean and standard deviation of 3D positions (x, y, z) per sensor from the validation test on *ligne_sol_10*.

**sensor**	**mean_x**	**sd_x**	**mean_y**	**sd_y**	**mean_z**	**sd_z**
**100**	2.126689	0.0307549	0.9486915	0.0420417	4.3927023	0.0496740
**116**	2.668121	0.0155060	0.9815030	0.0428437	4.4776781	0.0575065
**255**	2.449434	0.0426000	0.9939865	0.0530741	4.5982951	0.0573403
**436**	2.794225	0.0220812	1.1649789	0.0459587	4.5888884	0.0476202
**449**	2.494602	0.0356559	1.0889936	0.0530280	4.5193883	0.0799993
**3008**	2.278398	0.0176812	0.7770745	0.0556226	4.5439384	0.0385095
**3310**	2.393823	0.0287350	1.5983624	0.0541210	-0.0880601	0.0424154

Table S7. Mean and standard deviation of 3D positions (x, y, z) per sensor from the validation test on *ligne_sol_15*.

sensor	mean_x	sd_x	mean_y	sd_y	mean_z	sd_z
**100**	1.595185	0.0462728	1.2604119	0.0620677	4.3533964	0.0328158
**116**	2.455342	0.0645793	1.0499303	0.0626752	4.4843659	0.0484096
**255**	2.131036	0.0557465	0.9975273	0.0817328	4.3992737	0.0530422
**436**	2.626329	0.0365414	1.0240452	0.0441171	4.5453852	0.0501229
**449**	2.346152	0.0386883	0.9910674	0.0637375	4.5557467	0.0444934
**3008**	1.792827	0.0570542	1.0626679	0.0606789	4.3483325	0.0513604
**3310**	2.016676	0.0279212	1.5971342	0.0271945	0.0494599	0.0216823

Table S8. Mean and standard deviation of 3D positions (x, y, z) per sensor from the validation test on *ligne_sol_30*.

sensor	mean_x	sd_x	mean_y	sd_y	mean_z	sd_z
**100**	1.635527	0.0312653	1.2732140	0.0540866	4.3842597	0.0321541
**116**	3.312649	0.0328660	0.8666348	0.0427861	4.6099050	0.0622555
**255**	2.627174	0.0103003	1.0612673	0.0133279	4.5540674	0.0593142
**436**	3.603676	0.0225731	0.9687466	0.0565814	4.6306697	0.0620424
**449**	2.932553	0.0311813	0.9385995	0.0439456	4.6469099	0.0356557
**3008**	1.931228	0.0465065	1.0672275	0.0703825	4.3580940	0.0402495
**3310**	2.337991	0.0327308	1.6641896	0.0471515	-0.0634381	0.0270201

Table S9. Mean and standard deviation of 3D positions (x, y, z) per sensor from the validation test on *ligne_sol_50*.

sensor	mean_x	sd_x	mean_y	sd_y	mean_z	sd_z
**100**	1.641318	0.0471806	1.2339220	0.0707023	4.3602634	0.0435603
**116**	4.409685	0.0397757	1.1527785	0.0536041	4.2825308	0.0153446
**255**	3.320725	0.0318042	0.8883387	0.0476338	4.6473161	0.0369833

**436**	5.078620	0.0497940	1.1109998	0.0513218	4.1215376	0.1058368
**449**	3.837337	0.0213973	0.9908899	0.0345242	4.5733078	0.0461560
**3008**	2.201015	0.0365706	0.8814425	0.0567458	4.4909232	0.0587161
**3310**	2.778897	0.0193464	1.6944885	0.0297285	-0.0323031	0.0380108

Table S10. Mean and standard deviation of 3D positions (x, y, z) per sensor from the validation test on *ligne_sol_6*.

**sensor**	**mean_x**	**sd_x**	**mean_y**	**sd_y**	**mean_z**	**sd_z**
**100**	3.605547	0.0324191	0.9741807	0.0523500	4.6276272	0.0976383
**116**	3.842073	0.0133120	1.0379823	0.0423026	4.5510214	0.0577873
**255**	3.728209	0.0095198	1.0282678	0.0310315	4.6018441	0.0436741
**436**	3.960541	0.0197692	1.0201435	0.0833597	4.5344053	0.0838957
**449**	3.813622	0.0336130	0.9505933	0.0850886	4.6385947	0.0995386
**3008**	3.681507	0.0375171	0.9294848	0.0573703	4.6604902	0.0620327
**3310**	3.694289	0.0304056	1.5430048	0.0148000	-0.1295655	0.0467304

3D graphs

As we can see on these graphs, sensor 3310 tends to be misplaced especially on the z-axis. Further data exploration should be undertaken for example to exclude data situated at the beginning and end of recording sessions or verify how sensor 3310 behave during further experimental procedures.

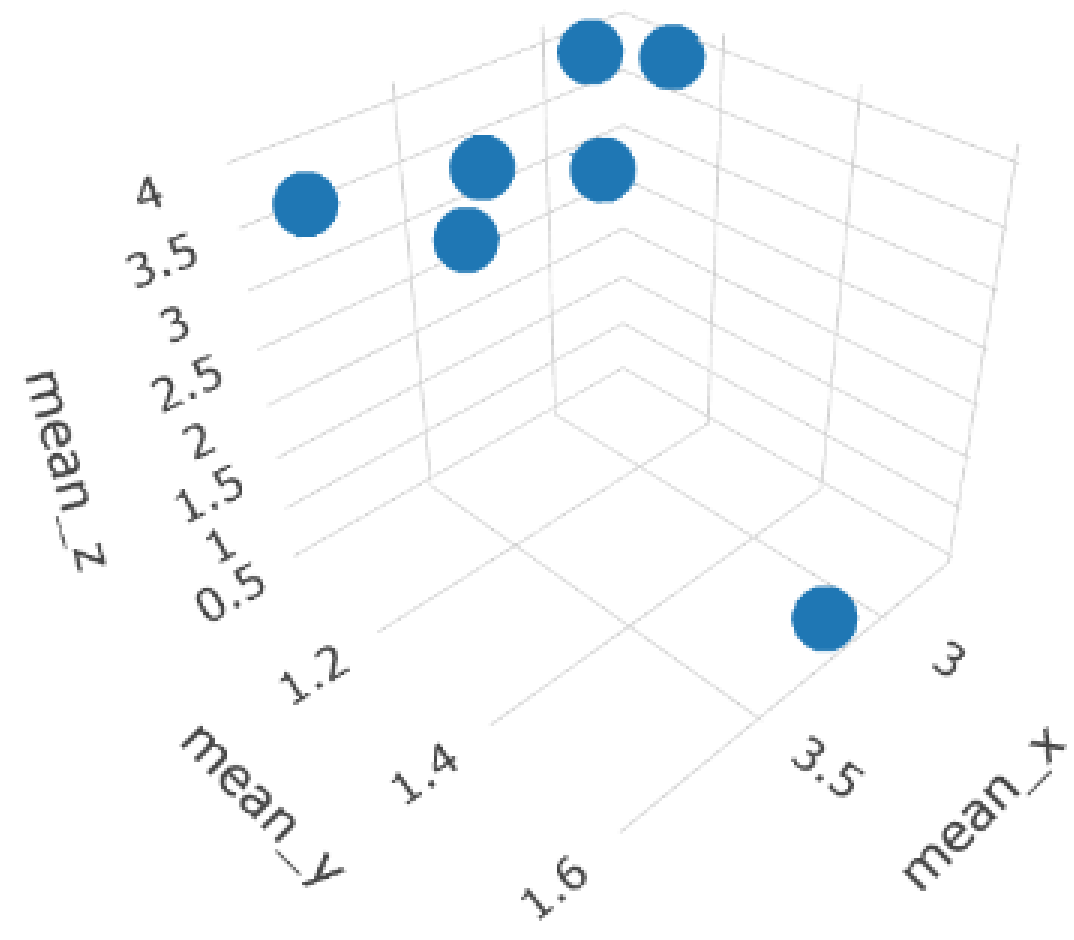

(a) *banc_516_20*

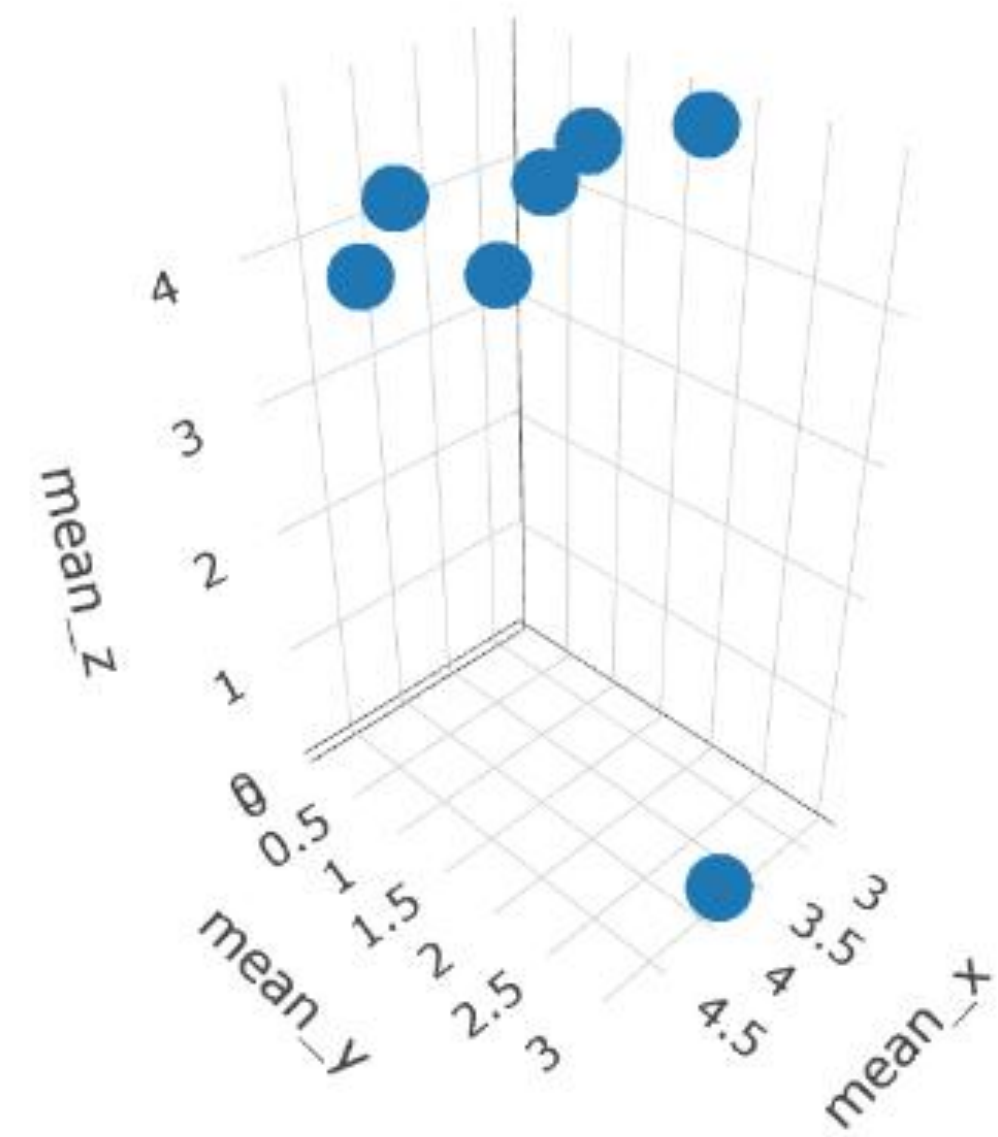

(b) *hexa_1000mm*

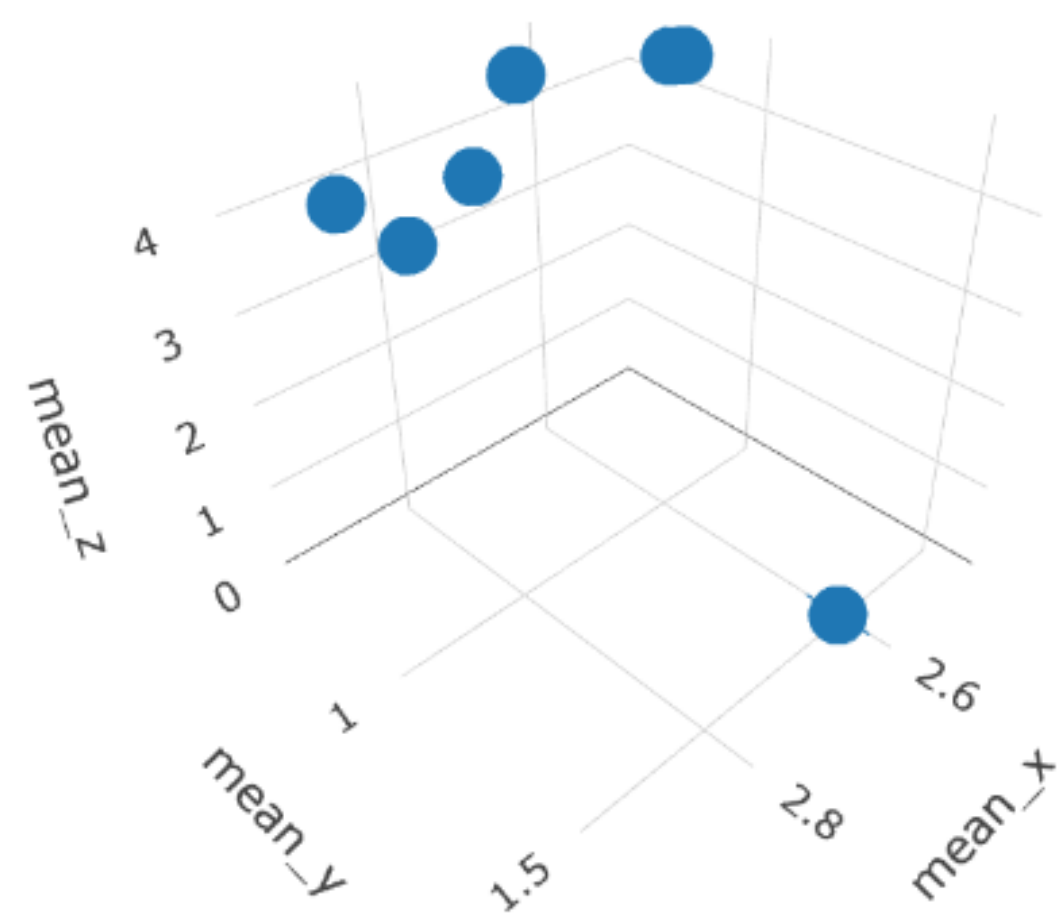

(c) *hexa_150mm*

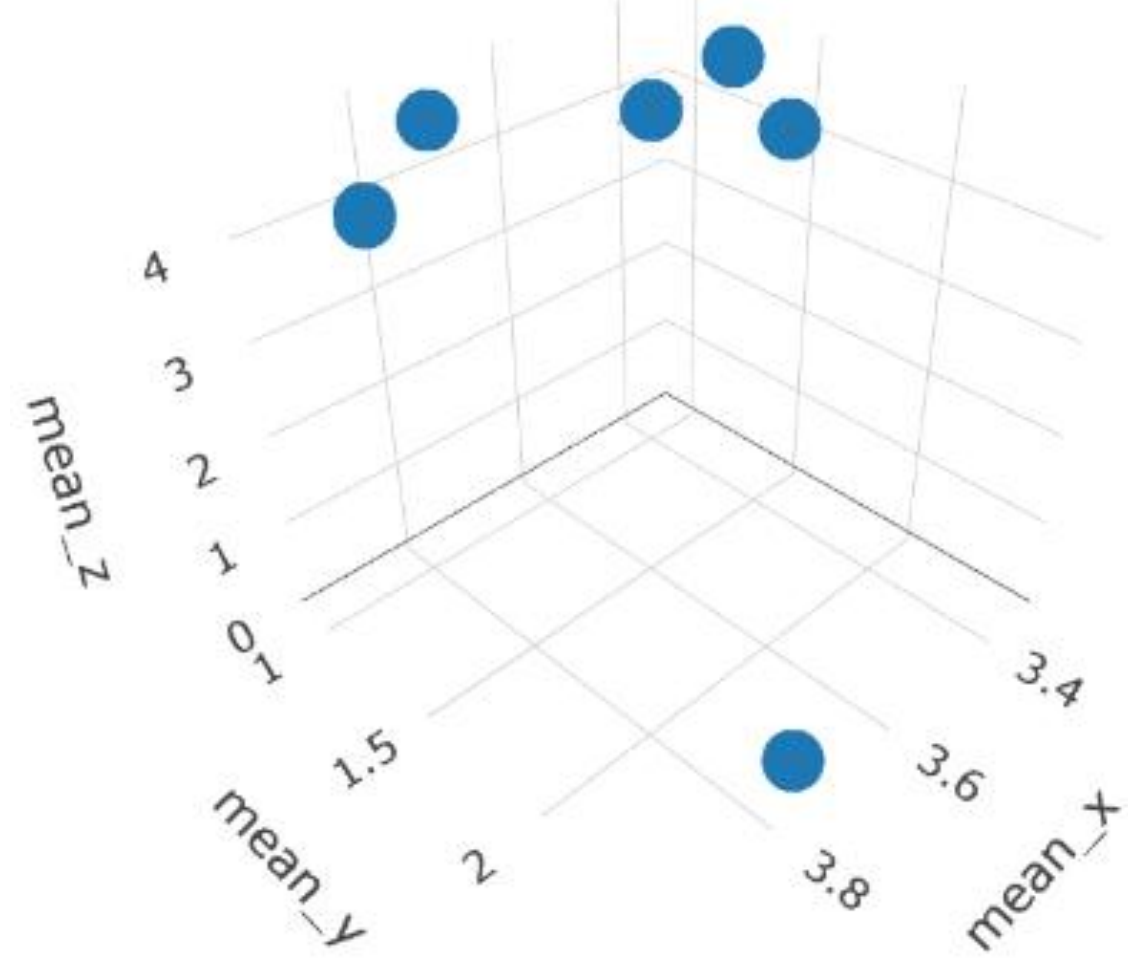

(d) *hexa_300mm*

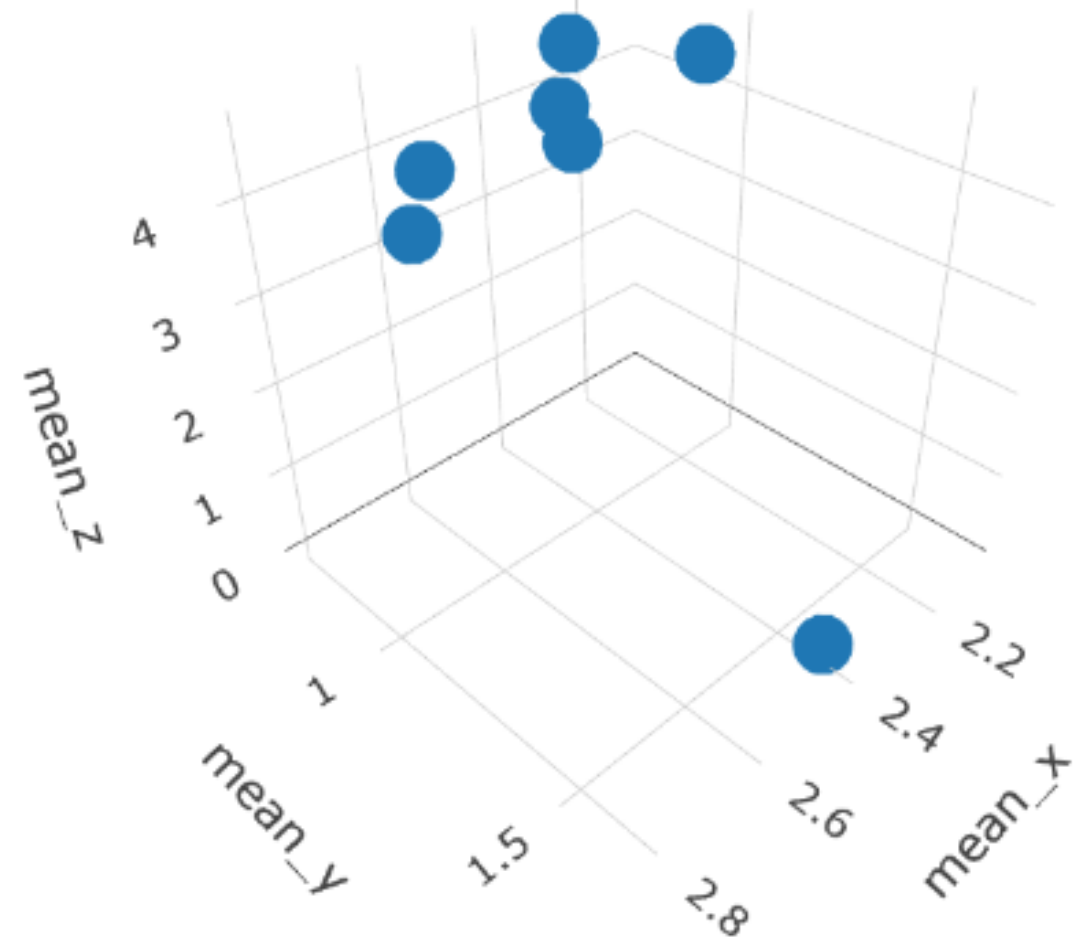

(e) *ligne_sol_10*

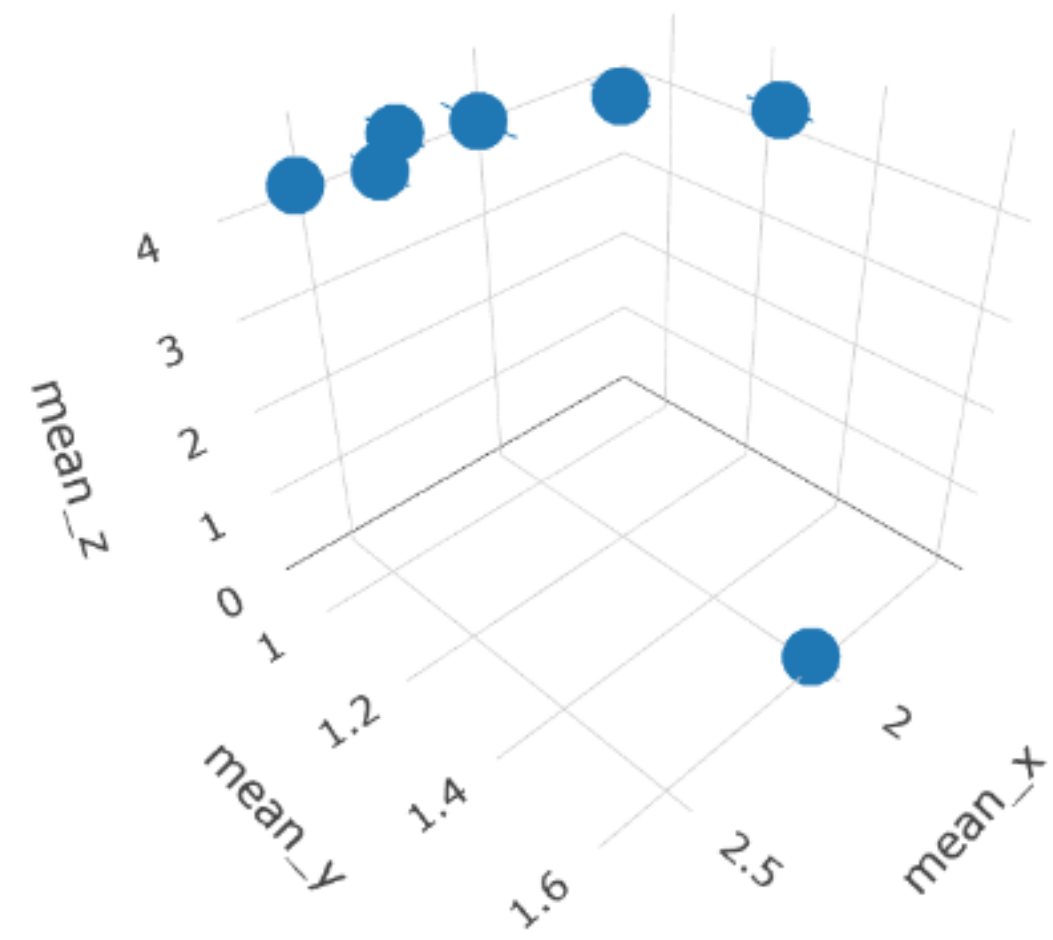

(f) *ligne_sol_15*

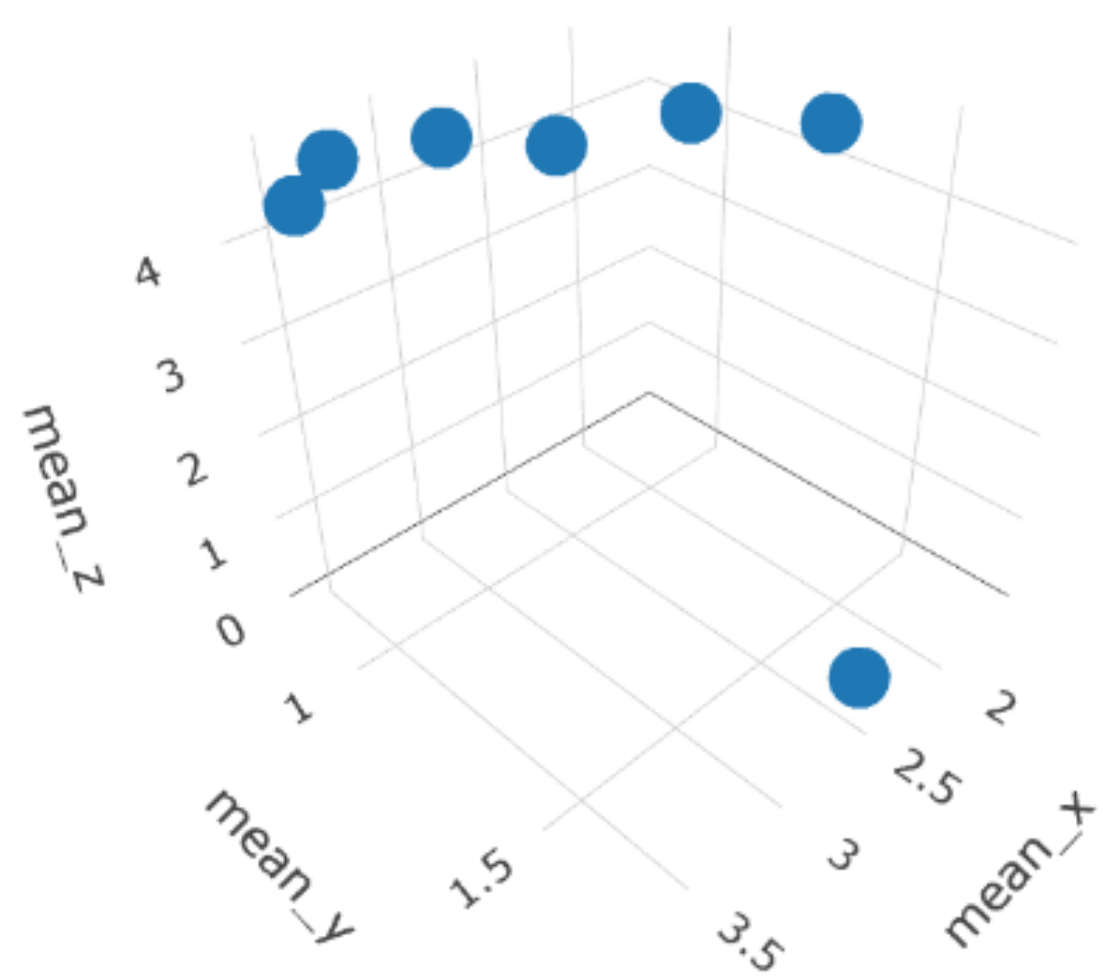

(g) *ligne_sol_30*

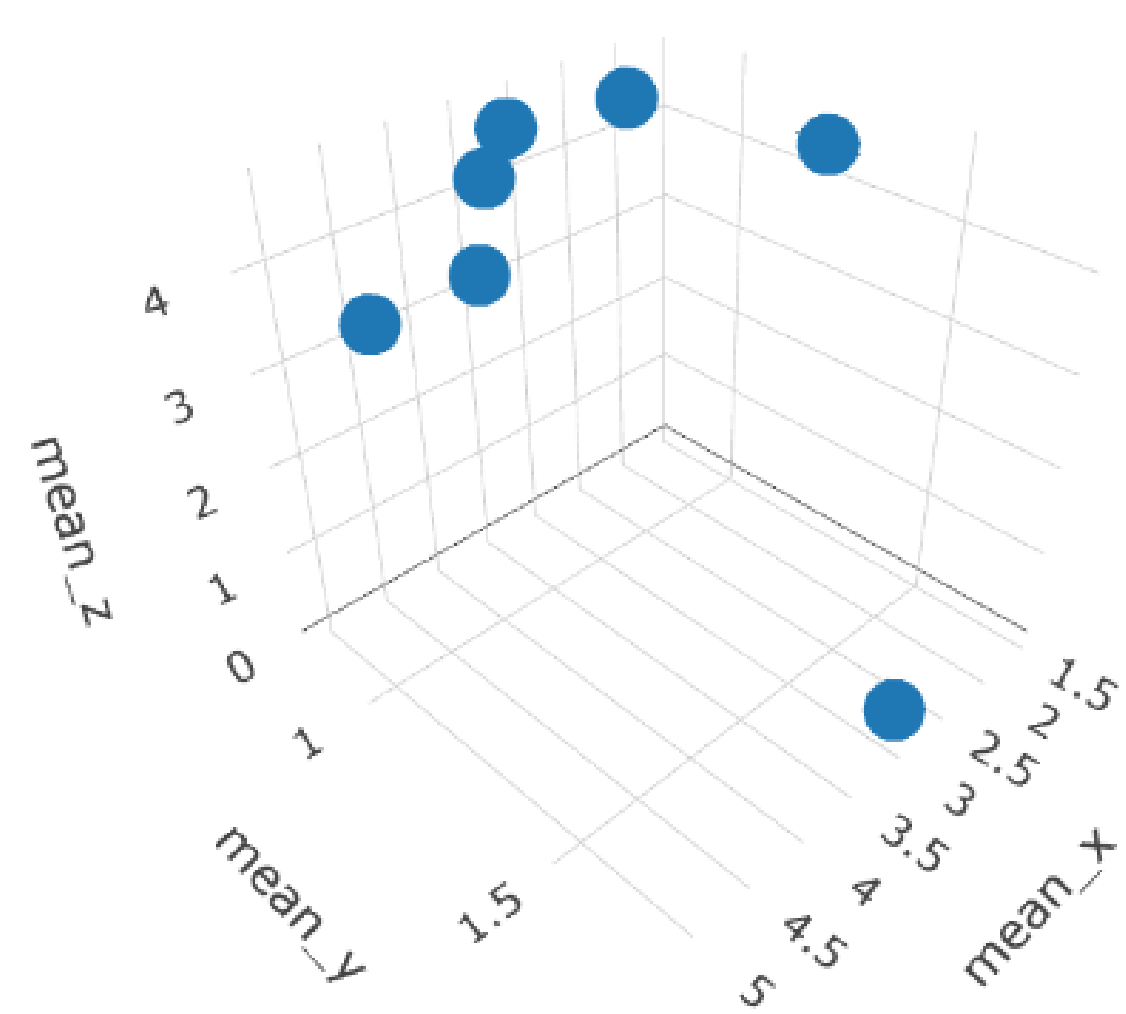

(h) *ligne_sol_50*

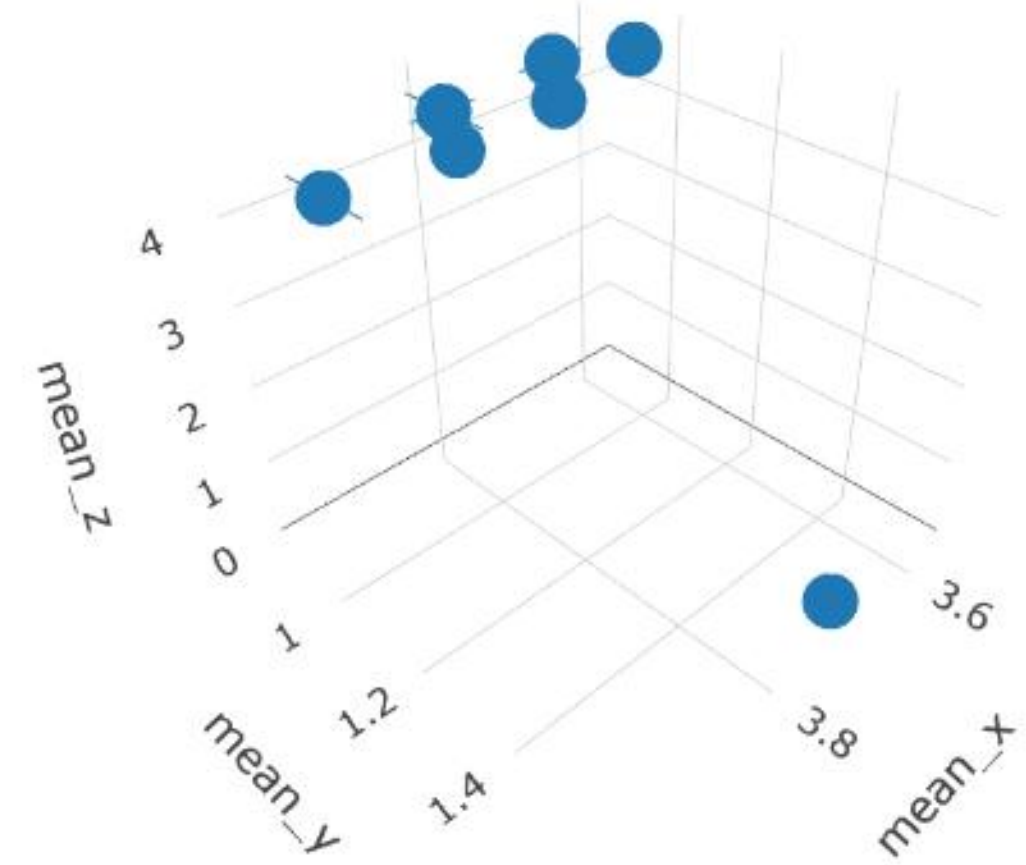

(a) *ligne_sol_6*

Fig S8. **3D visualizations of sensor positions from validation tests across various configurations.** Each subplot corresponds to a specific test case showing the spatial distribution of sensor positions.

Section 9: Parameters definition

Table S11. Comprehensive definitions, spatial assumptions, and biological meaning of parameters across all transmission models.

Model Class	Parameter	Mathematical Role	Definition & Spatial Assumption
**Baseline**	p	Constant probability	Probability of successful transmission per unit of time, assumed to be constant and independent of inter-individual distance.

**Segmented** (1 m, 1.5 m, 2 m)	p	Threshold probability	Probability of successful transmission per unit of time, **restricted exclusively** to contacts occurring within the specified distance threshold (e.g., < 1 m). Contacts beyond this threshold are assumed to carry zero transmission risk.
**Stratified** (Models 1 to 5)	p	Baseline probability	Baseline probability of successful transmission per unit of time for the **reference distance band (*j*).**
	α_i	Scaling factor	Scaling factor applied to the baseline probability (*p*) for the **non-reference distance band(s)** (i). The transmission probability in band i is defined as $\alpha_i \times p$
**Envelope** (1 m, 1.5 m, 2 m)	p_0	Maximum probability	Maximum probability of successful transmission per unit of time, defined specifically at a **distance of exactly 0 meters** (the intercept of the decay function).
	λ	Decay rate	Continuous spatial decay rate. It dictates how the probability of transmission decreases exponentially as the inter-individual distance increases from 0.

While the main text (Table 5/6) reports the posterior estimates, this table details the exact mathematical and spatial definition of each parameter to prevent erroneous cross-model comparisons. Note that in all models, the base probability parameter *(p)* represents the probability

of successful transmission per unit of time and *(P)* represents the cumulative probability per individual.

Section 10: UWB tracking system and field configuration

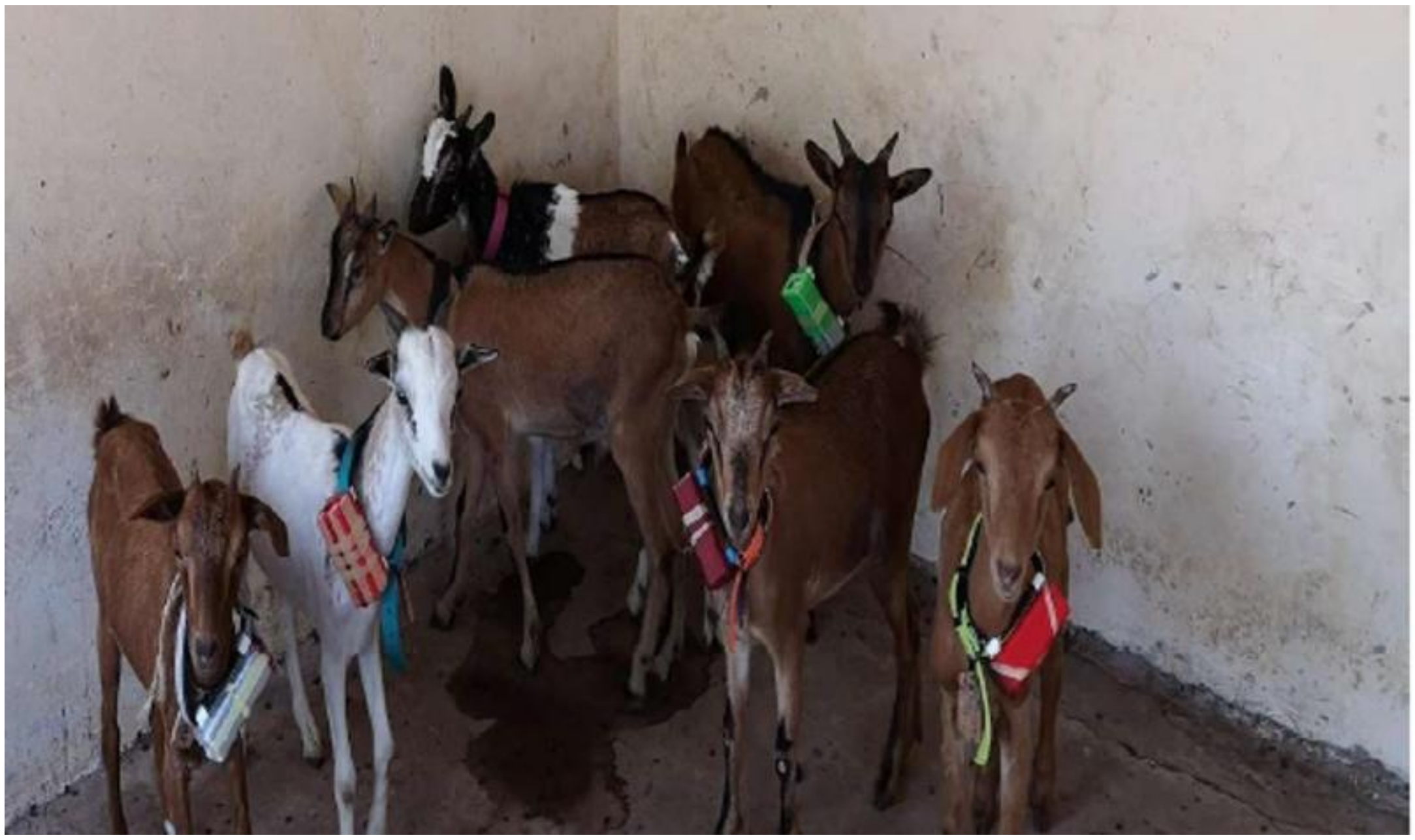

Figure S9: Goats equipped with the UWB sensors

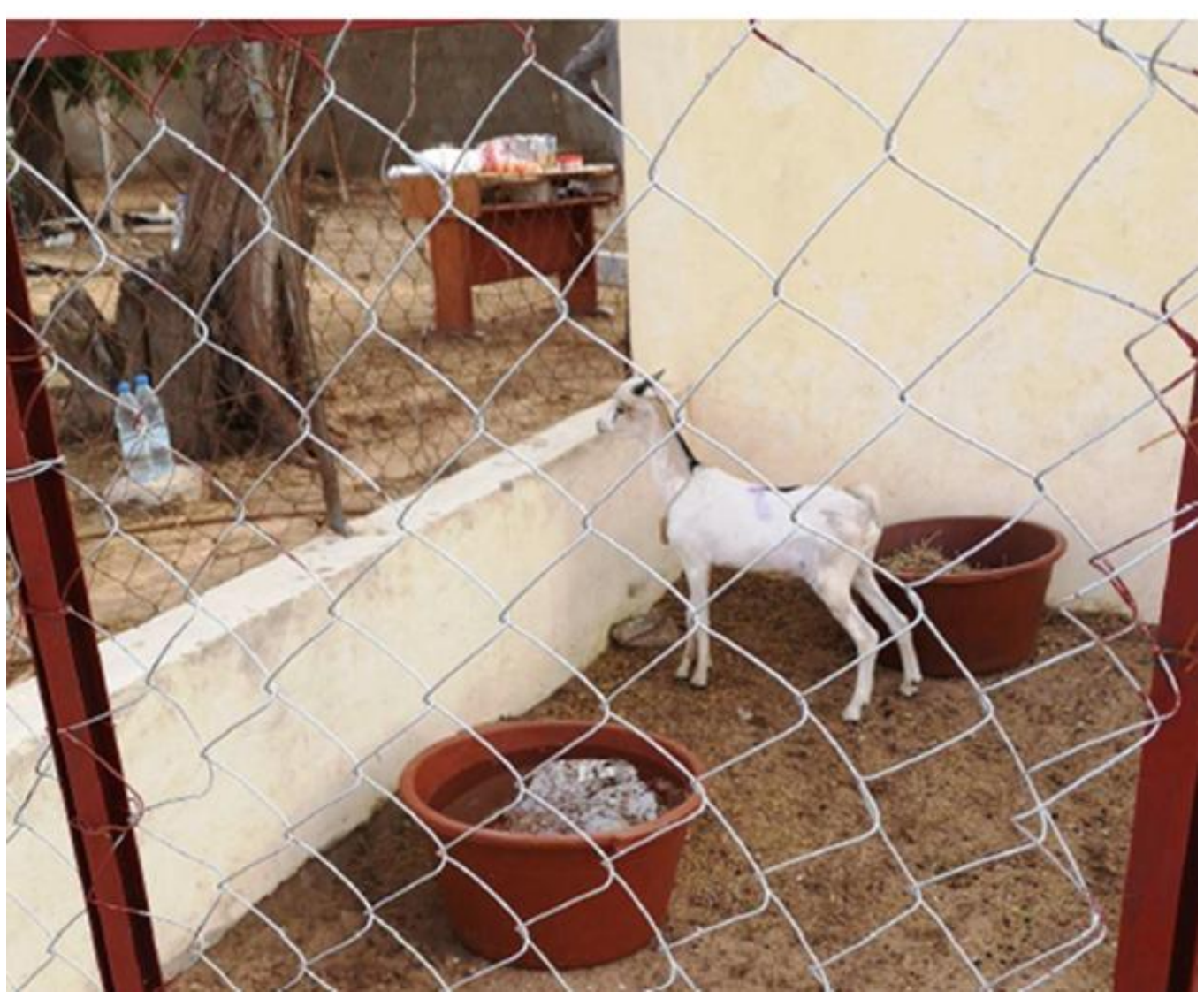

Figure S10: Isolated goat post-exposure

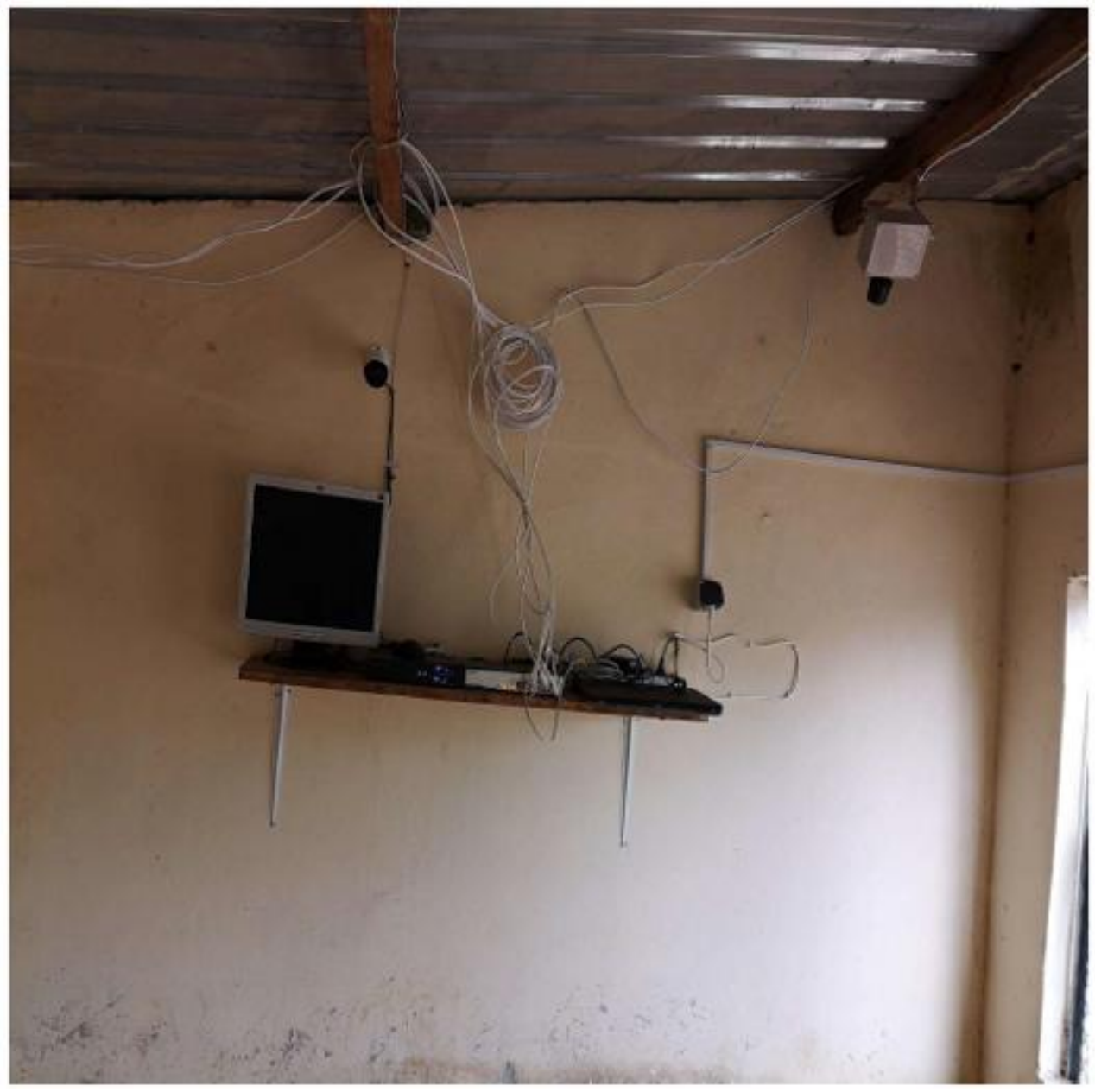

Figure S11: Server setup inside the paddock for real-time UWB position tracking

References

1. Batten CA, Banyard AC, King DP, Henstock MR, Edwards L, Sanders A, et al. A real time RT-PCR assay for the specific detection of Peste des petits ruminants virus. Journal of Virological Methods. 2011;171: 401–404. doi:10.1016/j.jviromet.2010.11.022

2. Berlingerio M, Coscia M, Giannotti F, Monreale A, Pedreschi D. Evolving networks: Eras and turning points. Pensa RG, Cordero F, Rouveirol C, Kanawati R, editors. IDA. 2013;17: 27–48. doi:10.3233/IDA-120566

3. Herzog CM, Aklilu F, Sibhatu D, Shegu D, Belaineh R, Mohammed AA, et al. Empirical and model-based evidence for a negligible role of cattle in peste des petits ruminants virus transmission and eradication. Commun Biol. 2024;7. doi:10.1038/s42003-024-06619-2
