# Supplementary material for: How fast can PPRV spread? Analyzing the results of an experimental PPRV infection of goats equipped with Ultra-Wideband sensors
Source: PLoS One. 2026 Sep 23;21(9):e0358839. doi: 10.1371/journal.pone.0358839 (PMC13600536; doi:10.1371/journal.pone.0358839)
Supplement: Supplementary file 2 [file pone.0358839.s008.pdf]

Authors’ Response

Manal Nhili*, Jean-Baptiste Menassol, Gaye Laye Diop, Mariame Diop, Aminata Ndoye, Mbengué Ndiaye, Aminata Ba, Michel Dione, Assane Gueye Fall, Noha El Khattabi, Modou Moustapha Lo, Mounia Abik, Andrea Apolloni, Adama Diallo

27 August 2026

Manuscript ID: PONE-D-26-21306R1

Title of submission: How fast can PPRV spread? Analyzing the results of an experimental PPRV infection of goats equipped with Ultra-Wideband sensors

Response to Editor and Reviewers

Dear Editor and Reviewers,

We thank you for your careful review of our revised manuscript entitled “How fast can PPRV spread? Analyzing the results of an experimental PPRV infection of goats equipped with Ultra-Wideband sensors” (Manuscript ID: PONE-D-26-21306R1). We have addressed all of the minor comments in the revised manuscript.

The Abstract has been revised to explicitly identify R_0 as the subject of the relevant sentence and to clarify that the reported values are derived estimates based on infectious-period assumptions from previous literature. The previous recommendation to extend quarantine to 30 days has also been moderated. The revised text now states that the adequacy of the current 21-day quarantine period specified in the WOAH Terrestrial Code warrants further evaluation and validation under field conditions, rather than implying an immediate policy revision.

The Discussion has been expanded to acknowledge the use of a single experimental PPRV strain, Senegal 20-GP, belonging to lineage IV, and to clarify that controlled experimental contact conditions may differ from natural contact patterns in pastoral systems, markets, and communal grazing. The need for further validation across additional strains or lineages and under field conditions is also stated. The Conclusion has been revised accordingly to clarify the scope and generalizability of our findings.

All corresponding changes have been incorporated into the revised manuscript. Given the minor and closely related nature of these comments, we have summarized the revisions rather than providing a separate point-by-point response.

We believe that the revised manuscript has been substantially strengthened thanks to the feedback received, and we hope that it now meets the journal's standards for publication.

Thank you once again for your consideration. We look forward to your favorable response and hope the revised manuscript can be accepted for publication in your esteemed journal.

Sincerely,
Manal NHILI
Email: manal.nhili@cirad.fr
Corresponding Author
