# Supplementary material for: How fast can PPRV spread? Analyzing the results of an experimental PPRV infection of goats equipped with Ultra-Wideband sensors
Source: PLoS One. 2026 Sep 23;21(9):e0358839. doi: 10.1371/journal.pone.0358839 (PMC13600536; doi:10.1371/journal.pone.0358839)
Supplement: Supplementary file 3 [file pone.0358839.s003.docx]

*PLOS ONE* Humane Endpoints Checklist

*PLOS ONE* manuscript number: ____**PONE-D-26-21306R2**___________________

**Complete the following if your study design includes death of a regulated animal as a likely outcome or planned experimental endpoint. Please also include all information in the Methods section of your manuscript.**

**ITEM 1.** **Describe whether humane endpoints* were used for all animals involved in the study.**

|  | **Recommendation** | **Section/Paragraph** |
| --- | --- | --- |
| **If humane endpoints* were used, report the following:** | | |
| **1** | **The specific criteria used to determine when animals should be euthanized: diarrhoea (in general, profuse diarrhoea is the last phase of the PPR)** | **Introduction:**  **PPR symptoms at page 3**  **S1 File at Section 1: Experiment design, Monitoring phase**  **Page 3** |
| **2** | **Once animals reached endpoint criteria, the amount of time elapsed before euthanasia: depending on the moment of the apparition of the diarrhoea** | **S1 File at Section 1: Experiment design, Monitoring phase**  **Page 3**  **Results:**  **Section 🡪 Clinical Observations and Serology results:**  **Page 24 and 25 (table 3)** |
| **3** | **Whether any animals died before meeting criteria for euthanasia: 3 animals were in that case (it has happened in night)** | **Results:**  **Section 🡪 Clinical Observations and Serology results:**  **Page 24 and 25 (table 3)** |
| **If humane endpoints* were not used, report the following:** | | |
| **1** | **A scientific and ethical justification for the study design, including the reasons why humane endpoints could not be used, and discussion of alternatives that were considered but could not be used** | **n.a.** |
| **2** | **Whether the institutional animal ethics committee specifically reviewed and approved the anticipated mortality in the study design** | **n.a.** |

**ITEM 2.** **Include the following details of the study design and outcomes.**

|  | **Recommendation** | **Section/Paragraph** |
| --- | --- | --- |
| **1** | **The duration of the experiment: five to eight weeks** | **S1 File at Section 1: Experiment design, Monitoring phase**  **Page 3** |
| **2** | **The numbers of animals used, euthanized, and found dead (if any); the cause of death for all animals: 6-7 animals for each experiment** | **Materials and methods**  **Pages 6-7**  **Section “Animals” at pages 8-7** |
| **3** | **How frequently animal health and behaviour were monitored: every day** | **S1 File at Section 1:**  **Section experiment design/** **Experiment infection phase/monitoring phase:**  **Pages 2-3** |
| **4** | **All animal welfare considerations taken, including efforts to minimize suffering and distress, use of analgesics or anaesthetics, or special housing conditions: special housing conditions, animal attendant specially and only dedicated to the experiment** | **S1 File at Section 1:**  **Section experiment design/** **Experiment infection phase/monitoring phase:**  **Pages 2-3**  **Results: table 3, pages 25-26** |
| **5** | **Any special training in animal care or handling provided for research staff: all staff involved in the experiment have been trained in animal handling. One the experiment supervisor has DVM and PhD degree with about 40 years’ experience in research on animal infectious pathogens (virus) and vaccine development with testing in animals (Adama Diallo). The second, Mr Moustapha Lo, PhD in immunology, was responsible for biosecurity and biosafety in ISRA/LNERV** | **Materials and methods Section Ethics statement:**  **Page 12** |

***Definition of a humane endpoint**

A humane endpoint is an experimental endpoint at which animals are euthanized when they display early markers associated with death or poor prognosis of quality of life, or specific signs of severe suffering or distress. Humane endpoints are used as an alternative to allowing such conditions to continue or progress to death following the experimental intervention (“death as an endpoint”), or only euthanizing animals at the end of an experiment. Before a study begins, researchers define the practical observations or measurements that will be used during the study to recognize a humane endpoint, based on anticipated clinical, physiological, and behavioral signs. These may include, for instance, body temperature or weight changes, tumor size or appearance, abnormal behaviors, pathological changes, ruffled fur, reduced mobility, body posture, or expression of specific body fluid markers. Please see the NC3Rs guidelines for more information.

**ARRIVE Guidelines**

*PLOS ONE* encourages authors to follow the [Animal Research: Reporting of In Vivo Experiments (ARRIVE) guidelines](http://www.nc3rs.org.uk/arrive-guidelines) for all submissions describing laboratory-based animal research and to upload a completed [ARRIVE Guidelines Checklist](http://www.nc3rs.org.uk/sites/default/files/documents/Guidelines/NC3Rs%20ARRIVE%20Guidelines%20Checklist%20%28fillable%29.pdf) to be published as supporting information.
