# Supplementary material for: How fast can PPRV spread? Analyzing the results of an experimental PPRV infection of goats equipped with Ultra-Wideband sensors
Source: PLoS One. 2026 Sep 23;21(9):e0358839. doi: 10.1371/journal.pone.0358839 (PMC13600536; doi:10.1371/journal.pone.0358839)
Supplement: Supplementary file 4 [file pone.0358839.s004.docx]

How fast can PPR Spread Analyzing the results of an experimental PPRV infection of goats equipped with Ultra-wide band sensors

| Line | Current | Recommendation |
| --- | --- | --- |
| Limited sample size and power concerns | 12 experiments, 70 naïve animals exposed. Only 18 infections observed (about 26%). | -Provide posterior uncertainty intervals for R₀ (only point estimates given in abstract).  -Present sensitivity analyses.  -Address identifiability issues. |
| The transmission treats each transmission as an independent | However:  -Animals are co-housed in a confined paddock.  -Transmission events are not independent.  -Shared exposure to a single seeder violates independence assumptions. | Therefore add discussion on:  -Cluster dependence  -Within-experiment correlation  -Random effects for experiment session |
| Incubation is inferred from  Symptom onset-  RT-PCR positivity-  Serology | Viral replication  Clinical manifestation  Sero-conversion | Pooling these will bias estimates |
| **Ethical**  **Reporting Incomplete**  Although institutional approval is mentioned: | Missing:   - Approval number   Explicit IACUC-equivalent body   - Humane endpoint criteria description - Clarification of why all animals were euthanized post-experiment |  |
