# Supplementary material for: How fast can PPRV spread? Analyzing the results of an experimental PPRV infection of goats equipped with Ultra-Wideband sensors
Source: PLoS One. 2026 Sep 23;21(9):e0358839. doi: 10.1371/journal.pone.0358839 (PMC13600536; doi:10.1371/journal.pone.0358839)
Supplement: Supplementary file 5 [file pone.0358839.s006.docx]

How fast can PPR Spread Analyzing the results of an experimental PPRV infection of goats equipped with Ultra-wide band sensors- 2^nd^ Review

| Location | Current | Why it is wrong | **Recommendation** |
| --- | --- | --- | --- |
| **Abstract** | "...we would recommend increasing the quarantine period to at least 30 days rather than the 21 days specified in the WOAH Terrestrial Code | This recommendation is not fully supported by the data.  Reasons:  -only 82 goats  -single lineage (Lineage IV)  -one experimental setting  -experimental rather than field conditions  -incubation estimate has uncertainty .  **Changing an international WOAH recommendation requires evidence from:**  -multiple studies  -field epidemiology  -outbreak investigations  -transmission modelling from naturally infected populations | "Our findings suggest that the adequacy of the current 21-day quarantine period deserves further evaluation and validation under field conditions." |
| In the text: | wording sometimes implies that the findings apply to **all** PPR viruses. | However, the study investigated  -one lineage  -one isolate  -One breed/environment | The conclusions should consistently specify:  "for the lineage IV strain evaluated." |
|  | The manuscript estimates  R₀ = 4.3–8.6 using infectious periods from previous literature. The authors correctly explain this |  | However, the **Discussion** should emphasize more clearly that  these are derived estimates, not directly observed values. |
| Methods | The manuscript could better acknowledge that  experimental contact  ≠  natural contact. | Goats under experimental conditions  -have restricted movement  -controlled environment  -known infection source  compared with  -pastoral systems  -markets  -communal grazing | The Discussion should explicitly mention this limitation. |
| Grammar | "could impact the transmission"  should be  "virus could circulate silently"  "great facility of movement"  should be | Should be | "could influence transmission"  "virus may circulate sub-clinically"  "high frequency of animal movement" |
