# Supplementary material for: How fast can PPRV spread? Analyzing the results of an experimental PPRV infection of goats equipped with Ultra-Wideband sensors
Source: PLoS One. 2026 Sep 23;21(9):e0358839. doi: 10.1371/journal.pone.0358839 (PMC13600536; doi:10.1371/journal.pone.0358839)
Supplement: Supplementary file 6 [file pone.0358839.s007.docx]

Authors’ Response

**Manal Nhili^*^, Jean-Baptiste Menassol, Gaye Laye Diop, Mariame Diop, Aminata Ndoye, Mbengué Ndiaye, Aminata Ba, Michel Dione, Assane Gueye Fall, Noha El Khattabi, Modou Moustapha Lo, Mounia Abik, Andrea Apolloni, Adama Diallo**

1 July 2026

**Manuscript ID:** PONE-D-26-21306 (previous ID: PONE-D-25-40904**)**

**Title of submission:** How fast can PPRV spread? Analyzing the results of an experimental PPRV infection of goats equipped with Ultra-Wideband sensors

**Response to Editor and Reviewers**

Dear Editor and Reviewers,

We sincerely appreciate the time and effort you have dedicated to reviewing our manuscript entitled “How fast can PPRV spread? Analyzing the results of an experimental PPRV infection of goats equipped with Ultra-Wideband sensors” (Manuscript ID: PONE-D-26-21306), and we are grateful for the constructive and insightful comments provided by the reviewers. Indeed, we have carefully considered all the comments and have revised the manuscript accordingly. We have addressed each point raised by the reviewers in a **point-by-point** response, which is provided below. Additionally, we have attached a clean version of the revised manuscript without any highlights for your convenience, and a revised manuscript with tracked changes to facilitate your review.
 We believe that the revised manuscript has been substantially strengthened thanks to the feedback received, and we hope that it now meets the journal’s standards for publication.
 Thank you once again for your consideration. We look forward to your favorable response and hope the revised manuscript can be accepted for publication in your esteemed journal.

Sincerely,
 Manal NHILI
 Email: [manal.nhili@cirad.fr](mailto:manal.nhili@cirad.fr)

**Response to Reviewer 1:**

We thank the reviewer for the positive evaluation and helpful suggestions.
**R1.1 Comment:** *Abstract: Shorten methodological detail; emphasize novelty and main findings instead of listing all numerical results.
Add a clear concluding statement linking findings to implications for PPRV control and spread.*

**Response:** We have shortened the abstract and added a sentence on the implications of findings to PPR spread and control. We believe that our findings could support update in Terrestrial Manual code regarding the increase of quarantine duration.

**For your convenience, the detailed modifications are highlighted below:**

**Page 2**

Peste des Petits Ruminants (PPR) is a highly contagious disease affecting goats and sheep. The speed and extent of its spread depend on contact patterns and the virus's intrinsic characteristics. To estimate its propagation speed and the role of inter-individual contacts, we conducted PPR virus transmission experiments in goats that were equipped with Ultra-Wideband (UWB) sensors to continuously track inter-individual distances alongside clinical and virological monitoring. Using Bayesian modeling, we integrated contact data and health status to estimate the basic reproduction number ($R_{0}$), the incubation period, and the virus transmissibility. The novel integration of distance tracking revealed that animal density in an area critically drives infection risk: the minimum exposure time that is required for a naïve animal to become infected dropped from nearly two days at low density to just four hours at high density. Overall, was estimated between 4.3 (95% HDI: 3.1 – 4.8) and 8.6 (95% HDI: 6.2 – 9.6) depending on the infectious period (5 and 10 days respectively). The incubation period was estimated to be around 14.7 days (95% CI 11.6 - 20.1). The exposure time varies greatly depending on the density, ranging from nearly two days at low density to around four hours at high density. These findings highlight high-density environments, such as conditions in livestock markets, as an important factor of PPRV transmission between animals and the disease spread. Considering the great facility of small ruminants’ movement on long distances, the high incubation period, about 15 days, might be one of the factors explaining the spread of PPR along long distances from the source of infection. To efficiently control and eradicate PPR, sheep and goats’ movement control must be one of the means to be applied. Based on our results, we would recommend increasing the quarantine period to at least 30 days, rather than the 21 days specified in World Animal Health (WOAH) Terrestrial Code.

**R1.2 Comment:** *Introduction: Condense the long epidemiological background (especially PPR history and lineage distribution). Move the study rationale and objectives earlier for clearer focus. Merge overlapping statements on lineage IV emergence and relevance to Senegal.*

**Response:** We condensed the epidemiological background and merged overlapping statements regarding lineage-IV. We also clarified the study gap and reorganized the text to ensure that the rational and study objectives are now clearly stated at the very end of the introduction. The introduction has now been reduced to the following:

**Lines 2-86 on Pages 4-7 (Section Introduction)**

Peste des petits ruminants (PPR) is a highly infectious and contagious disease affecting small ruminants. Clinically, it is characterized by fever, congestion of different mucosal membranes, followed by ocular and nasal discharges, then buccal erosive lesions and diarrhea at the late stage with death in some cases. Because of its morbidity rate that can be as high as 80-90% and a mortality rate as high as 50-80% depending on the epidemiological context, PPR is in the list of economically important animal diseases in which outbreaks must be reported to the World Organization for Animal Health (WOAH). The causative agent of this disease is a virus, the PPR Virus (PPRV). It is one of the members of the genus Morbillivirus in the family Paramyxoviridae, along with the rinderpest virus, the causal agent of rinderpest, the cattle and buffaloes disease that was officially eradicated globally in 2011 [1,2]. Because PPR and rinderpest share similar symptoms (ocular and nasal discharges, buccal erosive lesions and diarrhea), PPR was overlooked for a long time in favor of rinderpest. But gene sequence data that were available as of 1994 had showed that PPRV and RPV are distinct viruses but closely related [3]. In fact, looking carefully at the epidemiological history, both diseases have evolved independently [4]. From its historical stronghold endemic region in West Africa, PPR has expanded to most of African countries, except Southern Africa, the Middle and Near East, and most of the Asian countries (from China to Central Asia), and then some European countries [5,6].

The regions that are currently threatened by PPR are home to about 80% of the world's sheep and goat populations, and the annual losses that are caused by this disease were estimated in 2014 to be at the range of 1.2-2.8 billion [7]. Due to its significant economic impact, particularly on small farmers who rely mainly on sheep and goats for their livelihood in low-income countries, the Food and Agriculture of the United Nations (FAO) and World Organization for Animal Health (WOAH) convened an international conference in 2015 on PPR, conference during which was adopted a strategy for the global eradication of this disease by 2030: the PPR Global Control and Eradication Strategy (PPR-GCES) [8].

PPRV strains that have been identified so far have been grouped into four genetic lineages (I, II, III and IV) based on the virus gene sequence data [9]. Initially, these lineages occupied distinct geographical distributions across West Africa, East Africa and Asia. The lineage IV which is also named the Asian PPRV lineage as it was present only in Asia and the Middle East, but not in Africa, expanded its geographic distribution to that continent since year 2008, either replacing pre-existing lineage strains in some countries or co-existing with pre-existing lineage strains in some other countries [10–13]. For the moment, it is not known the factors behind the PPRV lineages geographical changes and the apparent advantages of lineage IV over to other lineages.

Knowledge of basic parameters of the PPRV transmission from host to host and understanding the virus spread might be useful in describing the transmission dynamics and supporting the PPR eradication program for more efficiency [14]. In 2018, Fournié et al. [15] proposed a mathematical model of PPRV transmission and elimination in Ethiopia based only on PPR serological surveillance data without any PPRV transmission experiment. Few years later, Herzog et al. [16,17] reported PPRV transmission experiments they conducted on both cattle and domestic small ruminants with a view to studying the potential involvement of cattle in PPR epidemiology. They concluded that cattle play a negligible role in PPRV circulation, even though PPRV-excreting small ruminants can infect cattle but without any overt disease. That transmission seems to have a probability much lower than that of small ruminants to small ruminants transmission. To our knowledge, there are currently no reports estimating PPRV transmission parameters in small ruminant populations under controlled experimental conditions. With that objective in mind, we conducted PPRV transmission experiments on goats to estimate basic epidemiological parameters, like contact rate and incubation period, as well as the minimum exposure time (i.e., the minimum time a naïve animal should be in contact with an infected one to get infected) and the influence of contact patterns between animals on virus transmissibility. Based on the results we obtained we propose a model for PPRV transmissibility. As the PPRV lineage IV seems to be the group of the most aggressive PPRV strains, continuously expanding its geographical distribution compared to other PPRV lineages, our experiments were conducted with a strain of that group. It was isolated during a PPR outbreak in 2020 in Senegal.

For our experiments, we equipped animals with UWB and Radio-frequency identification (RFID) devices that have been widely used to study interaction among individuals. Adapted to working in confined environments, this type of device provides more detailed and frequent information than GPS. The signals of the two devices are frequently triangulated, and the distance between their bearers is estimated at each time step. The result is a dynamic contact network among individuals. UWB and RFID devices have been widely used to study interactions among individuals (humans) in different settings like social events, conferences [18,19], hospitals [20], schools [21,22], and villages [19,23,24]. The results provide information for studying the interactions among individuals and retrieving information about possible drivers. Similarly, UWB devices have been recently applied to study animal behavior and welfare for different animal species, dogs, chickens, sheep, cows, and horses [25–33].

Due to the transmission mechanisms, we suppose that the likelihood of getting infected depends on the contact pattern between animals and could increase with exposure time. In our study, we tested these hypotheses by analyzing data from experimental infections where animals were equipped with UWB devices to collect information about contacts, and a set of statistical models was developed to infer the value of the virus’s transmissibility. The primary objectives of our study were: (i) to estimate the transmission rate and basic reproductive number (R_0) of the PPRV lineage IV; (ii) to characterize the distribution of the incubation period from experimental infection data; (iii) to estimate the minimum exposure time required for infection under different distance thresholds. Furthermore, we assessed whether UWB-derived distance information improved interpretation relative to duration-only models. To our knowledge, this is the first time that this kind of study, combining infection monitoring and contact patterns, has been performed for PPR. By taking account of contact variation, the analyses could help provide more accurate estimates of transmission probabilities and, consequently, the impact of PPR.

**R1.3 Comment:** *Materials and Methods: Streamline repetitive procedural descriptions (e.g., infection, exposure, monitoring).
Move excessive manufacturer, equipment, and coordinate details to supplementary material. Summarize UWB system setup focus on performance (accuracy, sampling rate) rather than hardware specifications.
Add a schematic summarizing the modelling framework (Baseline →Segmented → Stratified → Envelope).
Ensure all equations are properly formatted and numbered consistently.*

**Response:** We thank the reviewer for these constructive suggestions to improve the readability and flow of the Materials and Methods section. We have addressed each point as follows:

- *Streamlined procedures and moved details to Supplementary Material:* We agree that the procedural descriptions were overly detailed for the main text. We have streamlined this section by moving the detailed descriptions of the "Experiment infection phase", "Exposure phase", and "Monitoring phase" to the Supplementary Material (S1 File.docx at Section 1).
- *Summarized UWB system setup:* We have revised the UWB system setup section in the main text. As suggested, we removed the granular hardware specifications and refocused the description on the system's performance metrics, specifically highlighting its accuracy and sampling rate.
- *Modelling framework schematic:* We would like to clarify that a schematic summarizing the modelling framework (Baseline → Segmented → Stratified → Envelope) is already included in the manuscript as Fig 2. We have reviewed the text to ensure that Fig 2 is clearly referenced at this stage so readers can easily follow the modelling progression.
- *Equation formatting and numbering:* We have carefully reviewed the mathematical sections. All equations have been revised, properly formatted, and numbered consistently throughout the manuscript.

**For your convenience, the modifications are highlighted below:**

**Lines 146-158 on Page 10 (Section: Experiment design)**

Each experience was divided into three phases as shown in Fig 2:

1. Experimental Infection phase: infection of animal to be used for transmission of the PPRV to naïve animals by contact: the seeder.
2. Exposure Phase: The seeder is in contact with other naïve animals to allow transmission from animal-to-animal in a paddock for a period of variable duration.
3. Monitoring phase: exposed animals are isolated and monitored until they show PPR symptoms or any other PPR test positive results.

**Fig 2. Detailed Schema of the Experimental Infection Protocol for Studying PPR lineage IV Transmission Dynamics.** This diagram outlines the sequential stages of the experimental protocol designed to investigate the transmission of PPR among goats. The protocol includes three primary periods: the experimental infection period, the exposure period, and the monitoring period.

Detailed procedures for each experimental phase are provided in the S1 File, Section 1.

**Lines 160-196 on Pages 10-12 (Section: Data collection for indoor positioning)**

UWB solutions are used to measure the distance between a reference sensor (referred to as an anchor) and a sensor whose position is to be determined (referred to as a tag, equipped on the animal) using different metrics. For these experiments, paddock 1 was equipped with six anchors (UWB BeSpoon - STMicroelectronics, France) fixed to the ceiling, one at each corner and two near the center of the room. Each anchor was powered by a Power Over Ethernet cable connected to a dedicated 16-port switch (DES-1018MP; D-Link, Taipei - Taïwan). The tags (BeSpoon Industrial Tag; Length: 101mm, Width: 52mm, Thickness: 25mm; BeSpoon - STMicroelectronics, France) were enclosed in a custom 3D-printed case to provide shock protection and ensure better attachment to the collar. The UWB system was controlled via a dedicated local server (a laptop with a Linux operating system), which provided a user interface for verifying the general system status and initiating data acquisitions. This experimental setup was designed to monitor and record, in near-real time, the precise indoor location of each individual.

The theoretical performance of the solution allows positioning of an object with an accuracy of +/- 20 centimeters, but these performances depend notably on the tracking environment and the deployment conditions. Therefore, in order to evaluate the performance of this indoor positioning system under our experimental conditions, we designed and implemented a custom evaluation protocol using the different available tags (n = 7). Accuracy and precision were assessed using pairwise inter-sensor distance measurements across multiple geometric configurations:

- Sensors arranged in a straight line on the ground, with inter-sensor distances of 6, 10, 15, 30, and 50 cm, respectively.
- Sensors arranged in a straight line on a wooden bench at a height of 51.6 cm (approximating their actual monitoring height when equipped on the animals), with inter-sensor distances of 20 cm.
- Sensors arranged in a hexagon configuration with one sensor at the center and inter-sensor distances of 15, 30, and 100 cm, respectively.

Across all configurations, the system demonstrated a mean accuracy of 28.3 ± 6.2 cm and a mean precision of 22.2 ± 4.8 cm. These results were consistent with the manufacturer’s specifications and met our experimental requirements.

During the monitoring phase, the acquisition frequency was set to f = 1 Hz (one acquisition per second). Data were stored on the server as a csv file, with each line representing the estimated position of each tag on the x, y, and z axes. Distances between the infected individual and each susceptible animal were then computed from these data, using only the x and y axes. This frequency of indoor positioning provided a comprehensive temporal map of animal interactions. High-resolution proximity data enabled us to analyze not only the frequency of close contacts, but also the duration of these interactions at different distance thresholds.

**R1.4 Comment:** *Simplify numeric descriptions use summary terms (“higher,” “longer,” “comparable”) instead of repeating raw values. Strengthen interpretative commentary linking behavioural contact data to transmission outcomes.
Verify figure order and in-text citations for consistency.
Consider merging Figures 4 and 5 or moving detailed distribution plots to supplementary materials.*

**Response:** We thank the reviewer for these suggestions. We have simplified numeric descriptions using summary terms, verified figure order and citations for consistency, and moved the detailed distribution plots to the Supplementary Information (S1 File, Section 2), with the former Figures 4 and 5 now presented as Figures S2 and S4 respectively.

Furthermore, we strengthened the interpretative commentary linking behavioral data to transmission outcomes by introducing the concept of a "spatial buffer" and explicitly connecting it to our Stratified 2 model results. The revised paragraph now reads:

**Lines 750-769 on Pages 39-40 (Section: Discussion)**

Our results indicate that individuals spend a very short amount of time—just a few minutes—close to each other. Moreover, the analysis of the temporal contact network revealed no clear contact patterns, suggesting that animals tend to avoid close interactions. **This behavioral tendency to maintain distance effectively created a protective “spatial buffer” within the enclosure.** This behavior may be due to a lack of familiarity, the animals in our study were young and, coming from different herds, and were not previously acquainted. This could have influenced their social behavior and reduced their gregariousness, especially considering that young animals typically remain close to their mothers and siblings. **Consequently, the force of infection was not a continuous low-level stream, but rather a series of rare, high-intensity events. This behavioral “spatial buffering” explains why no transmission events were recorded in experiments lasting fewer than 24 hours; the duration was simply insufficient for random movement to eventually force a high-risk close-contact event. However, our Stratified 2 model identifies these rare, brief windows of face-to-face proximity (< 0.5 m) as the primary drivers of the outbreak, carrying a per-minute transmission probability (p) 39 times higher than the facility-wide average. This behavioral-probabilistic link suggests that PPRV is driven by discrete, high-risk interactions during spontaneous fluctuations in proximity, a dynamic that would rapidly collapse in high-density settings such as livestock markets.** We are currently analyzing the footage by cameras that were in the animal facility. The results, to be reported in a second manuscript, will give more information about the contacts between animals in the same premises.

**R1.5 Comment:** *Discussion: Begin with a strong interpretative paragraph highlighting how findings advance current understanding of PPRV spread.
Add a short limitations paragraph (e.g., small sample size, single strain, confined experimental setting).
Clarify how exposure-time estimates and contact density findings could inform outbreak modelling and control strategies.
Remove repetitive restatement of numerical results already covered in Results.*

**Response:** We thank the reviewer for these constructive suggestions, which have greatly improved the focus and flow of the Discussion. We have addressed each point as follows:

We have revised the opening paragraph of the Discussion to provide a strong, clear interpretation of how our findings advance the current understanding of PPRV transmission and spread.

**Lines 658-664 on Page 35 (Section Discussion)**

This study shows that controlled PPRV infection experiments, combined with high-resolution UWB proximity data, can improve estimations of the transmission parameters and show how contact structure shapes infection risk. The main finding of our study is that exposure duration alone explained much of the observed infection pattern, but distance-resolved data were essential for translating that risk into biologically meaningful scenarios, especially when animals were densely grouped. This is relevant for pens, transport, and livestock markets, where short-range contacts may accumulate over several hours.

We have added a dedicated paragraph outlining the limitations of our study, specifically addressing the small sample size, the use of a single strain, and the confined experimental setting.

**Lines 805-812 on Page 41 (Section Discussion)**

Despite these advancements, our study has several limitations. The experimental design involved a small sample size and a single PPRV strain, conducted within a confined experimental setting. While this controlled environment allows precise measurement of inter-animal distances and transmission events, it may not fully reflect the complexities of field scenarios, where environmental factors, seasonal variations, and diverse husbandry practices can influence transmission dynamics. Future research should aim to validate these findings in larger, more diverse populations under varying field conditions and also with virus strains from other PPRV lineages.

We removed the previous text regarding exposure duration, as it read too much like a methodological description. In its place, we added a new paragraph that clearly interprets how our exposure-time estimates and contact density findings can directly inform outbreak modelling and the design of control strategies.

**Lines 793-803 on Page 41 (Section: Discussion)**

Our findings on minimal exposure time estimates provide actionable insights for outbreak modeling and control strategies. These results demonstrate that significantly less exposure time is required for transmission in scenarios with higher contact density. For example, the minimal exposure time can scale by a factor of four from a highly dense herd (distance < 0.5m, density = 5 animals per m^2^) to a herd that is almost 35 times less dense, or by a factor of 12 when animals move randomly in a 100 times larger space. This translates directly into practical scenarios: in lower-density free-grazing systems, animals might require longer periods of interaction for transmission to occur, whereas in higher-density pen or market settings, even brief encounters could pose a substantial risk. These findings strongly support the effectiveness of physical distancing measures and provide valuable insights into safety protocols in controlled environments.

**Response to Reviewer 2:**

We thank the reviewer for the positive assessment of our study and for the constructive comments. We have revised the manuscript to address the points raised, specifically improving the clarity, methodological explanations, and interpretation. Our point-by-point responses are detailed below.

**R2.1 Comment:** *The author should clearly mention the study objectives.*

**Response:** At the end of the introduction, we have clearly indicated that knowledge parameters of the PPRV that might explain its transmission from host to host and spread are missing and that our objective is to study some of those parameters that might help understanding/explaining the virus spread such as: R_0_, contact rate and incubation period, the minimum exposure time.

**For your convenience, the modifications are highlighted below:**

**Lines 74-86 on Page 7 (Section: Introduction)**

Due to the transmission mechanisms, we suppose that the likelihood of getting infected depends on the contact pattern between animals and could increase with exposure time. In our study, we tested these hypotheses by analyzing data from experimental infections where animals were equipped with UWB devices to collect information about contacts, and a set of statistical models was developed to infer the value of the virus’s transmissibility. The primary objectives of our study were: (i) to estimate the transmission rate and basic reproductive number ($R_{0}$) of the PPRV lineage IV; (ii) to characterize the distribution of the incubation period from experimental infection data; (iii) to estimate the minimum exposure time required for infection under different distance thresholds. Furthermore, we assessed whether UWB-derived distance information improved interpretation relative to duration-only models. To our knowledge, this is the first time that this kind of study, combining infection monitoring and contact patterns, has been performed for PPR. By taking account of contact variation, the analyses could help provide more accurate estimates of transmission probabilities and, consequently, the impact of PPR.

**R2.2 Comment:** *Describe the justification of assumptions used for R₀ estimation (particularly the infectious period)*

**Response:** We thank the reviewer for this important point. Given the nature of the disease and the absence of age distribution data, the model can be accurately approximated using a Susceptible-Infectious-Recovered (SIR) framework, consequently $R0=\beta_{d}/\gamma$. The transmission rate, $\beta_{d}$ is the transmission parameter at daily timescale, and has been evaluated from the (hourly) experimental one using the scaling law. Recovery rate was not estimated within the scope of this study, since animals were euthanized before they recovered. Instead, we used established values for the infectious period from the literature, which are commonly employed in PPR mathematical modeling. We have stated in the text that the infectious period is derived from the literature.

**For your convenience, the modifications are highlighted below:**

**Lines 256-265 on Page 15 (Section Materials and Methods)**

In a continuous transmission model, considering an exponential process, the daily transmission rate $\beta_{d}$ can be estimated from the hourly transmission rate $\beta_{h}$as:

$$\begin{aligned} \beta_{d}= 1 - e^{-\beta_{h} 24} \#\left( 2 \right) \end{aligned}$$

Based on this, a first estimate of the basic reproductive number $R_{0}$ was calculated using the following formula derived from the SIR model definition:

$$\begin{aligned} R_{0}=\frac{\beta_{d}}{\gamma} \#\left( 3 \right) \end{aligned}$$

where, $\beta_{d}$ is the daily transmission rate, and γ stands for the recovery rate (the inverse of the infectious period) commonly used in PPR modeling. The infection period could not be estimated from our experimental settings. Therefore, we calculated $R_{0}$ using two infectious period estimates from the literature (5 and 10 days).

**R2.3 Comment:** *Explain clearly how “minimum exposure time” was inferred, given the experimental results.*

**Response:** We thank the reviewer for raising this point. We added a dedicated subsection entitled “Exposure time estimation” under the global section “Materials and methods” explaining that minimum exposure time was inferred by simulation from posterior transmission-probability estimates. The revised text states that these are model-based contact durations expected to produce later confirmed infection under assumed contact and density conditions, not directly observed infection times.

**For your convenience, the modifications are highlighted below:**

**Lines 355-381 on Pages 19-20 (Section Materials and methods)**

**Exposure time estimation**

To estimate the minimum exposure duration required for a susceptible animal to become infected, we simulated various contact scenarios by analytically inverting the cumulative probability of infection functions derived from our best-fitting transmission models. The cumulative probability of infection $P(t)$ after an exposure time $t$ (measured in minutes) was modeled under two main assumptions regarding animal movement and spatial density:

First, to represent a scenario where animals move freely and interact at varying distances without extended close contact (e.g., roaming herds), we used the Baseline model (see Eq. (4))

Second, to represent high-density environments where animals are maintained at relatively fixed, close distances (e.g., crowded livestock markets or transport), we used the Stratified models. For a susceptible animal kept at a constant distance within a specific spatial interval $k$, the cumulative probability of infection simplifies to:

$$\begin{aligned} P\left( t \right)= 1 - \left( 1-\alpha_{k}p \right)^{t}\#\left( 7 \right) \end{aligned}$$

In this formulation, $p$ represents the reference transmission probability for the baseline distance interval $j$ and scaling factors $\alpha_{k}$.

For both scenarios, the minimum exposure time $t$ required to reach a specific probability of infection threshold $P$ (e.g., 50% and 95%) was calculated by solving the cumulative probability equations.

$$\begin{aligned} t = \frac{\ln\left( 1-P \right)}{\ln\left( 1-p_{eff} \right)}\#\left( 8 \right) \end{aligned}$$

Where $p_{eff}$ is the effective transmission probability per minute ($p_{eff}= p$ for the Baseline model, and $p_{eff}=\alpha_{k}p$ for the Stratified models).

We used the posterior mean and 95% HDI estimates of the transmission probabilities ($p$) and scaling factors ($\alpha$) obtained from the Bayesian inference of the models. We used the Baseline model to estimate exposure time for the unrestricted movement scenario. For fixed distances, we used the Stratified 1 model for the 0.05-0.5 m range, and the Stratified 4 model for the remaining distances (0.5-1 m, 1-2 m, and ≥ 2 m). Finally, we converted all exposure times from minutes to hours to make them easier to compare across the different density scenarios.

**R2.4 Comment:** *Discuss the justification of Bayesian model structure*

**Response:** We thank the reviewer for this comment. Currently, there is no consensus in the literature regarding what constitutes an "effective contact" for PPRV. It was necessary to test several plausible models that account for different definitions of effective contact. In some of contact tracing cases, "effective contact" is typically defined by simple, binary thresholds (e.g., being within 1 meter for a set time), which our Baseline and Segmented models test to establish a standard epidemiological benchmark. However, biologically, the risk of respiratory or close-proximity transmission does not abruptly drop to zero at a specific cutoff; rather, viral concentration decays continuously as distance increases. By progressively moving to Stratified and Envelope (continuous decay) models, we could evaluate whether capturing this continuous physical reality significantly improved our predictive power compared to standard uniform mixing assumptions. To clarify this, we added a model roadmap in Table 1 and a short justification for the Bayesian framework choice and structure.

**Lines 216-229 on Pages 12-13 (Section: Materials and Methods)**

We employed a Bayesian statistical framework to estimate the transmission rate, incubation period, and model transmission probability, **a choice driven by the need to integrate high-resolution individual proximity data with batch-level and individual level infection outcomes. This probabilistic approach is particularly suited to experimental datasets with a limited number of independent batches and small sample size, as it avoids reliance on large-sample asymptotic assumptions and allows for the rigorous quantification of uncertainty through posterior distributions. By employing a Bayesian structure, we defined model-specific priors to ensure biological plausibility.** Depending on the particular quantity to estimate, we used different likelihoods to characterize the binary outcome of infection status and the duration of the incubation phase. In all cases we used minimally informative prior distributions to minimize potential bias and applied the Markov Chain Monte Carlo (MCMC) approach to estimate model parameters. **Table 1 summarizes the four transmission probability models described in this section, detailing their respective outcomes, predictors, parameters, and epidemiological assumptions.**

**Response to Reviewer 3:**

We thank the reviewer for the feedback. To answer clearly and extensively, we present the remarks made in the form of concerns and recommendations provided

- Limited Sample size and power concerns: 12 experiments with 70 naïve animals exposed, and only 18 infections observed (about 26%)

Experimental infection studies are designed to reduce the number of animals but at the same time provide statistical power. A batch of 4-6 animals has been judged reliable to provide useful information, while reducing the use of animals, and has been used in some of recent articles of small ruminant infections. Our statistical power analysis (Fig S6 in S1 File, Section 5) has shown that our experimental apparatus is able to detect with 80% an infection occurring with a probability as low as 0.03.

Laws, T.R. and Maishman, T.C. (2022) ‘Considerations in the design of animal infection pilot studies’, *Frontiers in Cellular and Infection Microbiology*, 12. Available at: <https://doi.org/10.3389/fcimb.2022.948464>.

Milovanović, M. *et al.* (2022) ‘The Experimental Infection of Goats with Small Ruminant Morbillivirus Originated from Barbary Sheep’, *Pathogens*, 11(9), p. 991. Available at: <https://doi.org/10.3390/pathogens11090991>.

Truong, T. *et al.* (2014) ‘Peste des Petits Ruminants Virus Tissue Tropism and Pathogenesis in Sheep and Goats following Experimental Infection’, *PLoS ONE*, 9(1), p. e87145. Available at: <https://doi.org/10.1371/journal.pone.0087145>.

- **Recommendations:**
  - **Provide posterior Uncertainty intervals for R_0_**

The basic reproductive ratio R₀ has been calculated using the estimated value for the transmission parameter obtained from the calibration, and the two infection periods used in the modelling papers: 5 and 10 days. Consequently, the posterior distribution of R_0_ coincides with the posterior distribution of the transmission parameter up to a scale factor. The average values of R_0_ calculated in the two conditions are presented throughout the paper together with their HDI: “4.3 (95% HDI: 3.1–4.8) for an infectious period of 5 days, and 8.6 (95% HDI: 6.2–9.6) for an infectious period of 10 days”.

- **Present sensitivity analysis and Address identifiability issues.**

We deliberately chose a Bayesian framework so that uncertainty in model parameters is explicitly represented by probability distributions. These distributions are then used to produce output distributions that inherently account for variability and uncertainty, thereby reducing the need for separate deterministic sensitivity analyses and helping to address potential parameter identifiability issues. We explored several prior distributions for the transmission parameter; however, they produced similar results, so we have chosen to present only those with the lowest AIC. For each distribution, multiple Markov chains were run from different initial parameter values to explore the parameter space and to assess convergence. In our model, epidemiological initial conditions (a single infected animal) were fixed à priori.

- **The transmission treats each transmission as an independent However: -Animals are co-housed in a confined paddock; Transmission events are not independent; Shared exposure to a single seeder violates independence assumptions.**
- **Recommendations: add discussion on:**
  - **Cluster dependence**
  - **Within-experiment correlation**
  - **Random effects for experiment session**

We agree with the reviewer that, since the animals shared the same location and were exposed to the same source at the same time, the results of the infections are correlated. To assess the role of cluster dependence and within-experimental correlation, we ran a series of statistical models with random effects.

We considered the infectious status at the end of the experiment and used a logistic model to assess the effect of the experiment and of exposure to different sources. In this case, we used the experiment ID as the random effect (risk unit). We considered the cumulative and average exposure to the source, which was estimated based on the distance between the source and the animal, as well as the duration of the experiment. The random effects model is significant at 10%, indicating that there is huge variance between the experiments. However, when accounting for different exposures, cumulative exposure was found to have a significant effect, reducing the variance of the fixed effect by more than 54%.

In both cases differences between experiments reduced when we consider long duration experiments (24 hours or more)

**The choice of the models used is presented in the Materials and methods section as:**

**Lines 288-297 on pages 16-17 (section: Materials and methods)**

We studied the effect of cluster dependence and within-experiment correlation on infectious status of the animals.

We used logistic regression model with a random effect, using the predicted variable as the individual infectious status and the exposure measure (${exposure}_{j})$ as a predictor of infectious status.

$$\begin{aligned} \log\left( p_{ij} \right)=\beta_{0}+\beta_{1} exposure_{j}+b_{j}+\epsilon_{ij} \#\left( 4 \right) \end{aligned}$$

Exposure was considered to be the inverse of the distance from the source of infection at each time point, and we considered both the cumulative exposure and the duration of the experiment. We then performed a comparison between the different models using ANOVA test, to find if any difference existed between the different experiments.

Further results are detailed in Section 6 of the Supplementary Information (S1 File), the complete analysis table is provided in Table S1 in the same section (S1 File).

- **Incubation is inferred from Symptom onset RT-PCR positivity and Serology. Pooling these will bias estimates**

Of all the infected animals (i.e. those with a positive serology result at the end of the experiment), only five exhibited symptoms or tested positive in an RT-PCR test. This occurred an average of 11 days after exposure, with a range of 7–17 days. The same animals tested seropositive around two days later on average (1.92 days). Animals that did not exhibit symptoms became seropositive within an interval of 9–37 days after exposure phase.

To improve the estimation of the incubation period, we only included seropositive animals. A survival model was calibrated under the assumption that the onset of symptoms preceded seroconversion by a short and variable delay. This delay was modelled using a normal distribution parametrized using data from five symptomatic animals (mean = 1.92 days, standard deviation = 2.8 days). Model calibration was performed using ten Markov chains of 5000 iterations each. We tested several models for the survival analysis, but similarly to the previous case the log-normal model outperformed. The median of the incubation period was 15 days with 95% HDI (11.8; 20).

**A paragraph has been added in the material and method section:**

**Lines 277-282 on Page 16 (Section: Materials and Methods)**

To improve the estimation of the incubation period, we only included seropositive animals. A survival model was calibrated under the assumption that the onset of symptoms preceded seroconversion by a short and variable delay. This delay was modelled using a normal distribution parametrized using data from five symptomatic animals (mean = 2.3 days, standard deviation = 2.9 days). Model calibration was performed using ten Markov chains of 5000 iterations each.

**And in the results section:**

**Lines 510-512 on page 27 (Section: Results)**

This occurred an average of 11 days after exposure, with a range of 7–17 days. The same animals tested seropositive around two days later on average (2.3 days). Animals that did not exhibit symptoms became seropositive within an interval of 9–37 days after exposure phase.

Together with the new estimates of the incubation periods.

**Ethical Reporting Incomplete Although institutional approval is mentioned:** Missing:

- Approval number
- Explicit IACUC-equivalent body
- Humane endpoint criteria description
- Clarification of why all animals were euthanized post-experiment

**Information on the ethical issues were provided during the first submission of the manuscript and were approved by the editors.**

**Response to Reviewer 4:**

We thank the reviewer for the positive assessment and for identifying the main areas that needed clarification. We revised the Introduction to state the research questions and hypotheses more clearly, clarified the statistical framework and endpoint definitions, expanded the discussion of uncertainty and limitations, and added biological interpretation of the distance and density dependent findings.

**Major Comments:**

**R4.1 Major Comment:** *The Introduction provides useful background on PPR and lineage IV and explains that the manuscript is structured in two parts (classical epidemiological parameters and contact-pattern-based models), but the main research questions and hypotheses are not stated clearly enough. I recommend adding a short paragraph at the end of the Introduction (e.g., after the paragraph beginning “In our study, we analysed data from experimental infections…” on pp. 6, lines85–95) to explicitly clarify the main research questions and hypotheses by stating the primary objectives (e.g., estimation of β and R₀, characterization of the incubation period, quantification of distance- and time-dependent transmission, and estimation of minimal exposure times under different density conditions), the secondary or exploratory objectives (e.g., assessing the added value of UWB distance information relative to duration-only models), and the main a priori expectations. This would make the study design easier to follow and help readers interpret the modelling results more directly.*

**Response:** We appreciate the reviewer pointing this out. We added an explicit objectives paragraph at the end of the Introduction, including primary objectives, the secondary/exploratory objective, and the main expectations.

**For your convenience, the modifications are highlighted below:**

**Lines 74-86 on Page 7 (Section: Introduction)**

Due to the transmission mechanisms, we suppose that the likelihood of getting infected depends on the contact pattern between animals and could increase with exposure time. In our study, we tested these hypotheses by analyzing data from experimental infections where animals were equipped with UWB devices to collect information about contacts, and a set of statistical models was developed to infer the value of the virus’s transmissibility. The primary objectives of our study were: (i) to estimate the transmission rate and basic reproductive number ($R_{0}$) of the PPRV lineage IV; (ii) to characterize the distribution of the incubation period from experimental infection data; (iii) to estimate the minimum exposure time required for infection under different distance thresholds. Furthermore, we assessed whether UWB-derived distance information improved interpretation relative to duration-only models. To our knowledge, this is the first time that this kind of study, combining infection monitoring and contact patterns, has been performed for PPR. By taking account of contact variation, the analyses could help provide more accurate estimates of transmission probabilities and, consequently, the impact of PPR.

**R4.2 Major Comment: 2.**	*The “Statistical analysis and models” section (beginning around pp. 14) includes several important components (transmission rate/R₀, incubation period, and the baseline/segmented/stratified/envelope models), but it remains dense for non-specialist readers. Although Tables 4 and 5 summarize priors and posterior estimates, they do not provide a clear model-level roadmap.*

*I recommend adding a brief overview table or schematic at the start of this section summarizing, for each model, the outcome, predictors, likelihood, key parameters (e.g., βₕ, β, R₀, p, P₀, λ, αᵢ), and epidemiological interpretation.*

*The notation should also be made fully consistent across subsections, particularly for βₕ versus β, p versus P versus P₀, and αᵢ/dᵢ/tᵢ/λ/P₀ (pp. 14-18).*

*In addition, the Results section should consistently state which model is being used for each estimate (e.g., Table 5, pp. 28–29) and briefly restate the key assumptions (distance bands vs. continuous envelope).*

*As currently presented, Table 5 combines posterior estimates from different model classes, but the accompanying text does not always restate on the relevant assumptions, making it easy to compare p estimates across models even when their definitions differ.*

**Response:** We revised the beginning of the Statistical analysis and models’ section to provide a model-level roadmap in Table 1. We also standardized the notation throughout the manuscript to ensure consistency across all subsections: $\beta_{d}$ and $\beta_{h}$ now explicitly denote the daily and hourly transmission rates, respectively; *p* represents the probability of successful transmission per minute; *P* denotes the cumulative probability of infection per individual; and *p₀* represents the maximum probability of transmission at a distance of zero (envelope model intercept).

To further clarify the assumptions underlying each model and prevent erroneous cross-model comparisons of parameter estimates, we added a “Assumption” column to Tables 5 and 6, and we included a parameter definition table (Table S11) in the Supplementary Information (S1 File, Section 9) detailing the mathematical role, spatial condition, and epidemiological meaning of every parameter across all model classes.

**R4.3 Major Comment:** *Some technical details could be moved to the Supplementary Material to improve the flow of the main text without compromising reproducibility.*

*In particular, this includes the Jaccard-based “era” clustering in the “Network analysis of the contact pattern” subsection (pp. 13, lines 247-266) and the full envelope-model formula (pp. 19). A short conceptual description can remain in the main text, while the full technical formulation is moved to the Supplementary Material.*

**Response:** We agree with the reviewer that relocating these details improves the flow and readability of the main text. We have moved the full technical formulation of the envelope model and the detailed Jaccard-based "era" clustering methodology to the Supplementary Material (S1 File.docx).

**The conceptual description now included in the main text reads:**

**Lines 349-354 on Page 19 (Section: Materials and methods)**

**Envelope model**

Our most sophisticated approach, the envelope model, integrates both temporal and spatial proximity data clustering. We identified distinct envelop interaction groups for each individual and used the total duration of these groups along with their median distances to estimate infection probability. This model was applied with different distance filters (≤ 1 m, ≤ 1.5 m, and ≤ 2 m). The mathematical formulation is detailed in (S1 File, Section 3).

**R4.4 Major Comment:** *This study appears to use serology as the principal infection endpoint in most quantitative analyses, with RT-PCR results and clinical signs incorporated primarily as complementary evidence. This approach is acceptable; however, it would benefit from more explicit statement and consistent application throughout the manuscript. To improve clarity and reproducibility, the authors are encouraged to add a brief paragraph in the “Statistical analysis and models” section (pp. 14) specifying the primary endpoint used in each analysis, defining a prespecified infection classification rule (e.g., serology and/or RT-PCR, including post-mortem PCR where applicable), and briefly clarifying the biological interpretation of each endpoint (e.g., prior infection/seroconversion, viral shedding, clinical disease).

In addition, because seroconversion occurs after infection, the authors should clarify how this temporal lag was addressed in models estimating minimal exposure time (most likely in the exposure-threshold or proximity-based modelling subsections) and provide a cross-reference to the relevant model paragraph.*

**Response:** We thank the reviewer for this suggestion, which improves the methodological transparency of our infection outcomes. We have added a new subsection titled “Infection classification and endpoint” placed just after the roadmap Table 1. We defined our classification rule as: an animal is considered infected if it demonstrates seroconversion, viral shedding (RT-PCR), or post-mortem PCR positivity.

Serology is the main criteria indicating host infection by PPRV as they are many cases of inapparent infections and those cases are discovered only after serological surveillance (we have also noted those cases during our study)

Regarding the temporal lag of seroconversion: we have clarified in the text that this lag does not bias our estimates of **minimal exposure time**.

- **Transmission event:** In our experimental design, the "viral shedding" could only have occurred during the fixed exposure window (1 to 48 hours) while the seeder was in the paddock. Our models were designed to calculate the probability that this event occurred **strictly within** **those tracked hours** based on UWB proximity data.
- **Serology:** Seroconversion proves that the event happened. Whether the antibodies are detected on Day 14 or Day 21 (the temporal lag) does not change the fact that the animal was infected during the “exposure phase” period tracked by UWB sensors.
- **Statistical independence:** Because our outcome is a **binary status** (Infected = 1, Naïve = 0) calibrated against a **fixed exposure window**, the time elapsed **after** that window to reach seropositivity is a constant that applies to all infected animals. It informs the **Incubation Period** (how long the virus takes to manifest) but does not bias the **transmission probability (p)** (how likely the virus was to jump during a specific minute of contact).

**For your convenience, the added subsection now reads:**

**Lines 232-244 on Page 14 (Section: Materials and methods)**

**Infection classification and endpoint**

To ensure consistency across all Bayesian models, we defined a pre-specified infection classification rule. An individual was classified as “infected” (Outcome = 1) if it met at least one of the following criteria during the monitoring phase: (1) seroconversion as detected by competitive ELISA (cELISA), representing a robust immune response to exposure; (2) a positive RT-PCR result from nasal/ocular swabs, indicating active viral shedding; or (3) positive post-mortem PCR results from lymph or lung tissue in cases of mortality. While seroconversion involves a temporal lag of several days, we used a binary Bernoulli likelihood that is independent of this lag. This way, we compute the probability of a transmission event occurring strictly within the fixed exposure window (1–48 hours). Thus, the temporal delay in antibody detection affects the incubation period analysis but does not impact or bias the estimation of the unit-time transmission probability (p) nor the resulting minimum exposure time simulations.

**R4.5 & 4.6 Major Comments:** *5. A major strength of this study is the use of UWB-based indoor positioning to reconstruct high-frequency, metric inter-animal distances, which provides a much stronger basis for transmission modelling than binary contact/non-contact definitions. This is an important methodological contribution and deserves clearer emphasis in both the Results and Discussion. In particular, for the segmented and stratified transmission models (Transmission probability models; Table 5, approximately pp. 18–19and pp. 28–29), the manuscript would benefit from explicitly identifying which distance intervals contribute most strongly to estimated infection risk, and from interpreting these intervals in biologically plausible terms (for example, direct contact, short-range droplet/aerosol exposure, or shared airspace effects).*

*6. A major strength of this study is the use of UWB-based indoor positioning to reconstruct high-frequency, metric inter-animal distances, which provides a stronger foundation for transmission modelling than binary contact/non-contact definitions. This methodological advantage should be emphasized more clearly in the Results and Discussion. More importantly, the manuscript would be strengthened by linking model outputs more explicitly to biologically plausible transmission mechanisms. For the segmented and stratified models, the authors should clarify which distance intervals contribute most to infection risk and interpret these ranges in terms of likely mechanisms (e.g., direct contact, short-range droplet/aerosol exposure, or shared airspace).*

*For the envelope model, the biological meaning of key parameters (particularly P₀ and λ) should be explained more directly, including how rapidly risk declines with distance and what near-zero-distance probability represents in real animal interactions.*

*Likewise, for the density-dependent minimal exposure time estimates, the Discussion would benefit from translating the results into practical scenarios (e.g., lower-density free-grazing versus higher-density pen or market settings). These revisions would improve the biological interpretability and applied relevance of the modelling results without requiring substantial changes to the analytical framework.*

**Response:** We thank the reviewer for this positive feedback.

We revised the Discussion and added a paragraph to make the UWB contribution more explicit.

**Lines 276-742 on Pages (38-39)**

A major strength of our study is the innovative use of UWB-based indoor positioning to reconstruct high-frequency, metric inter-animal distances. This provides a significantly stronger basis for transmission modeling compared to conventional binary contact/non-contact definitions, representing an important methodological advancement. This high-resolution spatial data allowed us to explore how proximity affects pathogen transmission. Our analysis of segmented and stratified models reveals that specific distance intervals contribute differentially estimated infection risk. For instance, very close proximity (e.g., <0.5m) likely represents direct contact transmission, involving physical touch or direct droplet transfer. Intermediate distances (e.g., 0.5-2m) could indicate short-range droplet/aerosol exposure, where infectious particles travel through air over limited distances. Longer distances (e.g., >2m) might reflect shared airspace effects or indirect transmission through contaminated fomites within a communal environment. Explicitly identifying and interpreting these intervals in biologically credible terms enhances the mechanistic understanding of PPRV spread. Similarly, for the envelope model, the parameters$p_{0}$ and $\lambda$ describe the baseline probability of transmission at near-zero distance and the rate at which this risk declines with increasing distance, respectively. A high $p_{0}$ signifies a strong risk upon close interaction, while a rapid decline in $\lambda$ indicates that transmission is highly distance-dependent.

We removed the previous text regarding exposure duration, as it read too much like a methodological description. In its place, we added a new paragraph that clearly interprets how our exposure-time estimates and contact density findings can directly inform outbreak modelling and the design of control strategies.

**Lines 793-803 on Page 41 (Section: Discussion)**

Our findings on minimal exposure time estimates provide actionable insights for outbreak modeling and control strategies. These results demonstrate that significantly less exposure time is required for transmission in scenarios with higher contact density. For example, the minimal exposure time can scale by a factor of four from a highly dense herd (distance < 0.5m, density = 5 animals per m^2^) to a herd that is almost 35 times less dense, or by a factor of 12 when animals move randomly in a 100 times larger space. This translates directly into practical scenarios: in lower-density free-grazing systems, animals might require longer periods of interaction for transmission to occur, whereas in higher-density pen or market settings, even brief encounters could pose a substantial risk. These findings strongly support the effectiveness of physical distancing measures and provide valuable insights into safety protocols in controlled environments.

**R4.7 Major Comment:** *What is the justification for using Ct < 26 to define infectious seeders (“good level of virus excretion…”, pp. 10 lines 177-183)? Please clarify whether this cutoff is supported by published infectivity data (PPRV or related morbilliviruses), assay calibration/virus isolation data, or internal validation in this study (e.g., correspondence with subsequent transmission events).*

**Response:** Following preliminary results of that test on samples 10 fold serial dilutions of the used PPRV, RNA extracted from the virus suspension at 103.2 TCID50/ml gave the threshold cycle number (CT value) of 22.13 while that result was 27.0 for the 100.2 TCID50/ml. Based on those standard samples results, once an inoculated animal expressed clinical signs of PPR and a good level of PPRV excretion in the collected swabs, CT value of the RT-PCR result less than 26, it was removed from paddock 2 to paddock 1 to serve as a seeder for the exposure phase. Usually, this period of experimental infections lasted between six to ten days post infection.

**Minor comments**

**R4.1 Minor Comment:** *Please briefly clarify in the Virus section why BTS cells (rather than CHS cells or other cell lines) were selected operationally (pp. 7, lines 108–114), particularly where unpublished data are cited.*

**Response:** We had in the laboratory both BTS and CHS cells in store in liquid nitrogen. Upon receipt of the suspected PPR samples from the field, we took 1 tube of each cell from the store for in vitro culture. Unfortunately, the CHS didn't grow well at that time compared to BTS cells that were finally used for the virus isolation. Later on, once enough CHS were obtained, the PPRV GP virus was grown also on those cells. But for the study, in which results are reported here, the virus that was grown only on BTS was used. Anyway, both cells, CHS and BTS, were developed by the same authors, Adombi and Diallo, at the IAEA laboratory in Vienna. The CHS cells were developed first and published, followed by BTS. Tests that were conducted in that laboratory had proved that both cells are efficient for PPRV isolation from pathological samples.

**R4.2 Minor Comment:** *Provide the full PyMC/BlackJax code and R scripts, plus an environment file (conda/requirements), random seeds, and convergence diagnostics summaries (ESS, divergences).*

**Response:** All code and data used in this study are publicly available on GitHub at: <https://github.com/mnhili/PPR_lineageIV_transmission>.
