# Supplementary material for: How fast can PPRV spread? Analyzing the results of an experimental PPRV infection of goats equipped with Ultra-Wideband sensors
Source: PLoS One. 2026 Sep 23;21(9):e0358839. doi: 10.1371/journal.pone.0358839 (PMC13600536; doi:10.1371/journal.pone.0358839)
Supplement: Peer review history

# Editor report: Decision Letter 0

**DOI:** 10.1371/journal.pone.0358839.r001

**Authors:** Yogesh Chander

14 May 2026

PONE-D-26-21306

How fast can PPRV spread? Analyzing the results of an experimental PPRV infection of goats equipped with Ultra-WideBand sensors

PLOS One

Dear Dr. Nhili,

Thank you for resubmitting your manuscript, "How fast can PPRV spread? Analyzing the results of an experimental PPRV infection of goats equipped with Ultra-WideBand sensors," to PLOS ONE.

As you are aware, this manuscript (previously PONE-D-25-40904) was withdrawn just as an editorial decision was being finalized. Because this is a new submission (PONE-D-26-21306), the system typically requires a fresh round of peer review. However, as the handling Academic Editor, I have reviewed your resubmission and compared it with the original version. I note that the scientific content remains unchanged, and you have addressed the data availability by providing a DOI.

To ensure efficiency and maintain continuity, I have decided to proceed with the existing expert reviews obtained during the first submission. All four reviewers found the work methodologically impressive and significant for PPRV eradication efforts, though they have raised several points regarding clarity, statistical interpretation, and manuscript structure. Therefore, I am inviting you to submit a revised version of the manuscript that addresses the compiled reviewer comments below.

**Reviewer 1 (Reviewer Recommendation Term: Minor Revision)**

This is a new, methodologically impressive piece of work that combines an experimental PPRV infection in goats with tracking based on Ultra-wideband (UWB) sensors to quantify contact patterns and transmission dynamics modelling using a Bayesian framework. The fusion of behavioural and epidemiological data is a breakthrough, and it helps to illuminate the concepts of exposure time, R₀, and incubation period for PPRV lineage IV under laboratory conditions.

The research is of great importance and fits well with the global PPR eradication initiatives. It is, however, a little too detailed in some parts of the manuscript, especially the Introduction and Materials and Methods, where the background and technical descriptions could be shortened significantly to make the text more reader friendly. The Results are detailed and thorough but sometimes they are too descriptive. The authors could clarify more and interpret less by focusing on the epidemiological implications of contact behaviour and exposure time which would help to unfold the story.

The Discussion brings forward the results in the context of existing literature and would be improved by adding a brief limitations section and a more definite statement regarding the importance of the findings for field surveillance and control programs. In essence, the work is scientifically robust and makes a novel addition to the body of knowledge, but a moderate revision as indicated below will improve it further.

1. Abstract: Shorten methodological detail; emphasize novelty and main findings instead of listing all numerical results. Add a clear concluding statement linking findings to implications for PPRV control and spread.
2. Introduction: Condense the long epidemiological background (especially PPR history and lineage distribution). Move the study rationale and objectives earlier for clearer focus. Merge overlapping statements on lineage IV emergence and relevance to Senegal.
3. Materials and Methods: Streamline repetitive procedural descriptions (e.g., infection, exposure, monitoring). Move excessive manufacturer, equipment, and coordinate details to supplementary material. Summarize UWB system setup focus on performance (accuracy, sampling rate) rather than hardware specifications. Add a schematic summarizing the modelling framework (Baseline →Segmented → Stratified → Envelope). Ensure all equations are properly formatted and numbered consistently.
4. Simplify numeric descriptions use summary terms (“higher,” “longer,” “comparable”) instead of repeating raw values. Strengthen interpretative commentary linking behavioural contact data to transmission outcomes. Verify figure order and in-text citations for consistency. Consider merging Figures 4 and 5 or moving detailed distribution plots to supplementary materials.
5. Discussion: Begin with a strong interpretative paragraph highlighting how findings advance current understanding of PPRV spread. Add a short limitations paragraph (e.g., small sample size, single strain, confined experimental setting). Clarify how exposure-time estimates and contact density findings could inform outbreak modelling and control strategies. Remove repetitive restatement of numerical results already covered in Results.

**Reviewer 2 (Reviewer Recommendation Term: Major Revision)**

This manuscript investigated PPRV transmission dynamics in goats using Ultra-WideBand proximity sensors combined with Bayesian modelling. The outcomes are innovative and relevant to PPR epidemiology and global eradication efforts. The study provides estimates of transmission rate, R₀, incubation period, and explores the influence of proximity on transmission probability.

The research design is interesting, and the analytical framework is mostly appropriate. However, several important issues require clarification before the manuscript can be considered for publication.

1. The author should clearly mention the study objectives
2. Describe the justification of assumptions used for R₀ estimation (particularly the infectious period)
3. Explain clearly how “minimum exposure time” was inferred, given the experimental results
4. Discuss the justification of Bayesian model structure

Overall, the study has strong potential, but substantial revisions are required to improve clarity, methodological rigor, and interpretation of findings.

**Reviewer 3 (Reviewer Recommendation Term: Minor Revision)**

**General Assessment** : It is the first study combining

- Controlled PPRV transmission experiments
- Real time UWB proximity tracking
- Bayesian transmission modelling
- The integration of contact heterogeneity into transmission inference is scientifically important.
- Addresses a key gap in PPR eradication modelling where field outbreak data are sparse.
- In the experimental control, it has defined a good format of: seeder infection, controlled exposure and individual monitoring
- There is pre-screening of animals which validates the findings

**Comments** : see the attached file from this reviewer.

**Reviewer 4 (Reviewer Recommendation Term: Major Revision)**

In this manuscript, Nhili et al. present an experimental PPRV infection study in goats using the Senegal 20-GP strain (lineage IV), combining Ultra-WideBand (UWB) proximity tracking with Bayesian modelling to estimate transmission-related parameters, including the transmission rate, R₀, incubation period, distance-dependent transmission probability, and minimum exposure time under different density conditions. The study is methodologically strong and addresses an important gap in veterinary epidemiology. In particular, the use of UWB-derived high-resolution spatial and temporal data provides a substantially more informative basis for transmission modelling than conventional homogeneous-mixing assumptions or simplified contact definitions. The experimental setup also appears carefully executed, with appropriate animal screening, quarantine, biosafety, and welfare procedures, and the analytical framework makes good use of the granularity of the proximity data rather than collapsing contacts into binary events. This work has clear relevance for improving parameterization of transmission models used in PPR control and eradication planning.

However, several points should be clarified and strengthened before the manuscript can be considered for publication. In particular, the paper would benefit from a clearer statement of the primary research questions and underlying hypotheses. In this context, the introduction (pp. 5, lines 65–68) should briefly summarize the key findings of Herzog et al., which appears to be the most relevant prior study for framing the present work. This would help clarify how the current experiment differs from Herzog et al., what gap it addresses, and where its main novelty lies. The manuscript would also benefit from a more coherent and accessible presentation of the statistical modelling framework, as well as a more explicit discussion of uncertainty, sample size limitations, and parameter identifiability. In addition, the biological and practical interpretation of the distance- and density-dependent findings should be expanded so that their implications for transmission dynamics and disease control are easier to assess.

**Major comments**

1. The Introduction provides useful background on PPR and lineage IV and explains that the manuscript is structured in two parts (classical epidemiological parameters and contact-pattern-based models), but the main research questions and hypotheses are not stated clearly enough. I recommend adding a short paragraph at the end of the Introduction (e.g., after the paragraph beginning “In our study, we analysed data from experimental infections…” on pp. 6, lines85–95) to explicitly clarify the main research questions and hypotheses by stating the primary objectives (e.g., estimation of β and R₀, characterization of the incubation period, quantification of distance- and time-dependent transmission, and estimation of minimal exposure times under different density conditions), the secondary or exploratory objectives (e.g., assessing the added value of UWB distance information relative to duration-only models), and the main a priori expectations. This would make the study design easier to follow and help readers interpret the modelling results more directly.
2. The “Statistical analysis and models” section (beginning around pp. 14) includes several important components (transmission rate/R₀, incubation period, and the baseline/segmented/stratified/envelope models), but it remains dense for non-specialist readers. Although Tables 4 and 5 summarize priors and posterior estimates, they do not provide a clear model-level roadmap. I recommend adding a brief overview table or schematic at the start of this section summarizing, for each model, the outcome, predictors, likelihood, key parameters (e.g., βₕ, β, R₀, p, P₀, λ, αᵢ), and epidemiological interpretation. The notation should also be made fully consistent across subsections, particularly for βₕ versus β, p versus P versus P₀, and αᵢ/dᵢ/tᵢ/λ/P₀ (pp. 14-18). In addition, the Results section should consistently state which model is being used for each estimate (e.g., Table 5, pp. 28–29) and briefly restate the key assumptions (distance bands vs. continuous envelope). As currently presented, Table 5 combines posterior estimates from different model classes, but the accompanying text does not always restate the relevant assumptions, making it easy to compare p estimates across models even when their definitions differ.
3. Some technical details could be moved to the Supplementary Material to improve the flow of the main text without compromising reproducibility. In particular, this includes the Jaccard-based “era” clustering in the “Network analysis of the contact pattern” subsection (pp. 13, lines 247-266) and the full envelope-model formula (pp. 19). A short conceptual description can remain in the main text, while the full technical formulation is moved to the Supplementary Material.
4. This study appears to use serology as the principal infection endpoint in most quantitative analyses, with RT-PCR results and clinical signs incorporated primarily as complementary evidence. This approach is acceptable; however, it would benefit from more explicit statement and consistent application throughout the manuscript. To improve clarity and reproducibility, the authors are encouraged to add a brief paragraph in the “Statistical analysis and models” section (pp. 14) specifying the primary endpoint used in each analysis, defining a prespecified infection classification rule (e.g., serology and/or RT-PCR, including post-mortem PCR where applicable), and briefly clarifying the biological interpretation of each endpoint (e.g., prior infection/seroconversion, viral shedding, clinical disease). In addition, because seroconversion occurs after infection, the authors should clarify how this temporal lag was addressed in models estimating minimal exposure time (most likely in the exposure-threshold or proximity-based modelling subsections) and provide a cross-reference to the relevant model paragraph.
5. A major strength of this study is the use of UWB-based indoor positioning to reconstruct high-frequency, metric inter-animal distances, which provides a much stronger basis for transmission modelling than binary contact/non-contact definitions. This is an important methodological contribution and deserves clearer emphasis in both the Results and Discussion. In particular, for the segmented and stratified transmission models (Transmission probability models; Table 5, approximately pp. 18–19and pp. 28–29), the manuscript would benefit from explicitly identifying which distance intervals contribute most strongly to estimated infection risk, and from interpreting these intervals in biologically plausible terms (for example, direct contact, short-range droplet/aerosol exposure, or shared airspace effects).
6. A major strength of this study is the use of UWB-based indoor positioning to reconstruct high-frequency, metric inter-animal distances, which provides a stronger foundation for transmission modelling than binary contact/non-contact definitions. This methodological advantage should be emphasized more clearly in the Results and Discussion. More importantly, the manuscript would be strengthened by linking model outputs more explicitly to biologically plausible transmission mechanisms. For the segmented and stratified models, the authors should clarify which distance intervals contribute most to infection risk and interpret these ranges in terms of likely mechanisms (e.g., direct contact, short-range droplet/aerosol exposure, or shared airspace). For the envelope model, the biological meaning of key parameters (particularly P₀ and λ) should be explained more directly, including how rapidly risk declines with distance and what near-zero-distance probability represents in real animal interactions. Likewise, for the density-dependent minimal exposure time estimates, the Discussionwould benefit from translating the results into practical scenarios (e.g., lower-density free-grazing versus higher-density pen or market settings). These revisions would improve the biological interpretability and applied relevance of the modelling results without requiring substantial changes to the analytical framework.
7. What is the justification for using Ct < 26 to define infectious seeders (“good level of virus excretion…”, pp. 10 lines 177-183)? Please clarify whether this cutoff is supported by published infectivity data (PPRV or related morbilliviruses), assay calibration/virus isolation data, or internal validation in this study (e.g., correspondence with subsequent transmission events).

**Minor comments**

1. Please briefly clarify in the Virus section why BTS cells (rather than CHS cells or other cell lines) were selected operationally (pp. 7, lines 108–114), particularly where unpublished data are cited.
2. Provide the full PyMC/BlackJax code and R scripts, plus an environment file (conda/requirements), random seeds, and convergence diagnostics summaries (ESS, divergences).
3. Please provide a detailed point-by-point response to these comments along with your revised manuscript. We look forward to receiving your revision.

Please submit your revised manuscript by Jul 10 2026 11:59PM. If you will need more time than this to complete your revisions, please reply to this message or contact the journal office at plosone@plos.org. When you're ready to submit your revision, log on to https://www.editorialmanager.com/pone/ and select the 'Submissions Needing Revision' folder to locate your manuscript file.

Please include the following items when submitting your revised manuscript:

- A letter that responds to each point raised by the academic editor and reviewer(s). You should upload this letter as a separate file labeled 'Response to Reviewers'.
- A marked-up copy of your manuscript that highlights changes made to the original version. You should upload this as a separate file labeled 'Revised Manuscript with Track Changes'.
- An unmarked version of your revised paper without tracked changes. You should upload this as a separate file labeled 'Manuscript'.

If applicable, we recommend that you deposit your laboratory protocols in protocols.io to enhance the reproducibility of your results. Protocols.io assigns your protocol its own identifier (DOI) so that it can be cited independently in the future. For instructions see: https://journals.plos.org/plosone/s/submission-guidelines#loc-laboratory-protocols. Additionally, PLOS ONE offers an option for publishing peer-reviewed Lab Protocol articles, which describe protocols hosted on protocols.io. Read more information on sharing protocols at https://plos.org/protocols?utm_medium=editorial-email&utm_source=authorletters&utm_campaign=protocols.

As the corresponding author, your ORCID iD is verified in the submission system and will appear in the published article. PLOS supports the use of ORCID, and we encourage all coauthors to register for an ORCID iD and use it as well. Please encourage your coauthors to verify their ORCID iD within the submission system before final acceptance, as unverified ORCID iDs will not appear in the published article. *Only*  the individual author can complete the verification step; PLOS staff *cannot*  verify ORCID iDs on behalf of authors.

We look forward to receiving your revised manuscript.

Kind regards,

Yogesh Chander, Ph.D.

Academic Editor

PLOS One

Journal Requirements:

When submitting your revision, we need you to address these additional requirements.

1. Please ensure that your manuscript meets PLOS ONE's style requirements, including those for file naming. The PLOS ONE style templates can be found at

https://journals.plos.org/plosone/s/file?id=wjVg/PLOSOne_formatting_sample_main_body.pdf and

https://journals.plos.org/plosone/s/file?id=ba62/PLOSOne_formatting_sample_title_authors_affiliations.pdf

2. To comply with PLOS One submissions requirements, in your Methods section, please provide additional information regarding the experiments involving animals and ensure you have included details on (1) methods of sacrifice, (2) methods of anesthesia and/or analgesia, and (3) efforts to alleviate suffering.

3. We note that the grant information you provided in the ‘Funding Information’ and ‘Financial Disclosure’ sections do not match.

When you resubmit, please ensure that you provide the correct grant numbers for the awards you received for your study in the ‘Funding Information’ section.

4. Your ethics statement should only appear in the Methods section of your manuscript. If your ethics statement is written in any section besides the Methods, please move it to the Methods section and delete it from any other section. Please ensure that your ethics statement is included in your manuscript, as the ethics statement entered into the online submission form will not be published alongside your manuscript.

5. If the reviewer comments include a recommendation to cite specific previously published works, please review and evaluate these publications to determine whether they are relevant and should be cited. There is no requirement to cite these works unless the editor has indicated otherwise.

[Note: HTML markup is below. Please do not edit.]

[NOTE: If reviewer comments were submitted as an attachment file, they will be attached to this email and accessible via the submission site. Please log into your account, locate the manuscript record, and check for the action link "View Attachments". If this link does not appear, there are no attachment files.]

To ensure your figures meet our technical requirements, please review our figure guidelines: https://journals.plos.org/plosone/s/figures

You may also use PLOS’s free figure tool, NAAS, to help you prepare publication quality figures: https://journals.plos.org/plosone/s/figures#loc-tools-for-figure-preparation.

NAAS will assess whether your figures meet our technical requirements by comparing each figure against our figure specifications.

Attachment Submitted filename: Reviewer 3_Comments.docx

# Author comment: Author response to Decision Letter 1

**DOI:** 10.1371/journal.pone.0358839.r002

8 Jul 2026

Dear Editor and Reviewers,

We sincerely appreciate the time and effort you have dedicated to reviewing our manuscript entitled “How fast can PPRV spread? Analyzing the results of an experimental PPRV infection of goats equipped with Ultra-Wideband sensors” (Manuscript ID: PONE-D-26-21306), and we are grateful for the constructive and insightful comments provided by the reviewers. Indeed, we have carefully considered all the comments and have revised the manuscript accordingly. We have addressed each point raised by the reviewers in a point-by-point response, which is provided below. Additionally, we have attached a clean version of the revised manuscript without any highlights for your convenience, and a revised manuscript with tracked changes to facilitate your review.

We believe that the revised manuscript has been substantially strengthened thanks to the feedback received, and we hope that it now meets the journal’s standards for publication.

Thank you once again for your consideration. We look forward to your favorable response and hope the revised manuscript can be accepted for publication in your esteemed journal.

Sincerely,

Manal NHILI

Email: manal.nhili@cirad.fr

Attachment Submitted filename: Response to Reviewers.docx

# Peer review: Decision Letter 1

**DOI:** 10.1371/journal.pone.0358839.r003

**Authors:** Yogesh Chander

27 Aug 2026

PONE-D-26-21306R1

How fast can PPRV spread? Analyzing the results of an experimental PPRV infection of goats equipped with Ultra-Wideband sensors

PLOS One?

Thank you for submitting your revised manuscript to *PLOS ONE* . The reviewers and I appreciate the extensive effort you have put into this revision, and we are very pleased with how well the study is shaping up.

I have noted the remaining points from the reviewers and am forwarding their comments for your consideration: please address the missing subject (<img height="20" src="data:image/png;base64,iVBORw0KGgoAAAANSUhEUgAAABUAAAAZCAMAAADpC5XfAAAAAXNSR0IArs4c6QAAAFpQTFRFAAAAAAAAAAA6AABmADo6ADqQAGa2OgAAOjqQOmaQOma2OpDbZgAAZrbbZrb/kDoAkNv/tmYAtmY6ttv/tv//25A625Bm2////7Zm/9uQ/9u2/9vb//+2///bw+n2hQAAAAF0Uk5TAEDm2GYAAAAJcEhZcwAAEnQAABJ0Ad5mH3gAAAAZdEVYdFNvZnR3YXJlAE1pY3Jvc29mdCBPZmZpY2V/7TVxAAAAnElEQVQoU81PURbCIAxrRcUpyhziQMf9r2laPrScwHyUR0iTQPQX2AIL9lfbpobDSu3ON0NXf8G9+qNhi6pGNrsH2MGhRdi+FxabL3qHKdkKYvsKNooo72a4ygCenqcVp9pS7m2LS31t01k0rEVMrZS5v8nGFsAWmBXkg26RTwksfIQdSopWP/WDFrGWxcsgu6SJg3hhPo/SUWTvH555B8W1cdxyAAAAAElFTkSuQmCC" width="17" />) in the Abstract, contextualize the 30-day quarantine recommendation as a point for further field validation, and clarify that <img height="20" src="data:image/png;base64,iVBORw0KGgoAAAANSUhEUgAAABUAAAAZCAMAAADpC5XfAAAAAXNSR0IArs4c6QAAAFpQTFRFAAAAAAAAAAA6AABmADo6ADqQAGa2OgAAOjqQOmaQOma2OpDbZgAAZrbbZrb/kDoAkNv/tmYAtmY6ttv/tv//25A625Bm2////7Zm/9uQ/9u2/9vb//+2///bw+n2hQAAAAF0Uk5TAEDm2GYAAAAJcEhZcwAAEnQAABJ0Ad5mH3gAAAAZdEVYdFNvZnR3YXJlAE1pY3Jvc29mdCBPZmZpY2V/7TVxAAAAnElEQVQoU81PURbCIAxrRcUpyhziQMf9r2laPrScwHyUR0iTQPQX2AIL9lfbpobDSu3ON0NXf8G9+qNhi6pGNrsH2MGhRdi+FxabL3qHKdkKYvsKNooo72a4ygCenqcVp9pS7m2LS31t01k0rEVMrZS5v8nGFsAWmBXkg26RTwksfIQdSopWP/WDFrGWxcsgu6SJg3hhPo/SUWTvH555B8W1cdxyAAAAAElFTkSuQmCC" width="17" /> values are model-derived estimates.

Please submit your revised manuscript by Sep 25 2026 11:59PM. If you will need more time than this to complete your revisions, please reply to this message or contact the journal office at plosone@plos.org. When you're ready to submit your revision, log on to https://www.editorialmanager.com/pone/ and select the 'Submissions Needing Revision' folder to locate your manuscript file.

Please include the following items when submitting your revised manuscript:

- A letter that responds to each point raised by the academic editor and reviewer(s). You should upload this letter as a separate file labeled 'Response to Reviewers'.
- A marked-up copy of your manuscript that highlights changes made to the original version. You should upload this as a separate file labeled 'Revised Manuscript with Track Changes'.
- An unmarked version of your revised paper without tracked changes. You should upload this as a separate file labeled 'Manuscript'.

If you would like to make changes to your financial disclosure, please include your updated statement in your cover letter. Guidelines for resubmitting your figure files are available below the reviewer comments at the end of this letter.

If applicable, we recommend that you deposit your laboratory protocols in protocols.io to enhance the reproducibility of your results. Protocols.io assigns your protocol its own identifier (DOI) so that it can be cited independently in the future. For instructions see: https://journals.plos.org/plosone/s/submission-guidelines#loc-laboratory-protocols. Additionally, PLOS One offers an option for publishing peer-reviewed Lab Protocol articles, which describe protocols hosted on protocols.io. Read more information on sharing protocols at https://plos.org/protocols?utm_medium=editorial-email&utm_source=authorletters&utm_campaign=protocols.

As the corresponding author, your ORCID iD is verified in the submission system and will appear in the published article. PLOS supports the use of ORCID, and we encourage all coauthors to register for an ORCID iD and use it as well. Please encourage your coauthors to verify their ORCID iD within the submission system before final acceptance, as unverified ORCID iDs will not appear in the published article. *Only*  the individual author can complete the verification step; PLOS staff *cannot*  verify ORCID iDs on behalf of authors.

We look forward to receiving your revised manuscript.

Kind regards,

Yogesh Chander, Ph.D.

Academic Editor

PLOS One

Journal Requirements:

1. If the reviewer comments include a recommendation to cite specific previously published works, please review and evaluate these publications to determine whether they are relevant and should be cited. There is no requirement to cite these works unless the editor has indicated otherwise.

2. Please review your reference list to ensure that it is complete and correct. If you have cited papers that have been retracted, please include the rationale for doing so in the manuscript text, or remove these references and replace them with relevant current references. Any changes to the reference list should be mentioned in the rebuttal letter that accompanies your revised manuscript. If you need to cite a retracted article, indicate the article’s retracted status in the References list and also include a citation and full reference for the retraction notice.

[Note: HTML markup is below. Please do not edit.]

Reviewers' comments:

Reviewer's Responses to Questions

**Comments to the Author**

Reviewer #1: All comments have been addressed

Reviewer #2: (No Response)

**********

2. Is the manuscript technically sound, and do the data support the conclusions?

Reviewer #1: Partly

Reviewer #2: Yes

**********

3. Has the statistical analysis been performed appropriately and rigorously?

Reviewer #1: Yes

Reviewer #2: Yes

**********

4. Have the authors made all data underlying the findings in their manuscript fully available?

The PLOS Data policy

Reviewer #1: Yes

Reviewer #2: Yes

**********

5. Is the manuscript presented in an intelligible fashion and written in standard English?

Reviewer #1: Yes

Reviewer #2: Yes

**********

Reviewer #1: Moderate recommendation to WOAH to be :"Our findings suggest that the adequacy of the current 21-day quarantine period deserves further evaluation and validation under field conditions." The conclusions should consistently specify: "for the lineage IV strain evaluated."; Discussion should emphasize more clearly that the R₀ = 4.3–8.6 were derived estimates, not directly observed values: Should explicitly mention that experimental contact

≠ natural contact. Some grammatical errors

Reviewer #2: The authors have substantially and conscientiously revised the manuscript in response to the previous review, and the revised version demonstrates genuine and thorough engagement with nearly every point raised. The clarity, structure, and scientific rigor of the paper have all improved markedly, and I consider the manuscript to be very close to being suitable for publication in PLOS ONE, pending a small number of remaining corrections detailed below.

Minor comments

1. A residual textual error remains in the Abstract, in the manuscript (page 2). The sentence, “Overall, was estimated between 4.3 (95% HDI: 3.1–4.8) and 8.6 (95% HDI: 6.2–9.6) depending on the infectious period...,” is missing its subject. The intended sentence appears to be: “Overall, R₀ was estimated to range from 4.3 ... to 8.6 ... depending on the infectious period.” This is likely an artifact of equation rendering during file conversion and should be corrected before typesetting, as the sentence is currently grammatically incomplete and may confuse readers who are unfamiliar with the study.

2. The recommendation to extend the quarantine period to at least 30 days, rather than the 21 days specified by the WOAH, is a relatively strong policy recommendation that appears in both the Abstract and the Discussion. Given that the estimated incubation period of 14.7 days has a fairly wide 95% credible interval (11.6–20.1 days), and that quarantine recommendations have important practical and economic implications for livestock movement control, the manuscript would be strengthened by explicitly acknowledging in the Discussion that this recommendation is based on a single experimental strain (Senegal 20-GP, lineage IV) evaluated under controlled conditions. Further validation across additional lineages or strains and under field conditions would be needed before this specific 30-day recommendation could be generalized as policy guidance.

The manuscript has been substantially strengthened, and virtually all major and minor comments from the previous review round have been thoroughly and appropriately addressed. Only the grammatical error in the Abstract and a brief caveat regarding the generalizability of the 30-day quarantine recommendation need to be addressed before the manuscript is suitable for acceptance.

**********

what does this mean?). If published, this will include your full peer review and any attached files.

If you choose “no”, your identity will remain anonymous but your review may still be made public.

**Do you want your identity to be public for this peer review?**  For information about this choice, including consent withdrawal, please see our Privacy Policy

Reviewer #1: No

Reviewer #2: No

**********

[NOTE: If reviewer comments were submitted as an attachment file, they will be attached to this email and accessible via the submission site. Please log into your account, locate the manuscript record, and check for the action link "View Attachments". If this link does not appear, there are no attachment files.]

To ensure your figures meet our technical requirements, please review our figure guidelines: https://journals.plos.org/plosone/s/figures

You may also use PLOS’s free figure tool, NAAS, to help you prepare publication quality figures: https://journals.plos.org/plosone/s/figures#loc-tools-for-figure-preparation.

NAAS will assess whether your figures meet our technical requirements by comparing each figure against our figure specifications.

Attachment Submitted filename: The transmission of PPR.docx

# Author comment: Author response to Decision Letter 2

**DOI:** 10.1371/journal.pone.0358839.r004

27 Aug 2026

Dear Editor and Reviewers,

We thank you for your careful review of our revised manuscript entitled “How fast can PPRV spread? Analyzing the results of an experimental PPRV infection of goats equipped with Ultra-Wideband sensors” (Manuscript ID: PONE-D-26-21306R1). We have addressed all of the minor comments in the revised manuscript.

The Abstract has been revised to explicitly identify R0 as the subject of the relevant sentence and to clarify that the reported values are derived estimates based on infectious-period assumptions from previous literature. The previous recommendation to extend quarantine to 30 days has also been moderated. The revised text now states that the adequacy of the current 21-day quarantine period specified in the WOAH Terrestrial Code warrants further evaluation and validation under field conditions, rather than implying an immediate policy revision.

The Discussion has been expanded to acknowledge the use of a single experimental PPRV strain, Senegal 20-GP, belonging to lineage IV, and to clarify that controlled experimental contact conditions may differ from natural contact patterns in pastoral systems, markets, and communal grazing. The need for further validation across additional strains or lineages and under field conditions is also stated. The Conclusion has been revised accordingly to clarify the scope and generalizability of our findings.

All corresponding changes have been incorporated into the revised manuscript. Given the minor and closely related nature of these comments, we have summarized the revisions rather than providing a separate point-by-point response.

We believe that the revised manuscript has been substantially strengthened thanks to the feedback received, and we hope that it now meets the journal’s standards for publication.

Thank you once again for your consideration. We look forward to your favorable response and hope the revised manuscript can be accepted for publication in your esteemed journal.

Sincerely,

Manal NHILI

Email: manal.nhili@cirad.fr

Corresponding Author

Attachment Submitted filename: Response to Reviewers.pdf

# Editor report: Decision Letter 2

**DOI:** 10.1371/journal.pone.0358839.r005

**Authors:** Yogesh Chander

7 Sep 2026

How fast can PPRV spread? Analyzing the results of an experimental PPRV infection of goats equipped with Ultra-Wideband sensors

PONE-D-26-21306R2

Dear Dr. Nhili,

Thank you for submitting your revised manuscript, **PONE-D-26-21306R2** , entitled *"How fast can PPRV spread? Analyzing the results of an experimental PPRV infection of goats equipped with Ultra-Wideband sensors,"*  to *PLOS ONE* .

As you know, publication in *PLOS ONE*  requires strict adherence to our core criteria: rigorous methodology, complete data accessibility, and high scientific validity without overstating experimental findings. In the first round, four independent reviewers examined the study in detail and raised a substantial number of scientific and methodological concerns. Following your comprehensive revision, two of our reviewers returned for a second closer reading and this time focusing on the finer points of interpretation, including the framing of the R0 estimates, the scope of your quarantine-period recommendation, and the distinction between experimental and natural contact conditions.

This manuscript was submitted last year as well and **unfortunately**  before coming to any decision it was withdrawn and again this year you submitted and it went through a rigorous review.

Following this final, exhaustive assessment of your revised text and response: I am satisfied that every remaining scientific, technical, and editorial concern has been fully resolved. The integration of Ultra-Wideband tracking with Bayesian transmission modeling provides a valuable, scientifically sound contribution to understanding PPRV dynamics. Therefore, I would like to inform you that your manuscript is suitable for publication and will be formally accepted for publication once it meets all outstanding technical requirements.

Within one week, you’ll receive an e-mail detailing the required amendments. When these have been addressed, you’ll receive a formal acceptance letter, and your manuscript will be scheduled for publication.

An invoice will be generated when your article is formally accepted. Please note, if your institution has a publishing partnership with PLOS and your article meets the relevant criteria, all or part of your publication costs will be covered. Please make sure your user information is up-to-date by logging into Editorial Manager at Editorial Manager® and clicking the ‘Update My Information' link at the top of the page. For questions related to billing, please contact billing support.

If your institution or institutions have a press office, please notify them about your upcoming paper to help maximize its impact. If they’ll be preparing press materials, please inform our press team as soon as possible -- no later than 48 hours after receiving the formal acceptance. Your manuscript will remain under strict press embargo until 2 pm Eastern Time on the date of publication. For more information, please contact onepress@plos.org.

Thanks for completing a thorough review process.

Sincerely,

**Dr. Yogesh Chander**

Academic Editor

*PLOS ONE*

Additional Editor Comments (optional):

Reviewers' comments:

# Editor report: Acceptance letter

**DOI:** 10.1371/journal.pone.0358839.r006

**Authors:** Yogesh Chander

PONE-D-26-21306R2

PLOS One

Dear Dr. Nhili,

I'm pleased to inform you that your manuscript has been deemed suitable for publication in PLOS One. Congratulations! Your manuscript is now being handed over to our production team.

At this stage, our production department will prepare your paper for publication. This includes ensuring the following:

* All references, tables, and figures are properly cited

* All relevant supporting information is included in the manuscript submission,

* There are no issues that prevent the paper from being properly typeset

You will receive further instructions from the production team, including instructions on how to review your proof when it is ready. Please keep in mind that we are working through a large volume of accepted articles, so please give us a few days to review your paper and let you know the next and final steps.

Lastly, if your institution or institutions have a press office, please let them know about your upcoming paper now to help maximize its impact. If they'll be preparing press materials, please inform our press team within the next 48 hours. Your manuscript will remain under strict press embargo until 2 pm Eastern Time on the date of publication. For more information, please contact onepress@plos.org.

You will receive an invoice from PLOS for your publication fee after your manuscript has reached the completed accept phase. If you receive an email requesting payment before acceptance or for any other service, this may be a phishing scheme. Learn how to identify phishing emails and protect your accounts at https://explore.plos.org/phishing.

If we can help with anything else, please email us at customercare@plos.org.

Thank you for submitting your work to PLOS One and supporting open access.

Kind regards,

PLOS One Editorial Office Staff

on behalf of

Dr. Yogesh Chander

Academic Editor

PLOS One
